# Single-subject detection of speech network activation using functional near-infrared spectroscopy

**DOI:** 10.1117/1.NPh.13.3.035009

**Published:** 2026-09-15

**Authors:** Carlotta Koch, Androu Abdalmalak, Joan Orpella, Jacob Rottenberg, Eric S. Jackson

**Affiliations:** a New York University, Department of Communicative Sciences and Disorders, New York, New York, United States; b New York University, Institute of Human Development and Social Change, New York, New York, United States; c New York University, Department of Psychology, New York, New York, United States; d Western University, Schulich School of Medicine and Dentistry, Department of Physiology and Pharmacology, London, Ontario, Canada; e Synapse NeuroAnalytics Inc., London, Ontario, Canada; f Georgetown University Medical Center, Department of Neuroscience, Washington, District of Columbia, United States; g Columbia University, Department of Applied Physics and Applied Mathematics, New York, New York, United States

**Keywords:** fNIRS, single-subject analysis, speech production, speech perception, auditory processing

## Abstract

**Significance:**

Most neuroimaging studies rely on group-level analyses, yet many clinical and translational applications require reliable inference at the level of individual participants.

**Aim:**

We evaluated whether functional near-infrared spectroscopy (fNIRS) can detect expected speech network related activation patterns at the single-subject level.

**Approach:**

Twenty-seven adults completed simple listening and speaking tasks while fNIRS data were recorded over bilateral frontal, temporal, and parietal regions. Data were analyzed using general linear models with false discovery rate correction and physiologically informed criteria, including expected HbO/HbR directionality and cross-chromophore coupling.

**Results:**

Group-level analyses revealed activation patterns consistent with prior literature, with widespread HbO increases and HbR decreases. At the single-subject level, most participants showed task-related activation within predefined cortical regions, although the number and spatial distribution of significant channels varied. Speaking elicited more robust and consistent activation than listening. HbO and HbR responses were positively associated across channels, indicating coherent neurovascular coupling.

**Conclusions:**

fNIRS can detect physiologically plausible activation patterns at the individual level, even in brief paradigms. However, variability across participants highlights the need to optimize task design and signal quality for reliable single-subject inference.

## 1 Introduction

Most neuroimaging methods reveal brain function at the group level, but in most clinical and translational settings, one size does not fit all. It is unclear to what extent canonical activation patterns can be reliably detected in individual neuroimaging datasets. Individual brain organization varies greatly and is often masked by group averages.^1^^–^^3^ This reliance on group-level averages becomes problematic in clinical contexts or when screening participants for inclusion in research, where individual variability is not averaged across participants but instead represents a potentially meaningful source of information that can guide diagnosis, treatment decisions, or participant selection.^2^^,^^4^^,^^5^ In this study, we address this gap using functional near-infrared spectroscopy (fNIRS) to evaluate whether expected activation patterns can be detected at the level of individual participants.

We focus on the cortical speech network using simple listen and speak tasks, given the potential for application of single-subject data in the field of speech-language pathology. These cortical networks are well characterized in the neuroimaging literature, mostly through group-level findings in functional magnetic resonance imaging (fMRI) and established neurocognitive models of speech perception and production.^6^^–^^10^ These networks are well established in the neuroimaging literature and are frequently investigated in research on speech disorders such as stuttering.^11^^–^^13^ Although fNIRS has demonstrated sensitivity to speech-related cortical activation at the group level,^14^^,^^15^ the reliability of detecting physiologically interpretable speech-related activation patterns at the single-subject level remains uncertain. In particular, it is unclear how consistently individual measurement channels capture spatially localized activation and show the expected coupling between HbO increases and HbR decreases. Prior work has reported substantial variability in replicability across labs,^16^^,^^17^ sessions and individuals.^18^^–^^20^ This variability is likely due to anatomical differences, optode placement variability, physiological noise, and fluctuations in task performance, which can strongly affect individual measurements but tend to average out in group-level analyses. Using simple listen and speak tasks offers a practical, brief, and easily implementable paradigm for probing this superficial cortical network in a manner that is compatible with potential clinical translation.

Listening and speaking involve distributed neural systems that coordinate activity across bilateral temporal, frontal, and parietal regions. During listening, activation is consistently observed across this large-scale network, including bilateral primary auditory cortex (Heschl’s gyrus) and superior temporal gyrus (STG).^21^^–^^23^ The middle temporal gyrus (MTG), particularly in the left hemisphere, contributes to lexical–semantic processing within a broader distributed network.^8^^,^^10^^,^^23^ Phonological processing, including the mapping of acoustic input onto phonemic representations, is more strongly associated with superior temporal regions, particularly the STG/superior temporal sulcus.^24^^,^^25^ Parietal regions such as the inferior parietal lobule, including the supramarginal gyrus, contribute to sensorimotor integration.^10^^,^^23^^,^^26^ Frontal cortical regions, including the inferior and middle frontal gyri, contribute to attentional control and top–down modulation of auditory processing during speech perception, while also supporting broader language-related and cognitive control functions.^23^^,^^27^^–^^30^ Motor-related regions such as the precentral gyrus, including the premotor cortex (PMC), and supplementary motor area (SMA) are also frequently co-activated during listening, potentially reflecting auditory–motor coupling or covert articulatory simulation; this network may also extend to inferior frontal regions implicated in speech motor planning.^27^^,^^28^^,^^31^^,^^32^ In addition, bilateral insular activation has been observed, likely due to its integrative role in processing auditory and interoceptive information.^23^^,^^33^ Together, these regions form a cohesive cortical network that supports auditory perception in both passive and active contexts.

**Fig. 1:**
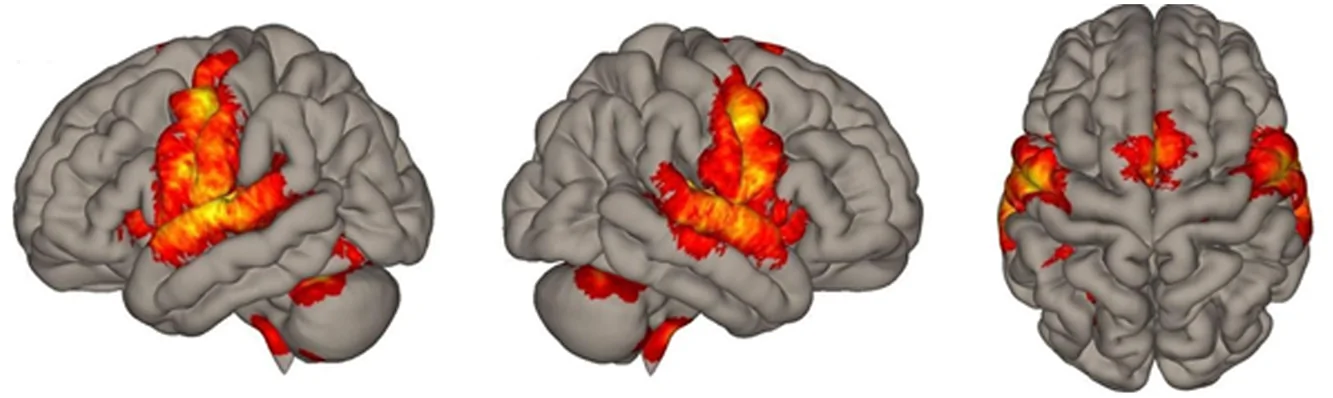
Cortical maps illustrating regions involved in speech production, based on functional neuroimaging data, shown from left to right: lateral view of the left hemisphere, lateral view of the right hemisphere, and superior view. Warmer colors indicate greater activation. Adapted from *Neural Control of Speech*^6^ with written permission from Frank H. Guenther. Copyright 2016 by MIT Press.

Speech production engages many of the same cortical regions, which can be seen in Fig. 1. Although subcortical structures, such as the cerebellum,^22^^,^^23^^,^^34^ also play an important role in speech production, the present study focuses on cortical regions because fNIRS primarily measures hemodynamic activity in the superficial cortex. The primary motor cortex drives articulatory execution and output by controlling the orofacial and laryngeal musculature,^6^^,^^22^ whereas the SMA and cingulate motor areas coordinate initiation of speech movements.^6^^,^^23^^,^^35^ The inferior frontal gyrus, including Broca’s area, is involved in articulatory planning and encoding,^6^^,^^23^^,^^35^ working in conjunction with the posterior superior temporal gyrus to monitor auditory feedback.^6^^,^^23^^,^^35^ These interacting components form a flexible and adaptive speech production network that exhibits stronger left-hemisphere dominance during fluent, multisyllabic production,^6^^,^^8^^,^^34^ yet allows for bilateral recruitment depending on linguistic and task demands.

Subcortical structures, such as the basal ganglia and thalamus, also play a critical role in speech production and listening. They are involved in motor control and speech initiation, contributing to the timing and sequencing of articulatory movements.^22^^,^^34^ However, these regions cannot be captured reliably using fNIRS and will therefore not be the focus of this paper.

Although fNIRS is limited to measuring hemodynamic activity in superficial cortical layers, these regions include many key nodes of the speech and auditory networks, and the modality has demonstrated the capacity to capture activation patterns that are comparable in validity and reliability to those obtained with fMRI when targeting cortex accessible to both methods.^14^^,^^15^^,^^36^ fNIRS is a noninvasive neuroimaging technique that uses near-infrared light to quantify task-evoked changes in cerebral hemodynamics.^37^ Importantly, its quiet, portable hardware and relative tolerance to motion compared with fMRI and electroencephalography make fNIRS particularly well suited for overt speech paradigms, where jaw and head movement are difficult to eliminate.^38^^,^^39^ In addition, fNIRS allows participants to sit upright and engage in face-to-face interaction, supporting more naturalistic speaking and listening contexts and increasing ecological validity. These practical advantages have facilitated its application in populations that may find traditional scanning environments challenging, including individuals with speech and language impairments^38^ and infants and children in developmental research.^40^ Collectively, these features underscore the translational potential of fNIRS for individualized and clinically relevant speech research.

fNIRS measures task-evoked changes in cerebral hemodynamics by emitting near-infrared light into the scalp and quantifying absorption differences between oxygenated (HbO) and deoxygenated hemoglobin (HbR) at spatially separated detectors. It specifically tracks changes in oxygenated hemoglobin (HbO) and deoxygenated hemoglobin (HbR).^15^^,^^41^^,^^42^ Evaluating single-subject fNIRS data requires not only statistical significance but also physiologically plausible response patterns, including appropriate directionality of HbO and HbR responses. By contrast, fMRI measures the blood-oxygenation-level-dependent (BOLD) signal, which reflects changes in only deoxyhemoglobin.^14^ Measuring both HbO and HbR can improve signal interpretation by helping distinguish true neural responses from artifacts or systemic noise.^38^ However, HbO and HbR differ in their temporal dynamics and sensitivity to physiological noise, which may affect their interpretability in brief, event-related paradigms. Although fNIRS is limited to superficial cortical regions and has lower spatial resolution than fMRI,^14^^,^^36^^,^^42^ its higher temporal resolution and portability make it particularly well suited for measuring cortical activity in clinical populations.^43^ These features also support applications that require adaptive or repeated measurements within subjects, including real-time neurofeedback and brain-computer interfaces,^44^^–^^46^ where robust, interpretable signals must be obtained at the individual level and in real time.

fNIRS does not work equally well for all individuals. Many studies have reported challenges in obtaining consistent hemodynamic responses across participants.^16^^,^^17^^,^^47^ Poor signal quality can render individual data unusable and, when present in even a few participants, may also distort group-level results despite careful preprocessing.^16^^,^^48^ The validity of the neural signal cannot be confirmed based on the raw signal alone, as good signal strength during acquisition does not guarantee meaningful task-evoked responses. Key preprocessing steps, such as motion correction, signal filtering, and other methods to remove extracerebral noise, are necessary to evaluate whether the recorded activity reflects true cortical activation.^17^^,^^37^^,^^47^^,^^49^ In the canonical hemodynamic response, HbO increases while HbR decreases in the same regions; this negative correlation reflects neurovascular coupling. When a brain area becomes active, it requires increased oxygen delivery, leading to increased blood flow and corresponding changes in hemoglobin concentrations. Consistent HbO–HbR coupling therefore provides an important physiological indicator that the measured signal reflects genuine cortical activation rather than noise.

A growing number of studies have used fNIRS to assess general speech production and a diverse range of speech disorders. For example, previous work has aimed to dissociate speech planning from execution, using nonword repetition and overt versus covert naming, in adults who stutter (AWS)^50^ and children who stutter (CWS).^51^ Other paradigms include connected speech tasks such as describing visual scenes in full sentences after a go-signal for CWS,^52^ as well as reading, counting, and free speech in AWS.^53^ In poststroke aphasia, fNIRS has been used to measure hemodynamic changes during confrontation naming, counting, and free narrative tasks.^54^ One study in developmental dyslexia used a passive listening paradigm in which participants heard amplitude-modulated white noise presented at varying temporal rates.^55^ General speech perception paradigms have also been applied in participants without speech disorders. For example, some studies used passive story listening to decode which segment of a story was perceived,^56^ whereas others presented sentences under varying conditions—quiet, noise, or vocoded speech—followed by overt repetition to examine neural activation during both speech perception and production.^57^ These paradigms demonstrate the growing utility of fNIRS for investigating speech and language disorders and highlight its potential for clinical and translational applications.

However, most neuroimaging research relies on group-level analyses using mass-univariate approaches such as the General Linear Model (GLM), which estimate average activation patterns across participants. Although statistically powerful and widely implemented, this framework is optimized for detecting effects that are consistent at the population level rather than characterizing heterogeneity across individuals.^1^^,^^2^ As a result, activation patterns that are meaningful at the level of a single participant may be obscured when averaged across subjects. This limitation becomes particularly salient in clinical contexts, where inference about an individual is required. Despite growing interest in individual-level inference,^4^ relatively few studies have systematically evaluated physiologically interpretable speech-related activation patterns at the level of individual participants and channels using standard fNIRS analysis frameworks. For example, some studies have used individualized functional channels of interest to examine cortical responses at the level of individual participants,^58^ whereas others have applied feature-learning and classification frameworks to distinguish hemodynamic patterns between individuals.^59^ More recent work has additionally evaluated within-subject reproducibility of fNIRS responses across sessions.^60^ These studies demonstrate the growing feasibility of individualized fNIRS analysis, but they address different goals than the present study, such as classification performance, developmental localization, or test–retest reproducibility. By contrast, the current study focuses specifically on whether a simple speech and listening paradigm can produce physiologically interpretable activation patterns at the level of individual participants and channels using standard GLM-based analysis approaches.

Our study was designed to further evaluate speech-related activation patterns at the individual participant level by implementing a simple yet informative paradigm to map speech and auditory network activation. Rather than focusing on classification or individualized localization alone, the present study specifically examined whether canonical activation patterns and physiologically plausible HbO/HbR coupling could be detected consistently within individual participants. This approach enhances the interpretability of single-subject data and supports clinical and translational applications, such as developmental speech monitoring and assessment of speech and language disorders. Because direct comparison between single-subject data and group-level findings is methodologically not straightforward—and most established results derive from group-level studies—we additionally performed group-level analyses to confirm that our paradigm produces activation patterns consistent with prior research. The primary aim of this study was to assess whether expected activation patterns can be detected at the level of individual participants using fNIRS.

## 2 Materials and Methods

### 2.1 Participants

A total of 27 adults were recruited through the first author’s personal network and convenience sampling. Prior to participation, all individuals were provided with detailed information about the study’s purpose and procedures, and written informed consent was obtained in accordance with the guidelines of the New York University Institutional Review Board.

No formal exclusion criteria were specified; however, all participants verbally reported normal or corrected-to-normal vision and no history of neurological or psychiatric disorders. All participants were included in the final analysis. Participants ranged in age from 19 to 64 years (M=29.04, SD=10.91). Of the 27 participants, 20 identified as female, 22 were right-handed, and 18 were native speakers of English. All participants were fluent in English, based on self-report and informal assessment by the experimenter through conversation at the beginning of the session. No monetary compensation was provided for participation.

### 2.2 Design

This study aimed to assess whether a brief, low-demand listen and speak paradigm administered with functional near-infrared spectroscopy (fNIRS) can reliably elicit expected patterns of cortical activation at the single-subject level. Specifically, we examined whether single-subject activation during a simple listen task and a simple speak task aligned with previously established findings on auditory and speech networks. To support interpretation of individual results, we also conducted a group-level analysis to confirm that the paradigm elicits consistent activation patterns at the group level.

Participants completed the same two tasks: (1) a listen task and (2) a speak task in a single session in fixed order. Both tasks used the same set of 50 stimuli, consisting of 25 trisyllabic English words presented twice in each task. All stimuli were phonotactically legal in American English and followed canonical stress patterns with a single primary stress per word. No item contained atypical stress assignment or irregular prosodic structure. Phonological complexity was controlled by excluding rare consonant clusters and unusual phoneme sequences. This ensured comparable articulatory length and prosodic structure across trials. A complete word list can be found in Table S1 in the Supplementary Material.

In the Listen Task, words were presented auditorily, and participants were instructed to listen passively. In the Speak Task, the same words appeared visually on a screen, and participants were instructed to read them aloud. Each word was separated by a baseline period to allow for signal resolution. No experimental conditions or group comparisons were included.

### 2.3 Procedure

All procedures were conducted in a quiet testing room at the NYU Department of Communicative Sciences and Disorders. Participants completed a single lab visit lasting ∼90  min. Upon arrival, participants provided written informed consent. The researcher then measured the participant’s head size and fitted the fNIRS cap containing light sources and detectors positioned over cortical regions relevant for auditory and speech processing. The cap was positioned using standard anatomical landmarks following the international 10 to 20 system. Cz was identified as the midpoint between the nasion and inion and verified by measuring the distance from both preauricular points to ensure symmetrical placement across participants. The cap was stabilized using a chest strap to reduce cap displacement associated with jaw and head movement during speaking. A chest strap was preferred over a chin strap because it can be assumed that chin straps can introduce additional mechanical artifacts by directly coupling jaw movement to the cap and optodes during articulation. After sensor calibration, participants completed the two experimental tasks while seated in front of a monitor.

In the Listen Task, participants heard the words, presented one at a time, through headphones at a comfortable volume level for the participant, with a 15-s rest interval between each word. They were instructed to remain still and attend to each word without responding. In the Speak Task, the same set of words appeared visually on a screen, one at a time. Participants were instructed to say each word aloud immediately upon its appearance. A 15-s rest period was included between trials. Each task lasted ∼12  min.

Throughout the session, participants were asked to focus on a central fixation cross in between trials and minimize movement. A researcher remained present in the room to monitor comfort and data quality. Following the tasks, a 3D scan of each participant’s head shape was captured using a mobile scanning application to enable digitization of optode locations and anatomical landmarks (nasion, left, and right preauricular points). These data were used for spatial registration and projection of channels onto a standardized cortical space. The session concluded with cap removal and debriefing.

### 2.4 fNIRS Data Acquisition

fNIRS data were recorded using two NIRSport2 systems (NIRx Medizintechnik GmbH, Germany): one 16×16 unit and one 8×8 unit, operating in tandem. Both devices are continuous-wave, LED-based systems that emit light at two wavelengths (760 and 850 nm). The systems recorded hemodynamic signals at a sampling rate of 5.1 Hz. The optode configuration included 24 sources and 30 detectors, of which 8 were short-separation detectors included to measure superficial physiological signals and enable regression of extracerebral noise. The optodes were embedded in EEG-compatible 10/20 caps (sizes 54, 56, 58, and 60 cm), with cap size selected based on individual head circumference.

Source illumination power was automatically adjusted by the acquisition software based on real-time signal-to-noise ratio (SNR) estimates. If a channel’s SNR was below threshold, source intensity was increased to improve signal quality.

### 2.5 Montage Design and Individual Headshapes

**Fig. 2:**
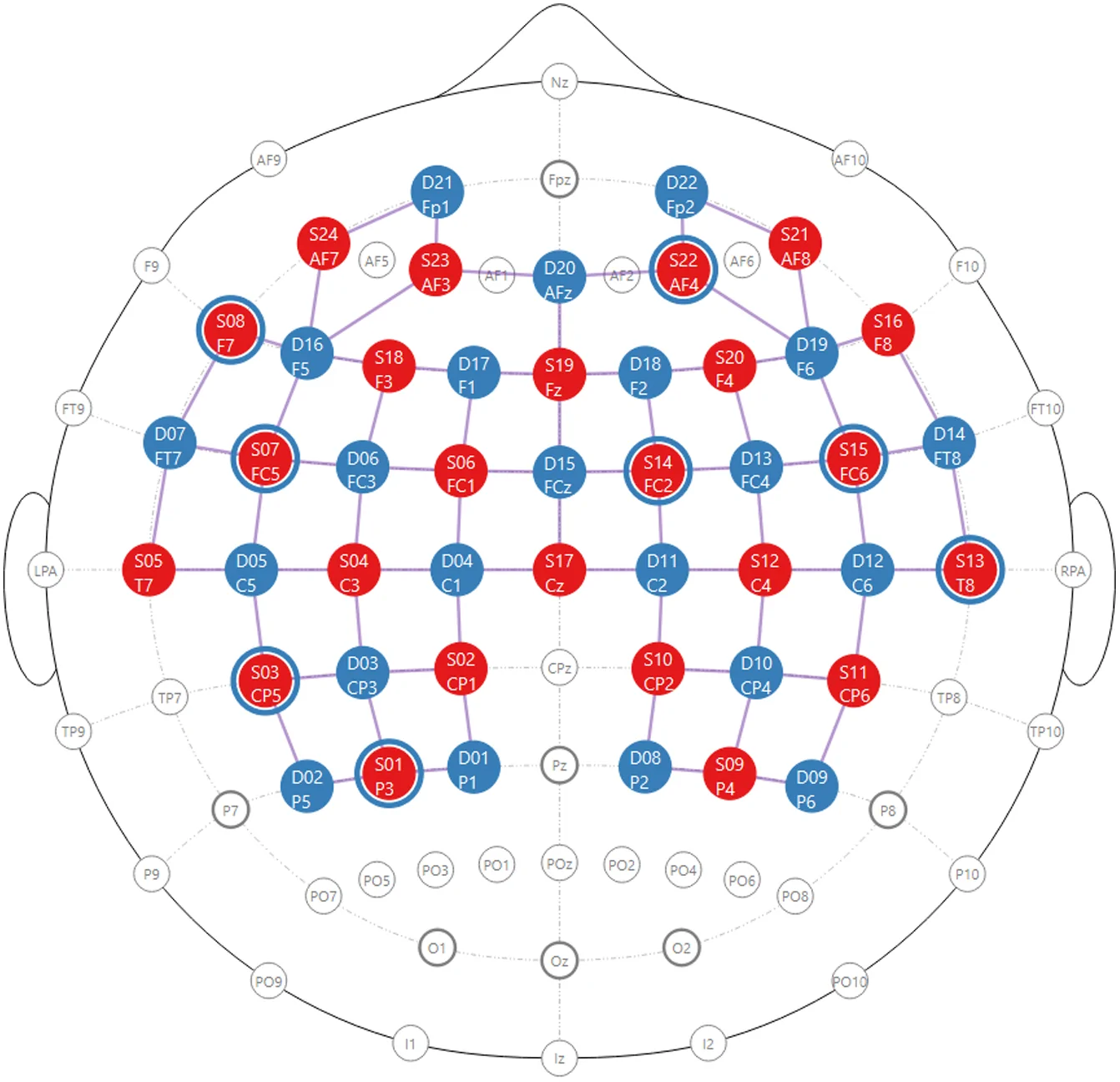
The standard fNIRS optode montage used in the current study. Red circles indicate source optodes, and blue circles indicate detector optodes. Blue outlines surrounding selected source optodes indicate the locations of short-separation channels used to measure superficial physiological signals. Lines connecting sources and detectors represent measurement channels (i.e., source–detector pairs). For the single-subject analysis, exact channel positions varied.

The montage was custom-designed to achieve broad cortical coverage, excluding only the occipital region, which was not the focus of this study. As depicted in Fig. 2, the montage included 73 long-separation channels spanning bilateral frontal, temporal, and parietal regions, along with eight short-separation channels used to measure superficial physiological signals. This design was chosen to target regions commonly involved in auditory and speech processing, including the superior temporal gyrus, inferior frontal gyrus, PMC, and somatosensory areas.

Short-separation channels were included to capture superficial physiological signals arising from scalp and extracerebral tissue and were defined by source–detector distances of ∼8  mm. Long-separation channels had a mean distance of 35 mm to allow measurement of cortical hemodynamic responses. Actual source–detector distances varied across participants due to anatomical differences in head shape and cap size. Therefore, we derived individualized source-detector distances from 3D head shape scans.

### 2.6 Headshape Digitization

To localize fNIRS optode positions relative to individual brain anatomy, we obtained 3D head scans from each participant using the Scaniverse mobile application on a smartphone, which outputs mesh files in .obj format. Mesh files represent the 3D surface geometry of the scanned head as a set of vertices and polygonal faces. Unlike approaches that rely on electromagnetic tracking devices (e.g., Polhemus wands) or individual structural MRI scans to register optode positions to cortical surfaces,^61^ this method enables spatial registration using a mobile 3D scanning application without requiring specialized hardware. After completion of the tasks, participants remained seated while the springs and optodes were removed, leaving the cap with channel labels in place. The experimenter then slowly moved around the participant with the device, capturing the head from multiple angles and distances to ensure full surface coverage and accurate reconstruction of the cap and landmark positions.

The resulting head models were imported into MATLAB and processed using the FieldTrip toolbox.^62^ First, each scan was aligned to a standard coordinate system using ft_meshrealign, based on manual rotation to match orientation conventions (positive z-axis upward). Next, cranial landmarks, including the nasion, left and right preauricular points, and Cz, were manually annotated using ft_electrodeplacement. Optode positions were then identified manually by referencing the 2D layout of our montage. The identified optode locations were saved to a digitization file (digpts.txt) unique to each participant. These files were then imported into NIRSite, where custom montage files (.mat) were created and later attached to each participant’s SNIRF file, which stores the fNIRS recordings and associated metadata. The files were then loaded into Satori, an fNIRS analysis software developed by NIRx and Brain Innovation (Satori v2.2.4, Brain Innovation, Maastricht, Netherlands (NIRx Medical Technologies^63^)).

For the group-level analysis, the individual digitized optode coordinates were used to compute a group-averaged montage. A custom MATLAB script calculated the mean spatial position of each optode across participants after excluding outlier positions that exceeded a predefined standard deviation threshold. The resulting averaged coordinate file (group_digpts.txt) was imported into NIRSite and saved as a standard probe configuration, which was then applied uniformly to all participants for group-level modeling. Across participants, the mean spatial variability of optode positions relative to the group-averaged coordinates was 4.16 mm, with a maximum deviation of 7.37 mm, reflecting the average and maximum distances between individual optode locations and the common template.

### 2.7 fNIRS Data Preprocessing

Data preprocessing was performed similarly for the speak and listen datasets. First, individual SNIRF files were loaded into Satori, along with the corresponding individual montages. Those datasets were imported into the Satori workflow manager for batch processing. Raw intensity signals were first converted to optical density. Each dataset was trimmed to remove signal outside of the task period, keeping 2 s before the first event and 2 s after the last event to avoid movement artifacts or post-task speech (e.g., letting the researcher know they are done with the task). Preprocessing steps included channel rejection based on scalp coupling index (SCI; threshold=0.75),^64^ spike correction (10 iterations, lag=5, threshold=3.5, influence=0.5), and temporal derivative distribution repair (TDDR) with high-frequency restoration.^65^ Short-channel regression was applied using a GLM-based approach with the most correlated short-channel regressor and with specific chromophores (HbO and HbR).^48^^,^^49^ Temporal filtering included high-pass Butterworth filtering at 0.01 Hz and low-pass Gaussian smoothing (0.4 Hz). Channel masks were applied to include only channels relevant to the listen and speak tasks, respectively, based on their estimated anatomical locations in MNI space and the established literature on speech and auditory processing networks. Because fNIRS channels have overlapping sensitivity profiles and limited spatial resolution,^66^^,^^67^ some channels were associated with multiple neighboring anatomical regions. Accordingly, the masks were intended to approximate broader cortical regions and functional networks rather than provide precise anatomical locations. Signals were converted to concentration changes in HbO and HbR using the modified Beer–Lambert law and z-transformed.

### 2.8 Channel Masks

To evaluate activation in expected cortical areas, task-specific channel masks were created for the listen and speak datasets. Channels were selected based on their anatomical correspondence to key regions in the speech and auditory network, including bilateral primary auditory cortex, superior temporal gyrus, inferior frontal gyrus (Broca’s area), premotor and motor cortex, SMA, and dorsolateral prefrontal cortex (dlPFC). These anatomical labels and MNI coordinates were estimated in Satori using standard optode locations registered to MNI space and referenced to the ICBM152 adult head atlas.^68^

**Fig. 3:**
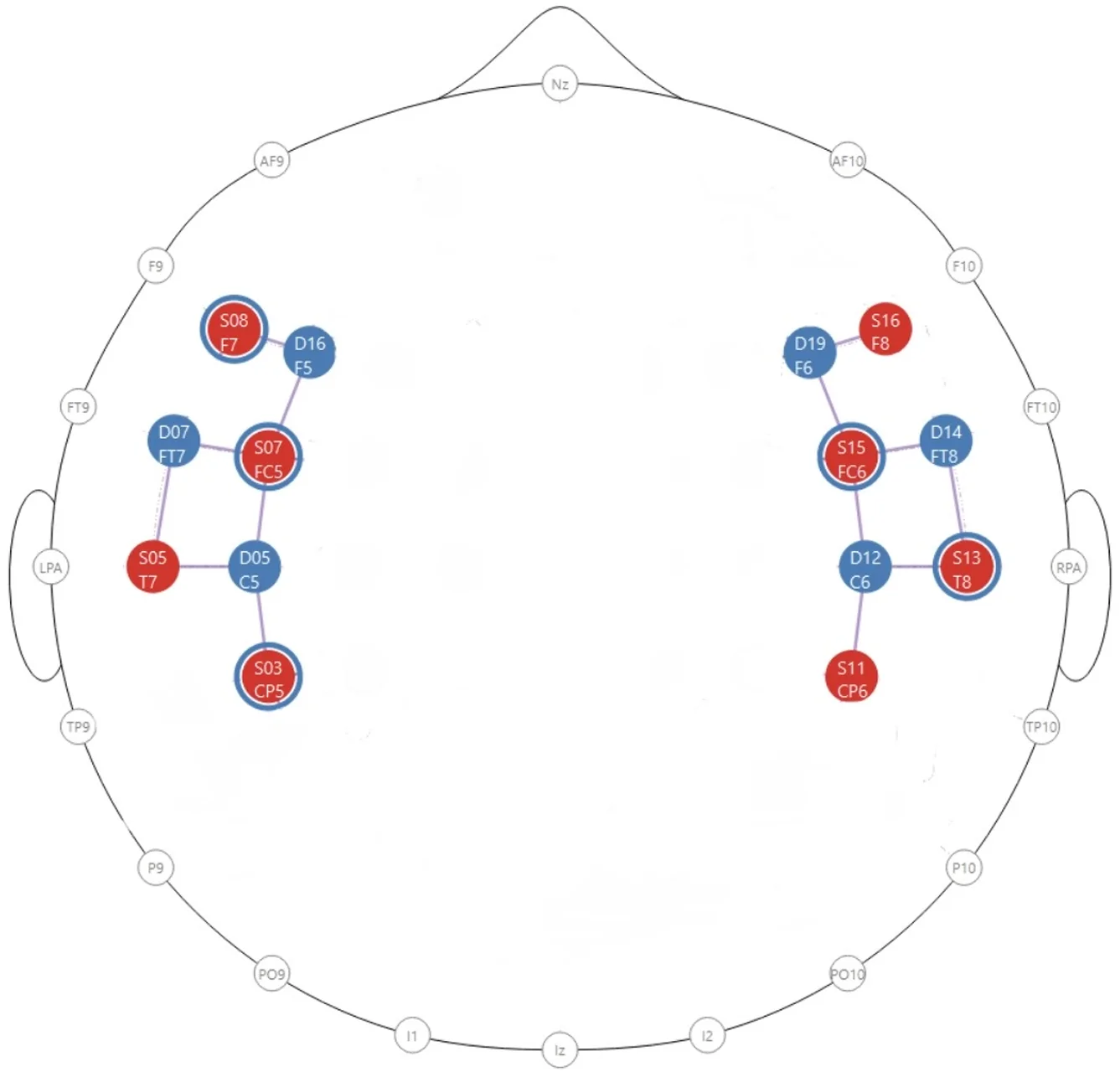
Channel mask for the listen task montage.

**Fig. 4:**
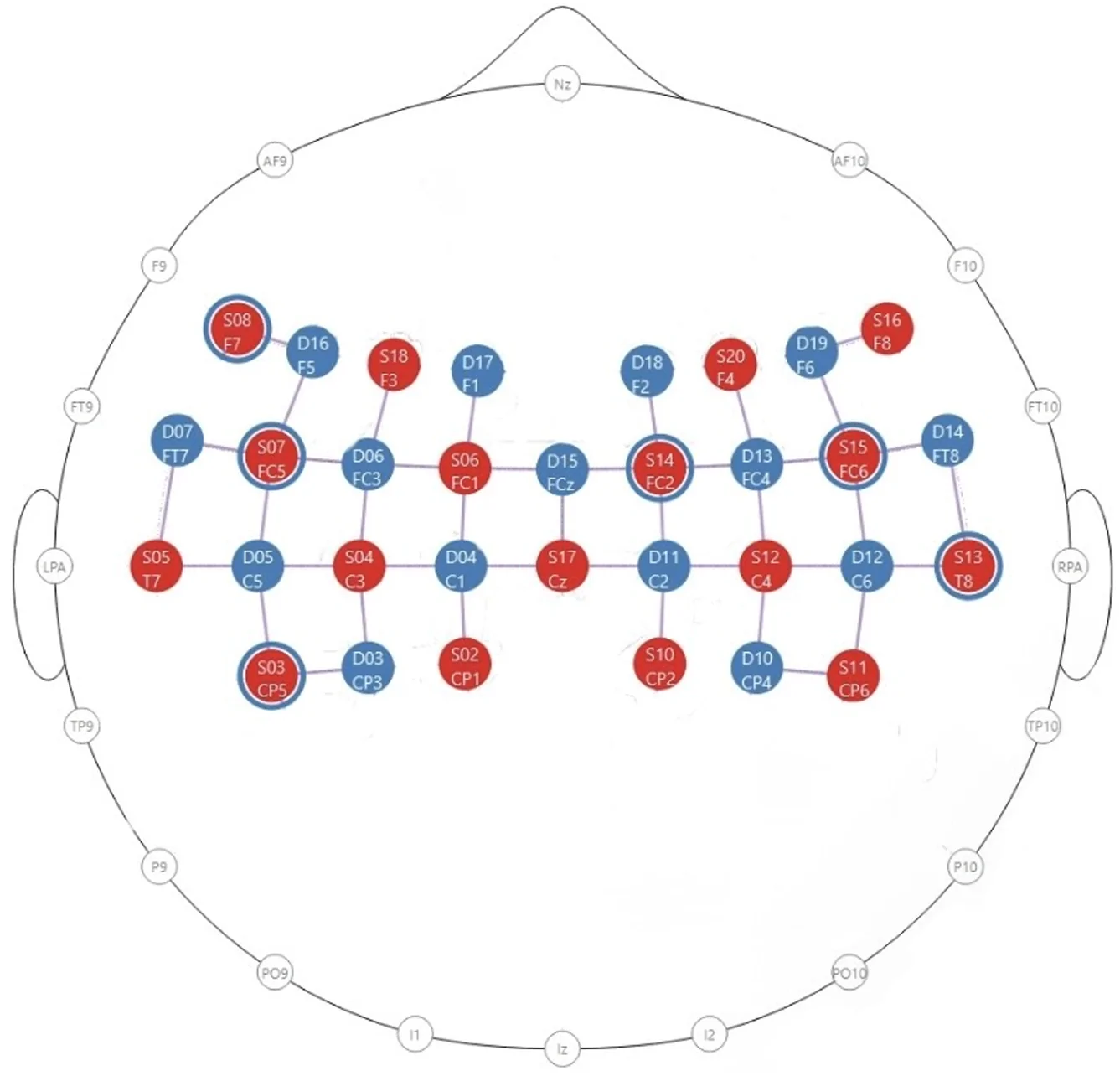
Channel mask for the speak task montage.

As presented in Tables 1 and 2 and Figs. 3 and 4, a total of 10 channels were included in the listen mask and 41 channels in the speak mask, reflecting broader expected engagement during speech production. A complete list of included channels, anatomical regions, MNI coordinates, and inclusion per task is provided in the Supplementary Materials (Table S2 in the Supplementary Material).

**Table 1:** Channel definitions for the listen task montage.

Channel	Source-detector pair	Cortical region
1	S3-D5	Left-Primary Auditory Cortex, Left-PMC and SMA, Left-Supramarginal Gyrus, Left-Superior Temporal Gyrus, Left- Primary Sensory Cortex
2	S5-D5	Left-Medial Temporal Gyrus, Left-Primary Auditory Cortex
3	S5-D7	Left-Medial Temporal Gyrus, Left-Superior Temporal Pole, Left-Temporal Pole
4	S7-D5	Left-PMC and SMA, Left-Broca’s Area
5	S7-D7	Left-Broca’s Area, Left-Insula, Left-Pars Orbitalis, Left-Temporal Pole
6	S7-D16	Left-Broca’s Area, Left-dlPFC
7	S8-D16	Left-Broca’s Area, Left-dlPFC, Left-Pars Orbitalis
8	S11-D12	Right-Primary Auditory Cortex, Right-Superior Temporal Gyrus, Right-Supramarginal Gyrus
9	S13-D12	Right-Primary Auditory Cortex, Right-Superior Temporal Gyrus, Right-Supramarginal Gyrus
10	S13-D14	Right-Medial Temporal Gyrus, Right-Superior Temporal Gyrus, Right-Temporal Pole
11	S15-D12	Right-Primary Motor Cortex, Right-Primary Sensory Cortex, Right Broca’s Area
12	S15-D14	Right Broca’s Area, Right-Insula, Right-Temporal Pole, Right-Superior Temporal Gyrus
13	S15-D19	Right Broca’s Area, Right-dlPFC
14	S16-D19	Right-Pars Orbitalis, Right-Broca’s Area

Source–detector pairs correspond to the optode geometry of the listen task probe. Cortical regions reflect template-based anatomical labels assigned to each channel and indicate the primary regions underlying the corresponding source–detector pair. Region labels are not mutually exclusive and reflect spatial overlap across cortical areas.

**Table 2:** Channel definitions for the speak task montage.

Channel	Source-detector pair	Cortical region
1	S2-D4	Left-PMC and SMA, Left-Primary Motor Cortex, Left-Primary Sensory Cortex, Left-Sensory Association Cortex
2	S3-D3	Left-Supramarginal Gyrus, Left-Angular Gyrus
3	S3-D5	Left-Primary Auditory Cortex, Left-PMC and SMA, Left-Supramarginal Gyrus, Left-Superior Temporal Gyrus, Left- Primary Sensory Cortex
4	S4-D3	Left-Primary Sensory Cortex
5	S4-D4	Left-PMC and SMA, Left-Primary Sensory Cortex
6	S4-D5	Left-Primary Sensory Cortex, Left-Primary Motor Cortex
7	S4-D6	Left-PMC and SMA, Left-Primary Sensory Cortex, Left-Frontal Eye Fields
8	S5-D5	Left-Medial Temporal Gyrus, Left-Primary Auditory Cortex
9	S5-D7	Left-Medial Temporal Gyrus, Left-Superior Temporal Pole, Left-Temporal Pole
10	S6-D4	Left-PMC and SMA
11	S6-D6	Left-Frontal Eye Fields, Left-PMC and SMA
12	S6-D15	Left-Frontal Eye Fields, Left-PMC and SMA
13	S6-D17	Left-Frontal Eye Fields, Left-PMC and SMA
14	S7-D5	Left-PMC and SMA, Left-Broca’s Area
15	S7-D6	Left-PMC and SMA, Left-Broca’s Area, Left-Frontal Eye Fields, Left-dlPFC
16	S7-D7	Left-Broca’s Area, Left-Insula, Left-Pars Orbitalis, Left-Temporal Pole
17	S7-D16	Left-Broca’s Area, Left-dlPFC
18	S8-D16	Left-Broca’s Area, Left-dlPFC, Left-Pars Orbitalis
19	S10-D11	Right-Primary Sensory Cortex, Right-Primary Motor Cortex and SMA
20	S11-D10	Right-Angular Gyrus, Right-Supramarginal Gyrus
21	S11-D12	Right-Primary Auditory Cortex, Right-Superior Temporal Gyrus, Right-Supramarginal Gyrus
22	S12-D10	Right-Primary Sensory Cortex, Right- Supramarginal Gyrus, Right-Primary Sensory Cortex
23	S12-D11	Right-PMC and SMA, Right-Primary Sensory Cortex
24	S12-D12	Right-Primary Sensory Cortex, Right-Primary Motor Cortex and SMA
25	S12-D13	Right-PMC and SMA, Right-Frontal Eye Fields, Right-Primary Motor Cortex, Right-Broca’s Area
26	S13-D12	Right-Primary Auditory Cortex, Right-Superior Temporal Gyrus, Right-Supramarginal Gyrus
27	S13-D14	Right-Medial Temporal Gyrus, Right-Superior Temporal Gyrus, Right-Temporal Pole
28	S14-D11	Right-PMC and SMA
29	S14-D13	Right-Frontal Eye Fields, Right-PMC and SMA
30	S14-D15	Right-Frontal Eye Fields, Right-PMC and SMA
31	S14-D18	Right-Frontal Eye Fields, Right-PMC and SMA
32	S15-D12	Right-Primary Motor Cortex, Right-Primary Sensory Cortex
33	S15-D13	Right-Broca’s Area, Right-Frontal Eye-Fields
34	S15-D14	Right Broca’s Area, Right-Insula, Right-Temporal Pole, Right-Superior Temporal Gyrus
35	S15-D19	Right, Broca’s Area, Right-dlPFC
36	S16-D19	Right-Pars Orbitalis, Right-Broca’s Area
37	S17-D4	Left-PMC and SMA
38	S17-D11	Right-PMC and SMA
39	S17-D15	Left/Right-PMC and SMA
40	S18-D6	Left-Frontal Eye Fields, Left-dlPFC
41	S20-D13	Right-Frontal Eye Fields, Right-PMC and SMA, Right-dlPFC

Source–detector pairs correspond to the optode geometry of the speak task probe. Cortical regions reflect template-based anatomical labels assigned to each channel and indicate the primary regions underlying the corresponding source–detector pair. Region labels are not mutually exclusive and reflect spatial overlap across cortical areas.

### 2.9 General Linear Model

GLM modeling was conducted separately for the listen and speak datasets. Listen events were modeled using a 2-s boxcar function, reflecting the passive nature of the task and approximating the average duration of the auditory stimuli used in the paradigm (M=1.55  s, SD=0.22). Speak events were modeled using a 3-s boxcar function to capture the expected duration of visual word processing, speech initiation, and overt articulation. In both datasets, the inter-trial interval served as the baseline condition. Event durations reflected the estimated period of task engagement, whereas the slower hemodynamic response was modeled through convolution of the boxcar functions with the canonical hemodynamic response function (HRF) within the GLM framework.^49^ Channels were classified as showing task-related activation only if the GLM contrast comparing task-related activity to baseline reached statistical significance and showed physiologically plausible directionality (positive t for HbO, negative t for HbR).^37^^,^^67^^,^^36^^,^^69^ False discovery rate (FDR) correction was applied separately to HbO and HbR contrasts using the Benjamini–Hochberg procedure,^70^ which ranks p-values and determines a chromophore-specific significance threshold to control the expected proportion of false discoveries.

To examine the relationship between chromophore responses, Spearman rank correlations were computed between HbO and HbR t-values across channels that showed significant task-related activation. This analysis assessed whether channels with stronger HbO increases also showed corresponding HbR decreases, consistent with the expected pattern of neurovascular coupling.

### 2.10 Group-Level Analysis

Although the primary aim of this study was to evaluate data quality and activation patterns at the single-subject level, we also conducted group-level analyses. Group-level models are standard in the fNIRS literature, and including them allows our results to be compared directly with prior studies and with common analytic practices.

Individual headshape information could not be incorporated into the group-level GLM because the group model requires an identical channel layout across participants. Using subject-specific headshapes would alter channel positions and effectively result in slightly different montages across participants. Therefore, the group-level analysis was conducted using a common template montage, defined as the group-averaged optode configuration, and individual variations in optode placement were not modeled at this stage.

### 2.11 Participant and Channel Inclusion

The single-subject analyses were designed to characterize data quality and activation patterns within individuals. For this reason, channels were not removed at the single-subject level. All channels were retained so that we could evaluate how often each channel produced usable signals, how individual data quality varied across the montage, and how these factors influenced single-subject activation estimates.

By contrast, the group-level GLM requires an identical channel layout across participants. Channels that are removed due to poor data quality in some participants would lead to unequal data dimensions and invalidate the group model. Therefore, channel and participant exclusion were applied only for the group-level analysis.

Channel-level quality was assessed using the SCI. Channels were considered included if SCI≥0.75. For the group analysis, a channel was retained only if at least half of the participants (≥14 of 27) showed SCI≥0.75 for that channel. Participants were included if at least half of their channels exceeded the same threshold (≥5 of 10 channels for listen; ≥21 of 41 channels for speak).

After applying the channel and participant exclusion thresholds, all remaining channels were kept in the group model. Remaining channels with SCI<0.75 in some participants were not removed, ensuring that every participant contributed data with the same channel layout.

## 3 Results

### 3.1 Single-Subject Analysis

All participants were included in the single-subject analysis. Activation estimates were computed separately for the listen and speak datasets and separately for HbO and HbR. A channel was classified as significant when it showed a statistically significant effect after applying the individual FDR corrected threshold, and the effect was in the expected direction (HbO increase, HbR decrease). Detailed channel-level significance tables for each participant are provided in the Supplementary Materials [Tables S5, S6, S7(a)–S7(d), and S8(a)–S8(d) in the Supplementary Material], as well as exact FDR thresholds (Tables S3 and S4 in the Supplementary Material), and tables reporting exact t- and p-values (Tables S9–S61 in the Supplementary Material).

### 3.2 Listen Task

#### 3.2.1 Participant-level

The number of channels retained within the mask in each participant after SCI channel rejection ranged from 2 to 14 (M=10.93, SD=3.86). There were no participants with no retained channels (see Table 3).

**Table 3:** Participant-level activation summary (listen task).

Participant	Rejected channels	Retained channels	Significant HbO	Significant HbR
pp1	0	14	1	3
pp2	0	14	1	5
pp3	2	12	1	0
pp4	0	14	1	1
pp5	2	12	2	4
pp6	9	5	1	0
pp7	11	3	1	1
pp8	3	11	8	5
pp9	1	13	4	2
pp10	0	14	0	9
pp11	12	2	0	1
pp12	1	13	1	1
pp13	1	13	0	1
pp14	0	14	7	1
pp15	4	10	2	2
pp16	11	3	0	1
pp17	5	9	0	2
pp18	2	12	0	1
pp19	0	14	2	3
pp20	0	14	3	2
pp21	9	5	1	0
pp22	3	11	1	2
pp23	4	10	5	1
pp24	1	13	0	0
pp25	0	14	4	2
pp26	2	12	6	4
pp27	0	14	2	2

Each value represents the number of channels rejected or retained after SCI thresholding or showed significant activation within the predefined listen mask for each participant.

For each participant, separate FDR thresholds were computed for HbO and HbR contrasts, and these values were used to determine statistical significance. Twenty-one participants (78%) showed at least one significant HbO channel within the predefined region mask. The number of channels significant in each participant ranged from 0 to 8 (M=2.00, SD=2.25). All 27 participants (100%) showed at least one significant HbR channel within the predefined region mask. The number of channels significant in each participant ranged from 2 to 14 (M=8.93, SD=3.93).

To assess the extent to which HbO and HbR activations are coupled across participants, we calculated a Spearman correlation between the number of significant channels for each chromophore. A positive association was observed between the number of significant HbO and HbR channels across participants, although this relationship did not reach statistical significance, $r_{s}=.34$, p=.080.

#### 3.2.2 Channel-level

The number of participants in which each channel was retained ranged from 19 to 24 (M=21.07, SD=1.49), as detailed in Table 4. The number of participants showing significant HbO activation per channel ranged from 0 to 9 (M=3.86, SD=2.90). The number of participants showing significant HbR activation per channel ranged from 0 to 15 (M=4.00, SD=4.39). For example, channel 10 in the right hemisphere—covering the right middle temporal gyrus, the superior temporal gyrus, and the temporal pole—was significant in 9 of 27 participants (33%) in HbO and 15 participants (56%) in HbR. By contrast, channel 5 in the left hemisphere—covering Broca’s area, insula, pars orbitalis, and the temporal pole—was not significant in any participant in HbO and was significant in only one participant in HbR.

**Table 4:** Channel-level activation summary (listen task).

Channel	Rejected	Retained	Significant HbO	Significant HbR
1	6	21	7	1
2	6	21	5	7
3	7	20	4	2
4	7	20	2	1
5	8	19	0	1
6	6	21	2	2
7	6	21	0	1
8	3	24	6	5
9	6	21	8	11
10	3	24	9	15
11	6	21	1	0
12	5	22	2	4
13	8	19	3	1
14	6	21	5	5

Each value represents the number of participants in which the corresponding channel was retained after SCI thresholding or showed significant activation within the predefined listen mask. Channels are ordered by their numeric identifiers.

**Fig. 5:**
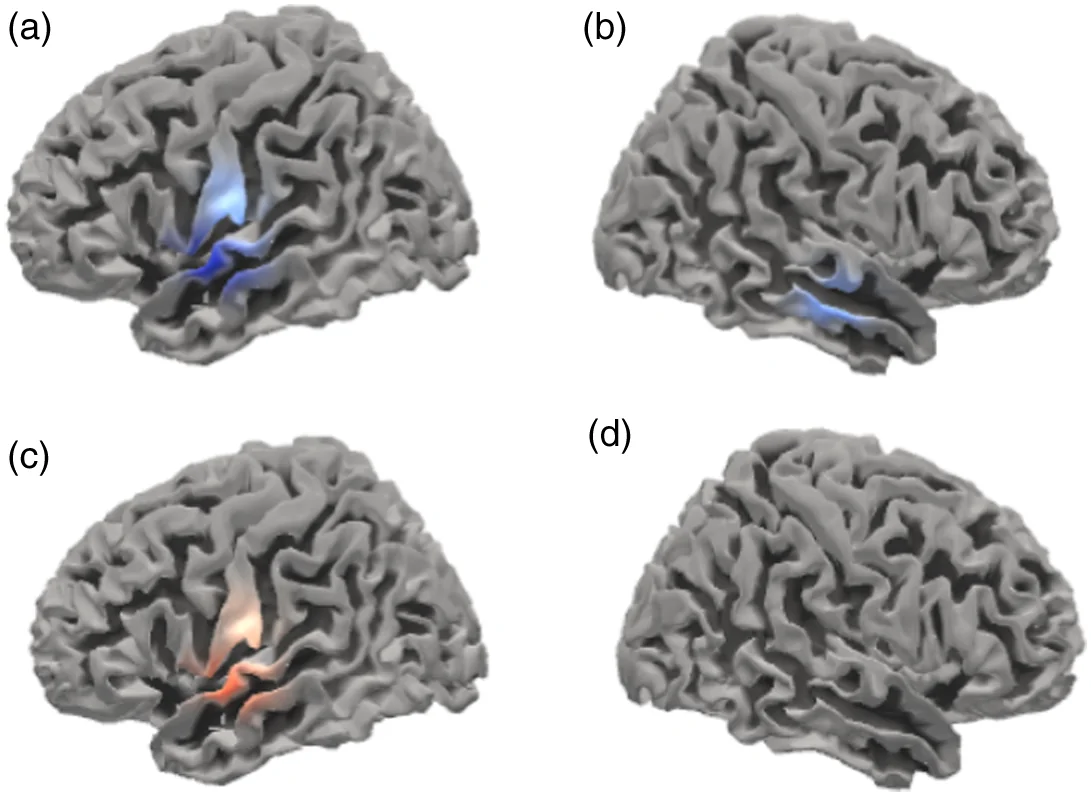
Example of physiologically plausible single-subject activation pattern during the listen task. (a) HbR activation, left hemisphere. (b) HbR activation, right hemisphere. (c) HbO activation, left hemisphere. (d) HbO activation, right hemisphere. Color intensity reflects the magnitude of the GLM contrast (listen > baseline), with warmer colors (red) indicating stronger positive activation, cooler colors (blue) indicating stronger negative activation, and colors closer to white indicating values nearer to zero. The pattern shows spatially coherent task-related activation with the expected inverse HbO–HbR relationship.

We also assessed how consistently the same channels yielded significant HbO and HbR effects across participants. The analysis revealed a strong positive association, ρ=.73, p=.003, indicating that channels showing significant HbO activation in a larger number of participants also tended to show significant HbR activation in a larger number of participants. A representative example of a single-subject activation pattern illustrating an expected response is shown in Fig. 5.

### 3.3 Speak Task

#### 3.3.1 Participant-level

The number of channels retained in each participant ranged from 4 to 41 (M=25.18, SD=10.36). There were no participants with no retained channels (see Table 5).

**Table 5:** Participant-level activation summary (speak task).

Participant	Rejected channels	Retained channels	Significant HbO	Significant HbR
pp1	8	33	15	8
pp2	10	31	0	2
pp3	7	34	22	24
pp4	20	21	2	1
pp5	0	41	9	18
pp6	30	11	0	1
pp7	37	4	1	1
pp8	7	34	28	14
pp9	26	15	1	0
pp10	18	23	7	10
pp11	32	9	0	5
pp12	20	21	2	4
pp13	23	18	4	3
pp14	8	33	14	14
pp15	20	21	4	7
pp16	35	6	5	0
pp17	22	19	3	3
pp18	16	25	11	9
pp19	13	28	0	1
pp20	11	30	4	16
pp21	0	41	0	3
pp22	3	38	21	8
pp23	12	29	25	11
pp24	19	22	2	13
pp25	1	40	24	17
pp26	13	28	21	10
pp27	16	25	9	15

Each value represents the number of channels rejected or retained after SCI thresholding or showed significant activation within the predefined speak mask for each participant.

Twenty-two participants (81%) showed at least one significant HbO channel within the predefined mask. The number of channels significant in each participant ranged from 0 to 28 (M=8.67, SD=9.14. Twenty-five participants (93%) showed at least one significant HbR channel within the predefined region mask. The number of channels significant in each participant ranged from 0 to 24 (M=8.07, SD=6.58).

To examine the correspondence between HbO and HbR responses, we computed the correlation coefficient between the number of significant channels for each chromophore across participants. This analysis showed a strong positive relationship, $r_{s}=.70$, p<.001, indicating that participants who exhibited greater HbO activation also tended to show HbR effects in the expected direction.

#### 3.3.2 Channel-level

As shown in Table 6, the number of participants in which each channel was retained ranged from 7 to 26 (M=16.58, SD=4.75), and the number of participants showing significant HbO activation per channel ranged from 2 to 12 (M=5.71, SD=2.32). The number of participants showing significant HbR activation per channel ranged from 1 to 12 (M=5.32, SD=3.13). For example, channel 6 in the left hemisphere—covering the primary sensory and motor cortex—was significant in 12 of 27 participants (44%) in HbO and 10 participants (37.04%) in HbR. By contrast, channel 28 in the right hemisphere—covering the pre-motor and SMA —was only significant in two participants (7%) in HbO and one participant (4%) in HbR. There were no channels that were not retained or not significant in at least one participant.

**Table 6:** Channel-level activation summary (speak task).

Channel	Rejected	Retained	Significant HbO	Significant HbR
1	19	8	3	3
2	15	12	4	2
3	7	20	6	7
4	10	17	6	2
5	14	13	4	3
6	5	22	12	10
7	10	17	7	8
8	5	22	8	8
9	5	22	3	4
10	17	10	4	4
11	14	13	3	4
12	11	16	8	5
13	14	13	3	2
14	6	21	10	10
15	13	14	6	6
16	6	21	6	8
17	7	20	7	5
18	9	18	5	1
19	17	10	4	2
20	6	21	2	3
21	3	24	8	12
22	13	14	9	2
23	17	10	5	4
24	7	20	9	9
25	9	18	11	6
26	6	21	6	11
27	1	26	4	12
28	20	7	2	1
29	15	12	6	6
30	14	13	6	6
31	15	12	5	2
32	8	19	3	7
33	9	18	7	10
34	4	23	5	6
35	7	20	4	5
36	10	17	5	2
37	15	12	5	4
38	12	15	6	5
39	16	11	5	4
40	9	18	6	1
41	7	20	6	6

Each value represents the number of participants in which the corresponding channel was retained after SCI thresholding or showed significant activation within the predefined listen mask. Channels are ordered by their numeric identifiers.

**Fig. 6:**
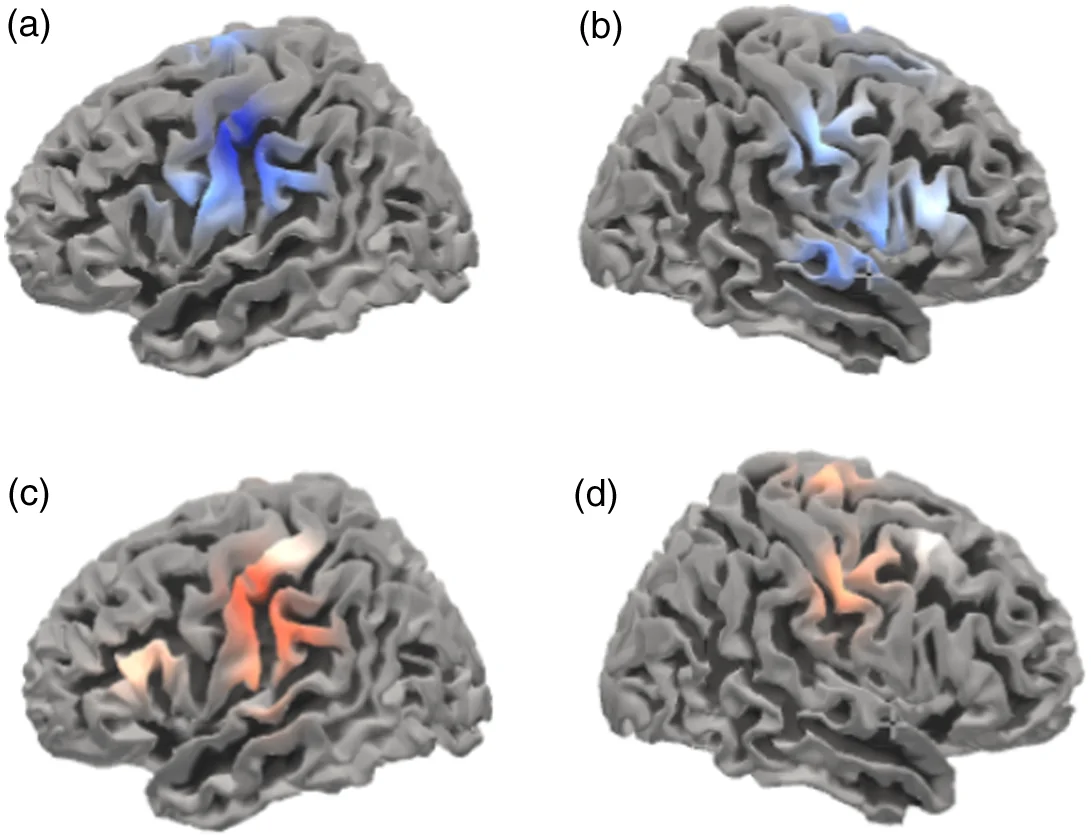
Example of physiologically plausible single-subject activation pattern during the speak task. (a) HbR activation, left hemisphere. (b) HbR activation, right hemisphere. (c) HbO activation, left hemisphere. (d) HbO activation, right hemisphere. Color intensity reflects the magnitude of the GLM contrast (speak > baseline), with warmer colors (red) indicating stronger positive activation, cooler colors (blue) indicating stronger negative activation, and colors closer to white indicating values nearer to zero. The activation pattern shows spatially coherent task-related responses with the expected inverse relationship between HbO increases and HbR decreases.

**Fig. 7:**
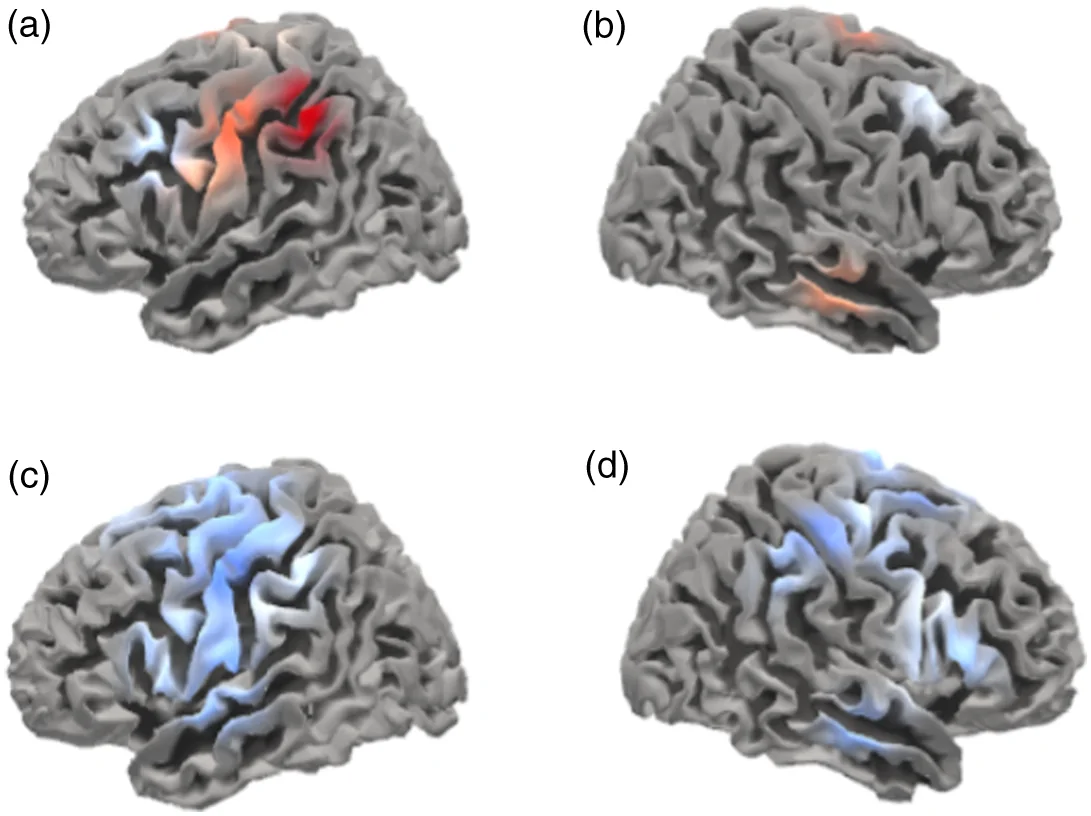
Example of non-interpretable single-subject activation during the speak task. (a) HbR activation, left hemisphere. (b) HbR activation, right hemisphere. (c) HbO activation, left hemisphere. (d) HbO activation, right hemisphere. Color intensity reflects the magnitude of the GLM contrast (speak > baseline), with warmer colors (red) indicating stronger positive activation, cooler colors (blue) indicating stronger negative activation, and colors closer to white indicating values nearer to zero. Although widespread activation is present, the directionality of HbO and HbR responses is inconsistent with canonical hemodynamic patterns, rendering the activation physiologically implausible.

We computed the correlation coefficient between the number of significant channels for each chromophore to examine the correspondence between channel-level significance frequencies for HbO and HbR across participants. The analysis revealed a moderate positive association, $r_{s}=.53$, p<.001. Representative examples of single-subject activation patterns during the Speak Task illustrating physiologically plausible and non-interpretable responses are shown in Figs. 6 and 7.

### 3.4 Group-Level Analyses

#### 3.4.1 Participant and channel inclusion

Four participants were excluded from the listen dataset because at least half of the channels within the predefined mask fell below the SCI threshold. Across participants, all channels met the group-level retention criterion and were included in the listen montage. Group-level GLM estimates were therefore computed across the full set of listen channels for the remaining participants.

Six participants were excluded from the speak dataset based on the predefined quality criteria. Fifteen channels did not meet the group-level retention threshold and were removed from the speak montage; the retained channel definitions are presented in Table 7. The remaining channels formed a consistent mask across participants for the group-level GLM analysis.

**Table 7:** Channel definitions for the speak task with channels included in group-level analysis.

Channel	Source-detector pair	Cortical region
1	S3-D5	Left-Primary Auditory Cortex, Left-PMC and SMA, Left-Supramarginal Gyrus, Left-Superior Temporal Gyrus, Left- Primary Sensory Cortex
2	S4-D3	Left-Primary Sensory Cortex
3	S4-D5	Left-Primary Sensory Cortex, Left-Primary Motor Cortex
4	S4-D6	Left-PMC and SMA, Left-Primary Sensory Cortex, Left-Frontal Eye Fields
5	S5-D5	Left-Medial Temporal Gyrus, Left-Primary Auditory Cortex
6	S5-D7	Left-Medial Temporal Gyrus, Left-Superior Temporal Pole, Left-Temporal Pole
7	S6-D15	Left-Frontal Eye Fields, Left-PMC and SMA
8	S7-D5	Left-PMC and SMA, Left-Broca’s Area
9	S7-D7	Left-Broca’s Area, Left-Insula, Left-Pars Orbitalis, Left-Temporal Pole
10	S7-D16	Left-Broca’s Area, Left-dlPFC
11	S8-D16	Left-Broca’s Area, Left-dlPFC, Left-Pars Orbitalis
12	S11-D10	Right-Angular Gyrus, Right-Supramarginal Gyrus
13	S11-D12	Right-Primary Auditory Cortex, Right-Superior Temporal Gyrus, Right-Supramarginal Gyrus
14	S12-D10	Right-Primary Sensory Cortex, Right- Supramarginal Gyrus, Right-Primary Sensory Cortex
15	S12-D12	Right-Primary Sensory Cortex, Right-Primary Motor Cortex and SMA
16	S12-D13	Right-PMC and SMA, Right-Frontal Eye Fields, Right-Primary Motor Cortex, Right-Broca’s Area
17	S13-D12	Right-Primary Auditory Cortex, Right-Superior Temporal Gyrus, Right-Supramarginal Gyrus
18	S13-D14	Right-Medial Temporal Gyrus, Right-Superior Temporal Gyrus, Right-Temporal Pole
19	S15-D12	Right-Primary Motor Cortex, Right-Primary Sensory Cortex
20	S15-D13	Right-Broca’s Area, Right-Frontal Eye-Fields
21	S15-D14	Right Broca’s Area, Right-Insula, Right-Temporal Pole, Right-Superior Temporal Gyrus
22	S15-D19	Right, Broca’s Area, Right-dlPFC
23	S16-D19	Right-Pars Orbitalis, Right-Broca’s Area
24	S17-D11	Right-PMC and SMA
25	S18-D6	Left-Frontal Eye Fields, Left-dlPFC
26	S20-D13	Right-Frontal Eye Fields, Right-PMC and SMA, Right-dlPFC

Source–detector pairs correspond to the optode geometry of the speak task probe for group-level analysis. Cortical regions reflect template-based anatomical labels assigned to each channel and indicate the primary regions underlying the corresponding source–detector pair. Region labels are not mutually exclusive and reflect spatial overlap across cortical areas.

#### 3.4.2 Listen task

The listen task elicited positive HbO t-values (t>0) across all channels at the group level (see Table 8). Six out of 14 channels (43%) showed significant HbO increases when applying the FDR-corrected threshold (p<.021).

**Table 8:** Group-level GLM results for the listen task.

Channel	Source-detector pair	HbO t	HbO p	HbO d	HbR t	HbR p	HbR d
1	S3-D5	2.24	0.036	0.47	−2.62	0.016*	−0.55
2	S5-D5	4.49	<.001*	0.94	−4.36	<.001*	−0.91
3	S5-D7	1.99	0.060	0.41	−2.48	0.021*	−0.52
4	S7-D5	2.49	0.021*	0.52	−1.80	0.085	−0.38
5	S7-D7	0.07	0.949	0.01	−0.51	0.617	−0.11
6	S7-D16	1.58	0.129	0.33	−1.47	0.155	−0.31
7	S8-D16	0.07	0.943	0.02	−0.74	0.467	−0.15
8	S11-D12	3.40	0.003*	0.71	−2.32	0.030	−0.48
9	S13-D12	3.27	0.004*	0.68	−4.02	0.001*	−0.84
10	S13-D14	3.71	0.001*	0.77	−5.80	<.001*	−1.21
11	S15-D12	2.25	0.034	0.47	−1.22	0.236	−0.25
12	S15-D14	0.84	0.408	0.18	−0.96	0.347	−0.20
13	S15-D19	3.82	0.001*	0.80	−3.57	0.002*	−0.74
14	S16-D19	2.25	0.035	0.47	−2.54	0.019*	−0.53

Table entries report group-level t, p, and Cohen’s d values for the contrast listen > baseline for HbO and HbR. t and d values are reported to two decimal places. Exact p values are reported when p≥.001; smaller values are reported as p<.001. Positive HbO and negative HbR responses represent the expected direction of task-related activation. Significant p–values are marked with an asterisk.

**Fig. 8:**
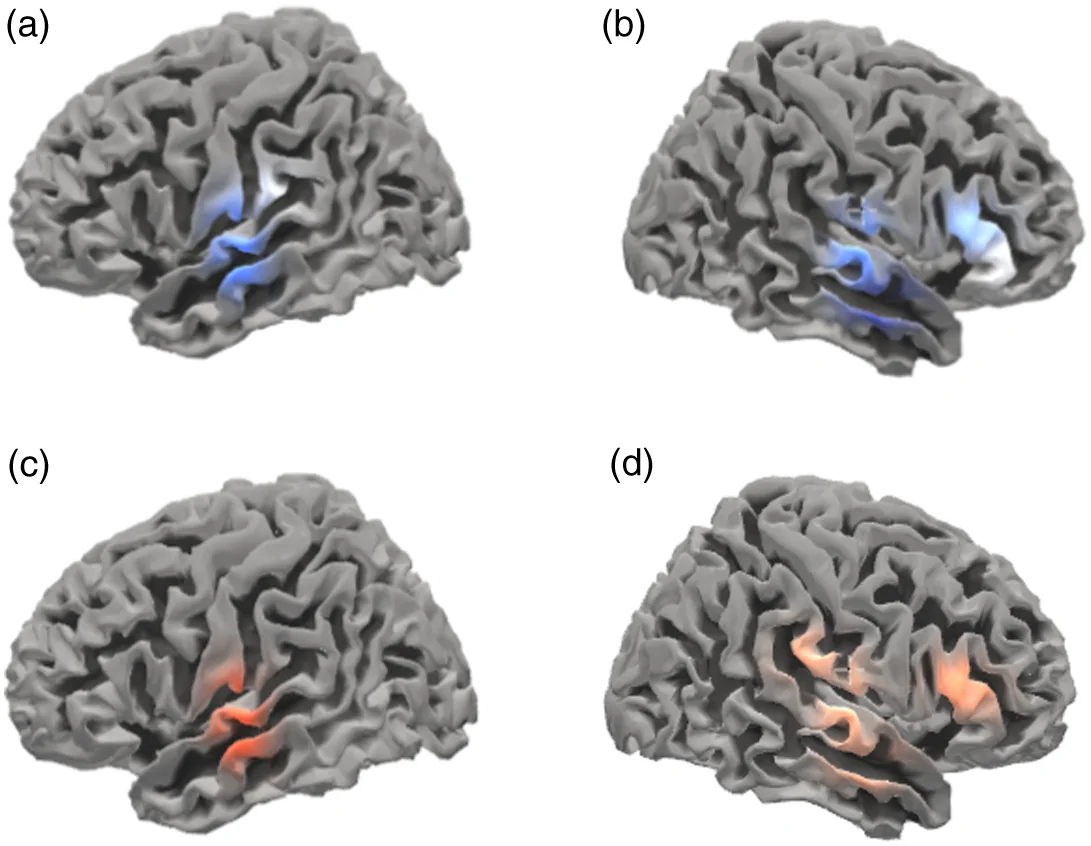
Group-level activation maps for the listen task. Panels show group-level GLM listen results projected onto an inflated cortical surface. (a) HbR activation, left hemisphere. (b) HbR activation, right hemisphere. (c) HbO activation, left hemisphere. (d) HbO activation, right hemisphere. Color intensity reflects the magnitude of the GLM contrast (listen > baseline), with warmer colors (red) indicating stronger positive activation, cooler colors (blue) indicating stronger negative activation, and colors closer to white indicating values nearer to zero. Only channels included in the group-level mask are shown.

HbR results showed consistent signal decreases (t<0) across all channels. Seven out of 14 channels (50%) exhibited significant HbR reductions when applying the FDR-corrected threshold (p<.021). The results are illustrated in Fig. 8. Group-level effect sizes are also reported in Table 8 and ranged from d=0.01 to 0.94 for HbO and from d=−0.11 to −1.21 for HbR.

#### 3.4.3 Speak task

At the group level, the speak task elicited widespread increases in HbO concentration (see Table 9). Twenty-five out of 26 channels (96%) showed significant HbO increases when applying the FDR-corrected threshold (p<.019), with only channel 21 failing to reach significance. All t-values were positive, which is consistent with expected task-related activation patterns.

**Table 9:** Group-level GLM results for the speak task.

Channel	Source-detector pair	HbO t	HbO p	HbO d	HbR t	HbR p	HbR d
1	S3-D5	2.56	<.001*	0.56	−5.64	<.001*	−1.23
2	S4-D3	2.94	<.001*	0.64	−0.75	0.464	−0.16
3	S4-D5	5.96	<.001*	1.30	−4.66	<.001*	−1.02
4	S4-D6	4.80	<.001*	1.05	−5.18	<.001	−1.13
5	S5-D5	2.95	0.008*	0.64	−3.66	0.002*	−0.80
6	S5-D7	0.15	<.001*	0.03	−1.46	<.001*	−0.32
7	S6-D15	2.02	<.001*	0.44	−3.00	<.001*	−0.65
8	S7-D5	4.72	<.001*	1.03	−3.38	0.003*	−0.74
9	S7-D7	2.85	<.001*	0.62	−2.28	<.001*	−0.50
10	S7-D16	3.79	<.001*	0.83	−1.89	<.001*	−0.41
11	S8-D16	1.32	<.001*	0.29	0.90	<.001*	0.20
12	S11-D10	3.13	<.001*	0.68	−0.29	<.001*	−0.06
13	S11-D12	3.81	<.001*	0.83	−3.68	<.001*	−0.80
14	S12-D10	4.37	<.001*	0.95	−2.14	<.001*	−0.47
15	S12-D12	6.15	<.001*	1.34	−4.98	<.001*	−1.09
16	S12-D13	5.72	<.001*	1.25	−4.48	<.001*	−0.98
17	S13-D12	1.41	<.001*	0.31	−5.09	<.001*	−1.11
18	S13-D14	0.05	<.001*	0.01	−4.17	<.001*	−0.91
19	S15-D12	2.93	<.001*	0.64	−4.06	<.001*	−0.89
20	S15-D13	4.64	<.001*	1.01	−4.36	<.001*	−0.95
21	S15-D14	0.89	0.383	0.19	−2.07	0.052	−0.45
22	S15-D19	1.41	<.001*	0.31	−3.21	<.001*	−0.70
23	S16-D19	1.74	<.001*	0.38	−1.17	<.001*	−0.26
24	S17-D11	2.09	<.001*	0.46	−1.10	<.001*	−0.24
25	S18-D6	1.09	<.001*	0.24	−0.89	<.001*	−0.20
26	S20-D13	2.92	<.001*	0.64	−2.36	<.001*	−0.51

Table entries report group-level t, p, and Cohen’s d values for the contrast speak > baseline for HbO and HbR. t and d values are reported to two decimal places. Exact p values are reported when p≥.001; smaller values are reported as p<.001. Positive HbO and negative HbR responses represent the expected direction of task-related activation. Significant p–values are marked with an asterisk.

**Fig. 9:**
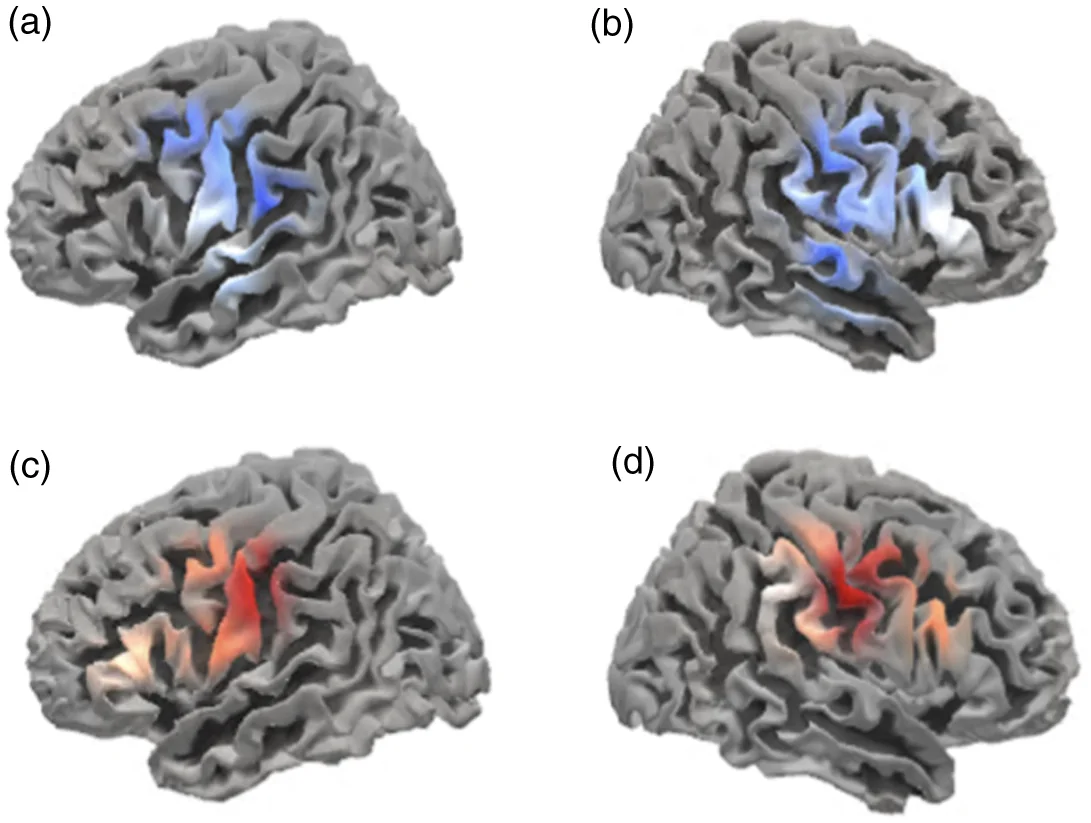
Group-level activation maps for the speak task. Panels show group-level GLM speak results projected onto an inflated cortical surface. (a) HbR activation, left hemisphere. (b) HbR activation, right hemisphere. (c) HbO activation, left hemisphere. (d) HbO activation, right hemisphere. Color intensity reflects the magnitude of the GLM contrast (speak > baseline), with warmer colors (red) indicating stronger positive activation, cooler colors (blue) indicating stronger negative activation, and colors closer to white indicating values nearer to zero. Only channels included in the group-level mask are shown.

HbR responses showed significant decreases in nearly all channels. Twenty-three out of 26 channels (88%) exhibited significant HbR reductions when applying the FDR-corrected threshold (p<.029), with only channels 2, 11, and 21 falling short of significance. All HbR t-values were negative except for channel 11, which showed a small positive effect. The results are illustrated in Fig. 9. Group-level effect sizes ranged from d=0.01 to 1.34 for HbO. HbR effect sizes were predominantly negative, ranging from d=−0.06 to −1.23. Channel 11 showed a small positive effect size (d=0.20), consistent with the positive HbR t value observed in that channel.

## 4 Discussion and Conclusion

This study explored the feasibility and reliability of single-subject-level analysis in fNIRS, using a simple listen and speak paradigm to engage well-established speech and auditory networks. Despite the widespread use of group-level analyses in neuroimaging, relatively little is known about the extent to which canonical activation patterns can be detected in individual fNIRS datasets. Our findings suggest that statistically interpretable activation patterns can be observed at the individual level in many participants, although substantial variability exists across participants, channels, and chromophores.

Across both tasks, the group-level results confirmed expected activation patterns. Listen elicited widespread HbR decreases and HbO increases across most regions, and speak produced even more robust and spatially widespread effects. These results provide important validation of the task itself and establish a useful benchmark for interpreting individual-level findings.

At the single-subject level, most participants showed evidence of task-related activation within predefined cortical masks. In the listen task, 78% of participants had at least one significant HbO channel and all participants showed at least one significant HbR channel. In the speak task, all participants showed at least some activation in the predefined masks for both chromophores. These findings suggest that task-related activation can often be detected at the individual level, although the number and spatial distribution of significant channels varied considerably across participants.

We further examined the coupling between HbO and HbR signals as an index of physiological reliability. At both the participant and channel levels, significant HbO and HbR effects were positively associated, indicating that individuals and regions showing stronger HbO responses also tended to show corresponding HbR decreases. Because canonical hemodynamic responses are characterized by simultaneous increases in HbO and decreases in HbR, this coupling provides an additional indicator of signal integrity beyond simple channel retention. Notably, only channels that met the FDR-corrected significance threshold and showed effects in the expected direction (positive for HbO, negative for HbR) were considered significant. Together, these findings suggest that variability in single-subject activation partly reflects differences in real physiological signal coherence rather than random noise.

Interestingly, HbR responses appeared more robust than HbO responses across participants in the listen task. Although HbO signals typically exhibit larger amplitude changes, they are also more susceptible to systemic physiological noise originating from scalp blood flow and other extracerebral sources.^14^^,^^39^ HbR changes, although smaller in magnitude, are often more spatially specific to cortical activation and more closely related to the BOLD signal measured with fMRI.^37^^,^^71^ As a result, HbR may sometimes provide a more stable indicator of task-related activation, particularly in single-subject analyses where physiological noise cannot be averaged out across participants. This interpretation is consistent with the higher single-subject detection rates observed for HbR relative to HbO in the listen task.

There was also considerable heterogeneity in significance patterns across participants. Importantly, the extent of activation varied considerably across participants, with some individuals showing only a few significant channels and others exhibiting widespread activation across the predefined masks. These differences may reflect physiological factors (e.g., hair thickness, skull geometry), cap placement, or individual variability in task engagement. Future studies should continue to explore these sources of variability and consider using within-subject designs to assess the reliability of single-subject results over time. For example, a repeated-measures design could help evaluate test–retest reliability of activation patterns in the same individual across sessions. It may also be valuable to investigate how task demands influence reliability at the single-subject level. The present findings suggest that the detectability of task-related activation can vary substantially across paradigms, participants, and chromophores. Increasing the number of trials per condition may also improve the detectability and stability of task-related activation at the single-subject level. Similarly, longer block designs may improve signal-to-noise ratio by allowing the hemodynamic response to accumulate over extended task periods, which may be advantageous when robust activation must be detected within a single individual.^39^ In addition, attentional engagement may vary across paradigms; future paradigms could incorporate mechanisms to monitor or increase attention during passive listening conditions, for example, by including occasional target-detection or working-memory probes. Future research could also explore whether more engaging or effortful paradigms elicit more reliable activation, even in low-burden protocols designed for clinical or applied use.

Several limitations of the present study should be considered. First, the listen and speak tasks were administered in a fixed order rather than randomized across participants, which may introduce potential order effects such as fatigue or practice-related changes in engagement. Second, the paradigm was intentionally designed to be brief and low burden, which limits the number of trials and may reduce statistical power for detecting activation at the single-subject level. In addition, the use of relatively simple, trisyllabic word stimuli may limit the extent to which the paradigm engages more complex phonological and articulatory processes. Future studies could explore the use of more linguistically complex stimuli, such as words with varying syllable structures or stress patterns, to increase task demands and potentially enhance the robustness of activation. Third, although we used physiologically informed criteria to evaluate signal reliability, fNIRS measurements remain sensitive to individual anatomical and biophysical factors such as hair thickness and scalp–cortex distance. In addition, the montage was originally designed for a related speech anticipation paradigm emphasizing frontal and parietal coverage, which limited the extent of posterior temporal sampling. Although the montage still included channels covering mid and posterior superior temporal regions associated with speech perception, broader coverage of posterior temporal cortex may have increased sensitivity to auditory and speech perception responses during the listen task Future studies should address these limitations by incorporating randomized task orders, increasing trial numbers, expanding posterior temporal coverage, and evaluating the test–retest reliability of single-subject activation patterns across sessions.

Overall, these findings support the potential value of single-subject fNIRS paradigms. Although group-level designs remain the gold standard in cognitive neuroscience, they are not always feasible or appropriate in applied contexts. Clinical assessments, individualized treatment monitoring, and brief screening tasks often require robust inferences to be made from a single person’s data. In such cases, group-level results cannot be generalized back to the individual, making single-subject analysis a potentially rewarding approach. Our results suggest that even brief, low-demand tasks can yield statistically interpretable activation patterns at the individual level in many participants, particularly when physiologically grounded quality metrics are incorporated. These findings highlight the potential for fNIRS to serve as a practical method for individualized neural assessment while also underscoring the substantial variability that remains at the single-subject level. Future research should continue to refine these approaches, evaluate their test–retest reliability in repeated-measures designs, and explore how task characteristics, such as cognitive demand, affect the robustness of single-subject results.

## Supplementary Material

10.1117/1.NPh.13.3.035009.s01

## Data Availability

The data and code that support the findings of this study are available from the corresponding author upon reasonable request.
