# Supplementary material for: Single-subject detection of speech network activation using functional near-infrared spectroscopy
Source: Neurophotonics. 2026 Sep 15;13(3):035009. doi: 10.1117/1.NPh.13.3.035009 (PMC13585373; doi:10.1117/1.NPh.13.3.035009)
Supplement: Supplementary file 1 [file NPh_013_035009_SD001.pdf]

Table S1

Word Stimuli Used in Listening and Speaking Tasks

**Words**
adventure
afternoon
agreement
animal
battery
camera
delicate
destruction
editor
elephant
explosion
formula
furniture
graduate
history
hospital
imagine
location
marketing
register
relative
reproduce
teenager
universe
unlucky

Table S2

Channel Definitions, MNI Coordinates, and Source–Detector Pairs

Channel	Cortical Region	MNI x-coordinates	MNI y-coordinates	MNI z-coordinates	Included in Listen	Included in Speak
S1-D1	Left Visual Motor Cortex	-31.06	-69.06	53.05	No	No
S1-D2	Left-Angular Gyrus	-52.11	-67.61	26.88	No	No
S1-D3	Left-Angular Gyrus, Left-Supramarginal Gyrus	-37.33	-58.17	46.32	No	No
S1-D23	Short Channel	-27.55	-65.32	44.61	No	No
S2-D1	Left-Visual Motor Cortex	-23.15	-60.18	69.49	No	No
S2-D3	Left-Primary Sensory Cortex	-37.07	-43.27	61.82	No	No
S2-D4	Left-PreMotor Area and SMA, Left-Primary Motor Cortex, Left-Primary Sensory Cortex, Left-Sensory Association Cortex	-29.15	-26.08	70.15	No	Yes
S3-D2	Left-Angular Gyrus	-56.8	-56.47	17.72	No	No
S3-D3	Left-Supramarginal Gyrus, Left-Angular Gyrus	-56.39	45.16	37.56	No	Yes
S3-D5	Left-Primary Auditory Cortex, Left-PreMotor and SMA, Left-Supramarginal Gyrus, Left-Superior Temporal Gyrus, Left- Primary Sensory Cortex	-64.37	-28.4	18.31	Yes	Yes
S3-D24	Short Channel	-55.15	40.57	16.54	No	No
S4-D3	Left-Primary Sensory Cortex	-54.77	-24.69	52.08	No	Yes
S4-D4	Left-PreMotor and SMA, Left-Primary Sensory Cortex	-29.37	-7.11	63.88	No	Yes
S4-D5	Left-Primary Sensory Cortex, Left-Primary Motor Cortex	-61.72	-12.42	31.74	No	Yes
S4-D6	Left-PreMotor and SMA, Left-Primary Sensory Cortex, Left-Frontal Eye Fields	-47.57	3.97	46.45	No	Yes
S5-D5	Left-Medial Temporal Gyrus, Left-Primary Auditory Cortex	-59.51	-12.17	2.45	Yes	Yes
S5-D7	Left-Medial Temporal Gyrus, Left-Superior Temporal Pole, Left-Temporal Pole	-60.62	-5.62	-22.11	Yes	Yes
S6-D4	Left-PreMotor and SMA	-21.22	5.73	65.77	No	Yes
S6-D6	Left-Frontal Eye Fields, Left-PreMotor and SMA	-38.52	20.16	50.11	No	Yes
S6-D15	Left-Frontal Eye Fields, Left-PreMotor and SMA	-6.07	24.17	61.17	No	Yes
S6-D17	Left-Frontal Eye Fields, Left-PreMotor and SMA	-15.37	23.96	52.29	No	Yes

S7-D5	Left-PreMotor and SMA, Left-Broca's Area	-63.58	-2.4	12.72	Yes	Yes
S7-D6	Left-PreMotor and SMA, Left-Broca's Area, Left-Frontal Eye Fields, Left-dlPFC	55.21	16.2	28.71	No	Yes
S7-D7	Left-Broca's Area, Left-Insula, Left-Pars Orbitalis, Left-Temporal Pole	-54.96	1.86	9.04	Yes	Yes
S7-D16	Left-Broca's Area, Left-dlPFC	-55.59	26.51	5.8	Yes	Yes
S7-D25	Short Channel	-60.25	12.04	19	No	No
S8-D7	Left-Temporal Pole, Left-Pars Orbitalis	-49.49	17.62	-21.58	No	No
S8-D16	Left-Broca's Area, Left-dlPFC, Left-Pars Orbitalis	-53.28	33.9	-6.56	Yes	Yes
S8-D26	Short Channel	-48.18	17.63	-21.3	No	No
S9-D8	Right-Visual Motor Cortex	30	-69.07	57.13	No	No
S9-D9	Right-Angular Gyrus, Right-Visual Association Cortex	50.12	-67.42	29.9	No	No
S9-D10	Right-Angular Gyrus, Right-Supramarginal Gyrus	33.13	-57.02	41.64	No	No
S10-D8	Right-Visual Motor Cortex, Right-Primary Sensory Cortex, Right-Sensory Association Cortex	25.02	-57.91	68.33	No	No
S10-D10	Right-Visual Motor Cortex, Right-Primary Sensory Cortex, Right-Sensory Association Cortex	28.6	-44.07	59.68	No	No
S10-D11	Right-Primary Sensory Cortex, Right-Primary Motor Cortex and SMA	23.85	-30.32	67.55	No	Yes
S11-D9	Right-Angular Gyrus	54.68	-52.61	20.58	No	No
S11-D10	Right-Angular Gyrus, Right-Supramarginal Gyrus	58.36	-44.57	36.09	No	Yes
S11-D12	Right-Primary Auditory Cortex, Right-Superior Temporal Gyrus, Right-Supramarginal Gyrus	42.58	-25.37	5.39	Yes	Yes
S12-D10	Right-Primary Sensory Cortex, Right- Supramarginal Gyrus, Right-Primary Sensory Cortex	48.85	-30.63	54.47	No	Yes
S12-D11	Right-PreMotor and SMA, Right-Primary Sensory Cortex	38.47	-13.49	62.4	No	Yes
S12-D12	Right-Primary Sensory Cortex, Right-Primary Motor Cortex and SMA	60.12	-13.96	35.05	No	Yes
S12-D13	Right-PreMotor and SMA, Right-Frontal Eye Fields,	41.89	-4.6	42.01	No	Yes

	Right-Primary Motor Cortex, Right-Broca's Area					
S13-D12	Right-Primary Auditory Cortex, Right-Superior Temporal Gyrus, Right-Supramarginal Gyrus	66.61	-18.8	5.53	Yes	Yes
S13-D14	Right-Medial Temporal Gyrus, Right-Superior Temporal Gyrus, Right-Temporal Pole	60.24	-1.62	-23.4	Yes	Yes
S13-D27	Short Channel	59.21	-14.17	-23.26	No	No
S14-D11	Right-PreMotor and SMA	20.39	4.31	48.6	No	Yes
S14-D13	Right-Frontal Eye Fields, Right-PreMotor and SMA	37.05	17.61	47.32	No	Yes
S14-D15	Right-Frontal Eye Fields, Right-PreMotor and SMA	18.24	15.36	59.12	No	Yes
S14-D18	Right-Frontal Eye Fields, Right-PreMotor and SMA	25.71	25.76	39.97	No	Yes
S14-D28	Short Channel	22.29	1.85	44	No	No
S15-D12	Right-Primary Motor Cortex, Right-Primary Sensory Cortex	61.89	-1.59	17.58	Yes	Yes
S15-D13	Right-Broca's Area, Right-Frontal Eye-Fields	56.55	16.68	28.63	No	Yes
S15-D14	Right Broca's Area, Right-Insula, Right-Temporal Pole, Right-Superior Temporal Gyrus	57.32	10.19	-10.39	Yes	Yes
S15-D19	Right Broca's Area, Right-dlPFC	55.46	29.42	7.58	Yes	Yes
S15-D29	Short Channel	37.79	19.35	-4.7	No	No
S16-D14	Right-Temporal Pole, Right-Pars Orbitalis	45.38	15.63	-26.23	No	No
S16-D19	Right-Pars Orbitalis, Right-Broca's Area	53.35	39.27	-3.8	Yes	Yes
S17-D4	Left-PreMotor and SMA	-24.76	-6.42	67.19	No	Yes
S17-D11	Right-PreMotor and SMA	19.15	-11.27	72.97	No	Yes
S17-D15	Left/Right-PreMotor and SMA	8.31	9.19	68.54	No	Yes
S18-D6	Left-Frontal Eye Fields, Left-dlPFC	-43.52	27.69	39.51	No	Yes
S18-D16	Left-Anterior Prefrontal Cortex, Left-dlPFC	-42.35	38.62	20.46	No	No
S18-D17	Left-dlPFC	-20.13	46.14	35.24	No	No
S19-D15	Left/Right-Frontal Eye Fields	3.19	-2.89	33.7	No	No
S19-D17	Left-Frontal Eye Fields, Left-dlPFC	-17.39	37.58	44.46	No	No
S19-D18	Right-Frontal Eye Fields, Right-dlPFC	14.55	40.87	46.16	No	No
S19-D20	Left/Right-dlPFC	-3.21	53.86	33.56	No	No

S20-D13	Right-Frontal Eye Fields, Right-PreMotor and SMA, Right-dlPFC	44.03	24.89	35.68	No	Yes
S20-D18	Right-dlPFC	25.55	42.59	28.6	No	No
S20-D19	Right-Frontal Eye Fields, Right-dlPFC	41.75	35.6	18	No	No
S21-D19	Right-dlPFC, Right-Anterior Prefrontal Cortex	35.92	41.74	-6.22	No	No
S21-D22	Right-Anterior Prefrontal Cortex	32.98	56.46	-13.52	No	No
S22-D19	Right-dlPFC, Right-Anterior Prefrontal Cortex	38.25	44.7	6.65	No	No
S22-D20	Right-Anterior Prefrontal Cortex	14.04	63.61	19.94	No	No
S22-D22	Right-Anterior Prefrontal Cortex	30.79	46.91	-7.75	No	No
S22-D30	Short Channel	31.43	56.65	10.84	No	No
S23-D16	Left-dlPFC, Left-Anterior Prefrontal Cortex	-37.68	52.3	10.42	No	No
S23-D20	Left-Anterior Prefrontal Cortex	-14.71	61.28	20.03	No	No
S23-D21	Left-Anterior Prefrontal Cortex	-29.87	-57.93	-2.5	No	No
S24-D16	Left-dlPFC, Left-Anterior Prefrontal Cortex	46.32	50.12	-2.45	No	No
S24-D21	Left-Anterior Prefrontal Cortex	-34.53	55.39	-14.21	No	No

Note. Cortical region assignments and Montreal Neurological Institute (MNI) coordinates were derived from optode projections generated in Satori (NIRx/Brain Innovation) using the standard template brain. Coordinates reflect the estimated cortical projection of each channel midpoint.

Table S3

Individual FDR Thresholds For the Listen Task.

Participant	HbO FDR Threshold	HbR FDR Threshold
pp1	0.0010	0.0022
pp2	0.0047	0.0032
pp3	0.1433	0.1018
pp4	0.0595	0.0528
pp5	0.0070	0.0184
pp6	0.0206	0.0180
pp7	0.0016	0.0010
pp8	0.0295	0.0259
pp9	0.0124	0.0005

pp10	0.0080	0.0289
pp11	0.1049	0.0120
pp12	0.0706	0.0003
pp13	0.1233	0.0042
pp14	0.0236	0.0000
pp15	0.0004	0.0044
pp16	0.0297	0.0492
pp17	0.2455	0.0048
pp18	0.0096	0.0036
pp19	0.0074	0.0007
pp20	0.0054	0.0066
pp21	0.0301	0.0435
pp22	0.0163	0.0001
pp23	0.0186	0.0009
pp24	0.0041	0.0013
pp25	0.0070	0.0000
pp26	0.0271	0.0020
pp27	0.0033	0.0008

Table S4

Individual FDR Thresholds For the Speak Task.

Participant	HbO FDR Threshold	HbR FDR Threshold
pp1	0.0240	0.0139
pp2	0.0136	0.0094
pp3	0.0316	0.0308
pp4	0.0013	0.0051
pp5	0.0131	0.0245
pp6	0.0067	0.0072
pp7	0.0585	0.5565
pp8	0.0291	0.0216

pp9	0.0144	0.0002
pp10	0.0194	0.0091
pp11	0.0033	0.0000
pp12	0.0055	0.0068
pp13	0.0157	0.0157
pp14	0.0258	0.0086
pp15	0.0093	0.0171
pp16	0.0013	0.1021
pp17	0.0129	0.0027
pp18	0.0165	0.0157
pp19	0.0302	0.0119
pp20	0.0114	0.0277
pp21	0.0400	0.0133
pp22	0.0191	0.0221
pp23	0.0393	0.0188
pp24	0.0005	0.0253
pp25	0.0194	0.0151
pp26	0.0347	0.0161
pp27	0.0196	0.0072

Table S5

Single-Subject Channel-Level Significance for HbO During the Listen Task

Partici-pant	Channel 1	Channel 2	Channel 3	Channel 4	Channel 5	Channel 6	Channel 7	Channel 8	Channel 9	Channel 10	Channel 11	Channel 12	Channel 13	Channel 14
	S3-D5	S5-D5	S5-D7	S7-D5	S7-D7	S7-D16	S8-D16	S11-D12	S13-D12	S13-D14	S15-D12	S15-D14	S15-D19	S16-D19
pp1	✘	✘	✘	✘	✘	✘	✘	✘	✔	✘	✘	✘	✘	✘
pp2	✔	✘	✘	✘	✘	✘	✘	✘	✘	✘	✘	✘	✘	✘
pp3	✘	✘	-	✘	-	✘	✘	✘	✔	✘	✘	✘	✘	✘
pp4	✘	✘	✘	✘	✘	✘	✘	✘	✘	✘	✘	✘	✘	✔
pp5	✘	✘	-	✘	✘	✔	-	✘	✔	✘	✘	✘	✘	✘
pp6	-	✘	✘	-	-	✘	-	✔	-	✘	-	-	-	-
pp7	-	-	-	-	-	-	✘	✔	-	-	-	-	-	✘
pp8	✔	✔	✔	✔	✘	✔	✘	-	-	✔	-	✘	✔	✔
pp9	✔	✘	✘	✘	✘	✘	✘	✔	✔	✔	✘	✘	-	✘
pp10	✘	✘	✘	✘	✘	✘	✘	✘	✘	✘	✘	✘	✘	✘
pp11	-	-	-	-	-	-	✘	-	-	-	-	-	-	✘
pp12	-	✘	✘	✘	✘	✘	✘	✘	✘	✘	✘	✘	✘	✔
pp13	-	✘	✘	✘	✘	✘	✘	✘	✘	✘	✘	✘	✘	✘
pp14	✔	✔	✔	✘	✘	✘	✘	✔	✘	✔	✘	✘	✔	✔
pp15	✘	✔	✘	-	-	-	✘	✘	✘	✔	✘	✘	✘	-
pp16	-	-	-	-	-	-	✘	-	-	✘	-	-	-	✘
pp17	✘	✘	✘	-	✘	✘	-	✘	✘	✘	-	✘	-	-
pp18	✘	✘	✘	✘	✘	-	✘	✘	✘	✘	✘	✘	-	✘
pp19	✘	✘	✘	✘	✘	✘	✘	✘	✔	✔	✘	✘	✘	✘
pp20	✘	✔	✔	✘	✘	✘	✘	✘	✘	✔	✘	✘	✘	✘

Participant	Channel 1<br>S3-D5	Channel 2<br>S5-D5	Channel 3<br>S5-D7	Channel 4<br>S7-D5	Channel 5<br>S7-D7	Channel 6<br>S7-D16	Channel 7<br>S8-D16	Channel 8<br>S11-D12	Channel 9<br>S13-D12	Channel 10<br>S13-D14	Channel 11<br>S15-D12	Channel 12<br>S15-D14	Channel 13<br>S15-D19	Channel 14<br>S16-D19
pp21	✔	-	-	✘	-	✘	-	✘	-	-	✘	-	-	-
pp22	✔	✘	✘	✘	-	✘	-	✘	✘	✘	✘	✘	✘	-
pp23	✘	-	-	✔	✘	✘	-	✔	✔	✘	✘	✔	✔	-
pp24	✘	-	✘	✘	✘	✘	✘	✘	✘	✘	✘	✘	✘	✘
pp25	✘	✘	✘	✘	✘	✘	✘	✔	✔	✔	✘	✘	✘	✔
pp26	✔	✔	✘	-	✘	-	✘	✘	✔	✔	✔	✔	✘	✘
pp27	✘	✘	✔	✘	✘	✘	✘	✘	✘	✔	✘	✘	✘	✘

Note. ✔ indicates that the channel met the participant-specific false discovery rate (FDR)–corrected significance threshold and showed an effect in the expected direction (positive HbO response). ✘ indicates that the channel did not meet the corrected threshold. — indicates that the channel was excluded based on the scalp coupling index (SCI) criterion. FDR correction was applied separately to HbO within each participant using the Benjamini–Hochberg procedure.

Table S6

Single-Subject Channel-Level Significance for HbR During the Listen Task

Participant	Channel 1<br>S3-D5	Channel 2<br>S5-D5	Channel 3<br>S5-D7	Channel 4<br>S7-D5	Channel 5<br>S7-D7	Channel 6<br>S7-D16	Channel 7<br>S8-D16	Channel 8<br>S11-D12	Channel 9<br>S13-D12	Channel 10<br>S13-D14	Channel 11<br>S15-D12	Channel 12<br>S15-D14	Channel 13<br>S15-D19	Channel 14<br>S16-D19
pp1	✘	✔	✘	✘	✘	✘	✘	✘	✔	✔	✘	✘	✘	✘
pp2	✔	✔	✘	✘	✘	✘	✘	✔	✔	✔	✘	✘	✘	✘
pp3	✘	✘	-	✘	-	✘	✘	✘	✘	✘	✘	✘	✘	✘
pp4	✘	✘	✘	✘	✘	✘	✘	✘	✘	✔	✘	✘	✘	✘
pp5	✘	✘	-	✘	✘	✔	-	✘	✔	✔	✘	✘	✘	✔
pp6	-	✘	✘	-	-	✘	-	✘	-	✘	-	-	-	-

Partici-pant	Channel 1	Channel 2	Channel 3	Channel 4	Channel 5	Channel 6	Channel 7	Channel 8	Channel 9	Channel 10	Channel 11	Channel 12	Channel 13	Channel 14
	S3-D5	S5-D5	S5-D7	S7-D5	S7-D7	S7-D16	S8-D16	S11-D12	S13-D12	S13-D14	S15-D12	S15-D14	S15-D19	S16-D19
pp7	-	-	-	-	-	-	✘	✔	-	-	-	-	-	✘
pp8	✘	✔	✔	✘	✘	✔	✘	-	-	✔	-	✔	✘	✘
pp9	✘	✘	✘	✘	✘	✘	✘	✔	✔	✘	✘	✘	-	✘
pp10	✘	✔	✔	✘	✔	✘	✔	✔	✔	✔	✘	✘	✔	✔
pp11	-	-	-	-	-	-	✘	-	-	-	-	-	-	✔
pp12	-	✘	✘	✘	✘	✘	✘	✘	✘	✔	✘	✘	✘	✘
pp13	-	✘	✘	✘	✘	✘	✘	✘	✘	✔	✘	✘	✘	✘
pp14	✘	✔	✘	✘	✘	✘	✘	✘	✘	✘	✘	✘	✘	✘
pp15	✘	✔	✘	-	-	-	✘	✘	✘	✔	✘	✘	✘	-
pp16	-	-	-	-	-	-	✘	-	-	✘	-	-	-	✔
pp17	✘	✘	✘	-	✘	✘	-	✘	✘	✔	-	✔	-	-
pp18	✘	✘	✘	✘	✘	-	✘	✘	✘	✘	✘	✔	-	✘
pp19	✘	✘	✘	✘	✘	✘	✘	✘	✔	✔	✘	✘	✘	✔
pp20	✘	✘	✘	✔	✘	✘	✘	✘	✘	✔	✘	✘	✘	✘
pp21	✘	-	-	✘	-	✘	-	✘	-	-	✘	-	-	-
pp22	✘	✘	✘	✘	-	✘	-	✘	✔	✔	✘	✘	✘	-
pp23	✘	-	-	✘	✘	✘	-	✘	✔	✘	✘	✘	✘	-
pp24	✘	-	✘	✘	✘	✘	✘	✘	✘	✘	✘	✘	✘	✘
pp25	✘	✘	✘	✘	✘	✘	✘	✔	✔	✘	✘	✘	✘	✘
pp26	✘	✔	✘	-	✘	-	✘	✘	✔	✔	✘	✔	✘	✘
pp27	✘	✘	✘	✘	✘	✘	✘	✘	✔	✔	✘	✘	✘	✘

Note. ✔ indicates that the channel met the participant-specific false discovery rate (FDR)–corrected significance threshold and showed an effect in the expected direction (negative HbR response). ✘ indicates that the channel did not meet the corrected threshold. — indicates that the channel was excluded based on the scalp coupling index (SCI) criterion. FDR correction was applied separately to HbR within each participant using the Benjamini–Hochberg procedure.

Table S7a

Single-Subject Channel-Level Significance for HbO During the Speak Task (Channel 1 - 10)

participant	Channel 1	Channel 2	Channel 3	Channel 4	Channel 5	Channel 6	Channel 7	Channel 8	Channel 9	Channel 10
	S2-D4	S3-D3	S3-D5	S4-D3	S4-D4	S4-D5	S4-D6	S5-D5	S5-D7	S6-D4
pp1	✘	✔	✔	✘	✘	✔	✘	✔	✘	-
pp2	-	✘	✘	-	-	✘	-	✘	-	✘
pp3	✔	✔	✔	-	✔	✔	✔	✔	✔	✘
pp4	-	-	✘	-	-	✘	-	✘	✘	-
pp5	✘	✘	✘	✔	✘	✔	✘	✘	✘	✘
pp6	-	-	-	-	-	-	-	✘	✘	-
pp7	-	-	-	-	-	-	-	-	-	-
pp8	✔	✔	✔	✔	✘	✔	✔	✔	✘	✔
pp9	-	-	✘	-	-	✔	-	✘	✘	-
pp10	-	-	✘	✘	-	-	✘	✘	✘	-
pp11	-	-	-	-	-	-	-	-	✘	-
pp12	-	-	-	-	-	✘	-	✘	✘	-
pp13	-	-	-	✘	-	✔	-	✔	✘	-
pp14	✘	✘	✔	✘	✘	✔	✘	✔	-	✘
pp15	-	-	✘	✘	✘	✘	✘	✔	✘	-
pp16	-	-	-	-	-	-	-	-	-	-
pp17	-	✘	✘	✘	-	✘	✘	✔	✔	-

participant	Channel 1	Channel 2	Channel 3	Channel 4	Channel 5	Channel 6	Channel 7	Channel 8	Channel 9	Channel 10
	S2-D4	S3-D3	S3-D5	S4-D3	S4-D4	S4-D5	S4-D6	S5-D5	S5-D7	S6-D4
pp18	-	-	✘	✔	-	✔	✔	✔	✘	✘
pp19	-	✘	✘	✘	✘	✘	✘	✘	✘	-
pp20	-	✘	✘	✘	✘	✔	✘	✘	✘	-
pp21	✘	✘	✘	✘	✘	✘	✘	✘	✘	✘
pp22	✘	✘	✘	✘	✘	✔	✔	✘	✘	✔
pp23	-	✔	✔	✔	✔	✔	✔	-	-	✔
pp24	-	-	-	-	-	✘	-	-	✘	-
pp25	✔	-	✘	✔	✔	✘	✔	✘	✘	✔
pp26	-	-	✔	✔	✔	✘	✔	✘	✘	-
pp27	-	-	✘	✘	-	✔	✘	✘	✔	-

Note. ✔ indicates that the channel met the participant-specific false discovery rate (FDR)–corrected significance threshold and showed an effect in the expected direction (positive HbO response). ✘ indicates that the channel did not meet the corrected threshold. — indicates that the channel was excluded based on the scalp coupling index (SCI) criterion. FDR correction was applied separately to HbO within each participant using the Benjamini–Hochberg procedure.

Table S7b

Single-Subject Channel-Level Significance for HbO During the Speak Task (Channel 11-20)

participant	Channel 11	Channel 12	Channel 13	Channel 14	Channel 15	Channel 16	Channel 17	Channel 18	Channel 19	Channel 20
	S6-D6	S6-D15	S6-D17	S7-D5	S7-D6	S7-D7	S7-D16	S8-D16	S10-D11	S11-D10
pp1	✘	✔	✘	✘	-	✘	✘	✘	✔	✔
pp2	-	-	-	✘	-	✘	✘	✘	✘	✘
pp3	✘	✔	✘	✔	✔	✘	✘	-	-	-
pp4	-	-	-	✘	✘	✘	✘	✘	-	✘

participant	Channel 11	Channel 12	Channel 13	Channel 14	Channel 15	Channel 16	Channel 17	Channel 18	Channel 19	Channel 20
	S6-D6	S6-D15	S6-D17	S7-D5	S7-D6	S7-D7	S7-D16	S8-D16	S10-D11	S11-D10
pp5	✘	✘	✘	✘	✘	✘	✘	✔	✘	✘
pp6	✘	-	-	-	-	✘	✘	-	-	-
pp7	-	-	-	-	-	-	-	✘	-	✘
pp8	-	✔	✔	✔	✔	✔	✔	-	✘	✘
pp9	-	-	-	✘	-	✘	✘	-	-	✘
pp10	-	-	-	✔	✔	✔	✔	✔	-	✘
pp11	-	✘	-	-	-	-	-	✘	-	-
pp12	-	✘	-	✘	-	✘	✔	✘	-	✘
pp13	-	-	-	✔	✘	✘	✔	✘	-	-
pp14	-	-	✘	✔	✔	-	✔	-	✔	✘
pp15	-	✘	✘	-	-	-	-	✘	-	✘
pp16	-	✔	-	-	-	-	-	✔	-	-
pp17	-	-	-	-	-	✘	✘	-	-	✘
pp18	✘	✔	✘	✔	-	-	-	✔	-	✘
pp19	✘	✘	✘	✘	✘	✘	✘	✘	-	✘
pp20	✘	-	✘	✘	✘	✘	✘	-	✘	✘
pp21	✘	✘	✘	✘	✘	✘	✘	✘	✘	✘
pp22	✔	✔	✔	✔	✔	✔	✔	-	✘	✘
pp23	✔	-	-	✔	✔	✔	✔	-	✔	✘
pp24	-	✘	-	✘	✘	✘	✘	✘	-	-
pp25	✘	✔	✘	✔	✘	✔	✘	✘	✔	✘
pp26	✔	✔	✔	✘	-	✔	-	✘	-	✔
pp27	✘	✘	-	✔	-	✘	-	✔	-	✘

Note. ✔ indicates that the channel met the participant-specific false discovery rate (FDR)–corrected significance threshold and showed an effect in the expected direction (positive HbO response). ✘ indicates that the channel did not meet the corrected threshold. — indicates that the channel was excluded based on the scalp coupling index (SCI) criterion. FDR correction was applied separately to HbO within each participant using the Benjamini–Hochberg procedure.

Table S7c

Single-Subject Channel-Level Significance for HbO During the Speak Task (Channel 21-30)

participant	Channel 21	Channel 22	Channel 23	Channel 24	Channel 25	Channel 26	Channel 27	Channel 28	Channel 29	Channel 30
	S11-D12	S12-D10	S12-D11	S12-D12	S12-D13	S13-D12	S13-D14	S14-D11	S14-D13	S14-D15
pp1	✔	✔	-	✔	✔	✘	✘	-	-	✔
pp2	✘	✘	✘	✘	✘	✘	✘	✘	✘	✘
pp3	✔	✔	✔	✔	✔	✔	✘	-	✘	✘
pp4	✘	-	-	✘	✔	✘	✘	-	-	-
pp5	✘	✔	✘	✘	✔	✘	✔	✘	✘	✘
pp6	✘	-	-	-	-	-	✘	-	-	-
pp7	✔	-	-	-	-	-	-	-	-	-
pp8	-	✔	✔	-	✔	-	✔	✘	✔	✔
pp9	✘	-	-	-	-	✘	✘	-	-	-
pp10	✘	✘	-	✘	✔	✘	✘	-	-	-
pp11	-	-	-	-	-	-	✘	-	-	✘
pp12	✘	✘	-	✘	✘	✘	✘	-	-	-
pp13	✘	-	-	-	-	✘	✘	-	-	-
pp14	✔	✔	✔	✔	✔	✘	✘	✘	✘	✔
pp15	✘	-	-	✘	-	✔	✔	-	-	-
pp16	-	-	-	-	-	-	✘	-	-	✔
pp17	✘	-	-	✘	✘	✔	✘	-	-	-

participant	Channel 21	Channel 22	Channel 23	Channel 24	Channel 25	Channel 26	Channel 27	Channel 28	Channel 29	Channel 30
	S11-D12	S12-D10	S12-D11	S12-D12	S12-D13	S13-D12	S13-D14	S14-D11	S14-D13	S14-D15
pp18	✔	-	-	✔	✘	✔	✘	-	-	-
pp19	✘	✘	-	✘	-	✘	✘	-	-	-
pp20	✘	-	✘	✔	✔	✘	✘	-	✘	-
pp21	✘	✘	✘	✘	✘	✘	✘	✘	✘	✘
pp22	✘	✔	✘	✔	✘	✘	✘	✔	✔	✔
pp23	✘	✔	✔	✔	✔	-	✔	-	✔	✘
pp24	✘	-	-	✘	-	✘	✘	-	✔	✘
pp25	✔	✔	✔	✘	✔	✔	✘	✔	✔	✔
pp26	✔	✔	-	✔	✔	✔	✘	-	✔	-
pp27	✔	-	-	✔	✘	✘	✘	-	-	-

Note. ✔ indicates that the channel met the participant-specific false discovery rate (FDR)–corrected significance threshold and showed an effect in the expected direction (positive HbO response). ✘ indicates that the channel did not meet the corrected threshold. — indicates that the channel was excluded based on the scalp coupling index (SCI) criterion. FDR correction was applied separately to HbO within each participant using the Benjamini–Hochberg procedure.

Table S7d

Single-Subject Channel-Level Significance for HbO During the Speak Task (Channel 31-41)

participant	Channel 31	Channel 32	Channel 33	Channel 34	Channel 35	Channel 36	Channel 37	Channel 38	Channel 39	Channel 40	Channel 41
	S14-D18	S15-D12	S15-D13	S15-D14	S15-D19	S16-D19	S17-D4	S17-D11	S17-D15	S18-D6	S20-D13
pp1	✘	✘	-	✘	-	✔	✔	✔	-	✘	✘
pp2	✘	✘	✘	✘	✘	✘	✘	✘	✘	-	✘
pp3	✘	-	✔	✘	✔	-	✘	✔	✔	✔	✘
pp4	-	✘	✘	✘	✘	✘	-	-	-	-	✔

participant	Channel 31	Channel 32	Channel 33	Channel 34	Channel 35	Channel 36	Channel 37	Channel 38	Channel 39	Channel 40	Channel 41
	S14-D18	S15-D12	S15-D13	S15-D14	S15-D19	S16-D19	S17-D4	S17-D11	S17-D15	S18-D6	S20-D13
pp5	✘	✘	✘	✘	✘	✔	✘	✔	✔	✘	✘
pp6	-	-	-	✘	-	-	-	✘	✘	✘	-
pp7	-	-	-	-	-	✘	-	-	-	-	-
pp8	✔	-	✔	✔	✔	-	✔	✘	✔	✔	✔
pp9	-	✘	-	✘	✘	✘	-	-	-	-	-
pp10	-	✘	✔	✘	✘	-	-	-	-	✘	✘
pp11	-	-	-	-	-	✘	✘	✘	✘	-	-
pp12	-	✘	✔	✘	✘	✘	-	-	-	-	✘
pp13	-	✘	✘	-	✘	✘	-	-	-	✘	✘
pp14	✘	-	✘	✘	✘	-	✘	✘	-	✘	✔
pp15	-	✘	✘	✔	✘	-	-	-	✘	-	✘
pp16	-	-	-	-	-	✔	-	✔	-	-	-
pp17	-	-	-	✘	-	-	-	✘	-	✘	✘
pp18	-	✘	✘	✘	-	✘	-	✔*	-	✔	✘
pp19	✘	✘	-	✘	✘	✘	-	-	-	✘	-
pp20	-	✘	✘	✘	✘	✔	-	-	-	✘	✘
pp21	✘	✘	✘	✘	✘	✘	✘	✘	✘	✘	✘
pp22	✔	✘	-	✘	✘	-	✔	✔	✔	✔	✔
pp23	✔	✔	✔	✔	✔	-	✔	-	-	-	✘
pp24	-	✘	✘	✘	✘	-	✘	✘	-	✔	✘
pp25	✔	✘	✔	✔	✘	✘	✔	✔	✔	✘	✔
pp26	✔	✔	✔	✔	✔	✘	-	-	-	✔	-
pp27	-	✔	✘	✘	✘	✔	-	-	✘	✘	✔

Note. ✔ indicates that the channel met the participant-specific false discovery rate (FDR)–corrected significance threshold and showed an effect in the expected direction (positive HbO response). ✘ indicates that the channel did not meet the corrected threshold. — indicates that the channel was excluded based on the scalp coupling index (SCI) criterion. FDR correction was applied separately to HbO within each participant using the Benjamini–Hochberg procedure.

Table S8a

Single-Subject Channel-Level Significance for HbR During the Speak Task (Channel 1-10)

participant	Channel 1	Channel 2	Channel 3	Channel 4	Channel 5	Channel 6	Channel 7	Channel 8	Channel 9	Channel 10
	S2-D4	S3-D3	S3-D5	S4-D3	S4-D4	S4-D5	S4-D6	S5-D5	S5-D7	S6-D4
pp1	✘	✘	✔	✘	✘	✔	✘	✔	✘	-
pp2	-	✘	✘	-	-	✘	-	✘	-	✘
pp3	✔	✔	✔	-	✔	✔	✔	✘	✘	✔
pp4	-	-	✘	-	-	✘	-	✘	✘	-
pp5	✔	✘	✘	✘	✔	✘	✘	✔	✔	✔
pp6	-	-	-	-	-	-	-	✘	✘	-
pp7	-	-	-	-	-	-	-	-	-	-
pp8	✘	✘	✘	✔	✘	✔	✔	✔	✘	✘
pp9	-	-	✘	-	-	✘	-	✘	✘	-
pp10	-	-	✘	✘	-	-	✔	✘	✘	-
pp11	-	-	-	-	-	-	-	-	✘	-
pp12	-	-	-	-	-	✘	-	✘	✘	-
pp13	-	-	-	✘	-	✔	-	✘	✘	-
pp14	✘	✘	✔	✘	✘	✔	✔	✔	-	✔
pp15	-	-	✘	✘	✔	✘	✔	✔	✔	-
pp16	-	-	-	-	-	-	-	-	-	-
pp17	-	✘	✘	✘	-	✘	✘	✔	✔	-

participant	Channel 1	Channel 2	Channel 3	Channel 4	Channel 5	Channel 6	Channel 7	Channel 8	Channel 9	Channel 10
	S2-D4	S3-D3	S3-D5	S4-D3	S4-D4	S4-D5	S4-D6	S5-D5	S5-D7	S6-D4
pp18	-	-	✔	✘	-	✔	✘	✔	✘	✘
pp19	-	✘	✘	✘	✘	✘	✘	✘	✘	-
pp20	-	✔	✔	✔	✘	✔	✔	✘	✘	-
pp21	✘	✘	✘	✘	✘	✘	✘	✘	✘	✘
pp22	✘	✘	✘	✘	✘	✘	✘	✔	✘	✘
pp23	-	✘	✘	✘	✘	✔	✘	-	-	✘
pp24	-	-	-	-	-	✔	-	-	✘	-
pp25	✔	-	✘	✘	✘	✘	✔	✘	✘	✔
pp26	-	-	✔	✘	✘	✘	✘	✘	✘	-
pp27	-	-	✔	✘	-	✔	✔	✘	✔	-

Note. ✔ indicates that the channel met the participant-specific false discovery rate (FDR)–corrected significance threshold and showed an effect in the expected direction (negative HbR response). ✘ indicates that the channel did not meet the corrected threshold. — indicates that the channel was excluded based on the scalp coupling index (SCI) criterion. FDR correction was applied separately to HbR within each participant using the Benjamini–Hochberg procedure.

Table S8b

Single-Subject Channel-Level Significance for HbR During the Speak Task (Channel 11-20)

participant	Channel 11	Channel 12	Channel 13	Channel 14	Channel 15	Channel 16	Channel 17	Channel 18	Channel 19	Channel 20
	S6-D6	S6-D15	S6-D17	S7-D5	S7-D6	S7-D7	S7-D16	S8-D16	S10-D11	S11-D10
pp1	✘	✘	✘	✘	-	✔	✘	✘	✘	✘
pp2	-	-	-	✔	-	✘	✘	✘	✘	✘
pp3	✘	✔	✔	✔	✘	✘	✘	-	-	-
pp4	-	-	-	✘	✘	✘	✘	✘	-	✘

participant	Channel 11	Channel 12	Channel 13	Channel 14	Channel 15	Channel 16	Channel 17	Channel 18	Channel 19	Channel 20
	S6-D6	S6-D15	S6-D17	S7-D5	S7-D6	S7-D7	S7-D16	S8-D16	S10-D11	S11-D10
pp5	✔	✔	✘	✘	✔	✔	✘	✘	✔	✘
pp6	✘	-	-	-	-	✔	✘	-	-	-
pp7	-	-	-	-	-	-	-	✘	-	✔
pp8	-	✔	✘	✘	✘	✔	✔	-	✘	✘
pp9	-	-	-	✘	-	✘	✘	-	-	✘
pp10	-	-	-	✔	✔	✔	✘	✘	-	✘
pp11	-	✔	-	-	-	-	-	✘	-	-
pp12	-	✘	-	✘	-	✘	✔	✘	-	✘
pp13	-	-	-	✔	✘	✘	✘	✘	-	-
pp14	-	-	✘	✔	✔	-	✔	-	✘	✘
pp15	-	✘	✘	-	-	-	-	✘	-	✘
pp16	-	✘	-	-	-	-	-	✘	-	-
pp17	-	-	-	-	-	✘	✘	-	-	✔
pp18	✘	✔	✘	✘	-	-	-	✔	-	✘
pp19	✘	✘	✘	✘	✘	✘	✘	✘	-	✘
pp20	✘	-	✘	✔	✘	✔	✘	-	✘	✘
pp21	✘	✘	✘	✔	✘	✘	✘	✘	✘	✘
pp22	✘	✘	✘	✔	✔	✔	✔	-	✘	✘
pp23	✔	-	-	✔	✔	✔	✔	-	✘	✘
pp24	-	✘	-	✘	✔	✘	✘	✘	-	-
pp25	✔	✘	✘	✘	✘	✘	✘	✘	✔	✔
pp26	✔	✘	✔	✘	-	✘	-	✘	-	✘
pp27	✘	✘	-	✔	-	✘	-	✘	-	✘

Note. ✔ indicates that the channel met the participant-specific false discovery rate (FDR)–corrected significance threshold and showed an effect in the expected direction (negative HbR response). ✘ indicates that the channel did not meet the corrected threshold. — indicates that the channel was excluded based on the scalp coupling index (SCI) criterion. FDR correction was applied separately to HbR within each participant using the Benjamini–Hochberg procedure.

Table S8c

Single-Subject Channel-Level Significance for HbR During the Speak Task (Channel 21-30)

participant	Channel 21	Channel 22	Channel 23	Channel 24	Channel 25	Channel 26	Channel 27	Channel 28	Channel 29	Channel 30
	S11-D12	S12-D10	S12-D11	S12-D12	S12-D13	S13-D12	S13-D14	S14-D11	S14-D13	S14-D15
pp1	✔	✘	-	✔	✘	✔	✘	-	-	✘
pp2	✘	✘	✔	✘	✘	✘	✘	✘	✘	✘
pp3	✔	✔	✔	✔	✔	✔	✔	-	✔	✔
pp4	✘	-	-	✘	✘	✘	✘	-	-	-
pp5	✔	✘	✔	✔	✘	✔	✔	✘	✘	✔
pp6	✘	-	-	-	-	-	✘	-	-	-
pp7	✘	-	-	-	-	-	-	-	-	-
pp8	-	✘	✘	-	✔	-	✔	✘	✔	✘
pp9	✘	-	-	-	-	✘	✘	-	-	-
pp10	✔	✘	-	✘	✘	✔	✔	-	-	-
pp11	-	-	-	-	-	-	✘	-	-	✔
pp12	✘	✘	-	✘	✘	✔	✔	-	-	-
pp13	✘	-	-	-	-	✘	✔	-	-	-
pp14	✔	✘	✘	✘	✔	✘	✘	✘	✘	✔
pp15	✘	-	-	✘	-	✔	✔	-	-	-
pp16	-	-	-	-	-	-	✘	-	-	✘
pp17	✘	-	-	✘	✘	✘	✘	-	-	-

pp18	✔	-	-	✔	✘	✘	✔	-	-	-
pp19	✔	✘	-	✘	-	✘	✘	-	-	-
pp20	✔	-	✘	✘	✔	✔	✔	-	✔	-
pp21	✘	✘	✘	✔	✘	✘	✘	✘	✘	✘
pp22	✘	✘	✘	✔	✘	✘	✘	✘	✘	✘
pp23	✘	✘	✘	✘	✘	-	✔	-	✘	✔
pp24	✔	-	-	✔	-	✔	✔	-	✔	✘
pp25	✔	✔	✔	✘	✘	✔	✘	✔	✔	✔
pp26	✔	✘	-	✔	✔	✔	✘	-	✔	-
pp27	✔	-	-	✔	✔	✔	✔	-	-	-

Note. ✔ indicates that the channel met the participant-specific false discovery rate (FDR)–corrected significance threshold and showed an effect in the expected direction (negative HbR response). ✘ indicates that the channel did not meet the corrected threshold. — indicates that the channel was excluded based on the scalp coupling index (SCI) criterion. FDR correction was applied separately to HbR within each participant using the Benjamini–Hochberg procedure.

Table S8d

Single-Subject Channel-Level Significance for HbR During the Speak Task (Channel 31-41)

participant	Channel 31	Channel 32	Channel 33	Channel 34	Channel 35	Channel 36	Channel 37	Channel 38	Channel 39	Channel 40	Channel 41
	S14-D18	S15-D12	S15-D13	S15-D14	S15-D19	S16-D19	S17-D4	S17-D11	S17-D15	S18-D6	S20-D13
pp1	✘	✘	-	✔	-	✘	✘	✘	-	✘	✘
pp2	✘	✘	✘	✘	✘	✘	✘	✘	✘	-	✘
pp3	✔	-	✔	✘	✘	-	✔	✔	✔	✘	✘
pp4	-	✘	✔	✘	✘	✘	-	-	-	-	✘
pp5	✘	✔	✘	✘	✘	✔	✘	✘	✘	✘	✘
pp6	-	-	-	✘	-	-	-	✘	✘	✘	-
pp7	-	-	-	-	-	✘	-	-	-	-	-

participant	Channel 31	Channel 32	Channel 33	Channel 34	Channel 35	Channel 36	Channel 37	Channel 38	Channel 39	Channel 40	Channel 41
	S14-D18	S15-D12	S15-D13	S15-D14	S15-D19	S16-D19	S17-D4	S17-D11	S17-D15	S18-D6	S20-D13
pp8	✘	-	✔	✔	✘	-	✔	✘	✘	✘	✔
pp9	-	✘	-	✘	✘	✘	-	-	-	-	-
pp10	-	✔	✔	✔	✘	-	-	-	-	✘	✘
pp11	-	-	-	-	-	✘	✔	✔	✔	-	-
pp12	-	✘	✔	✘	✘	✘	-	-	-	-	✘
pp13	-	✘	✘	-	✘	✘	-	-	-	✘	✘
pp14	✘	-	✘	✘	✘	-	✔	✔	-	✘	✔
pp15	-	✘	✘	✘	✘	-	-	-	✔	-	✘
pp16	-	-	-	-	-	✘	-	✘	-	-	-
pp17	-	-	-	✘	-	-	-	✘	-	✘	✘
pp18	-	✘	✘	✘	-	✘	-	✘	-	✘	✔
pp19	✘	✘	-	✘	✘	✘	-	-	-	✘	-
pp20	-	✔	✔	✔	✔	✘	-	-	-	✘	✘
pp21	✘	✘	✘	✘	✘	✘	✘	✘	✔	✘	✘
pp22	✘	✔	-	✘	✔	-	✘	✘	✘	✘	✘
pp23	✘	✔	✘	✔	✔	-	✘	-	-	-	✘
pp24	-	✔	✔	✘	✔	-	✘	✔	-	✔	✔
pp25	✔	✘	✔	✘	✘	✘	✘	✔	✘	✘	✔
pp26	✘	✘	✔	✘	✘	✔	-	-	-	✘	-
pp27	-	✔	✔	✔	✔	✘	-	-	✘	✘	✔

Note. ✔ indicates that the channel met the participant-specific false discovery rate (FDR)–corrected significance threshold and showed an effect in the expected direction (negative HbR response). ✘ indicates that the channel did not meet the corrected threshold. — indicates that the channel was excluded based on the scalp coupling index (SCI) criterion. FDR correction was applied separately to HbR within each participant using the Benjamini–Hochberg procedure.

Table S9

Single-Subject GLM Results for HbO and HbR During the Listen Task (Participant pp1)

Channel	HbO Beta	HbO t	HbO p	HbR Beta	HbR t	HbR p
S3-D5	0.40531	1.40494	0.16012	-0.33639	-1.46638	0.14263
S5-D5	0.87593	3.17967	0.00149	-0.62770	-3.06644	0.00218
S5-D7	-0.03244	-0.15712	0.87516	-0.34750	-1.66517	0.09596
S7-D5	0.05426	0.16429	0.86951	-0.17221	-0.86718	0.38590
S7-D7	-0.20529	-0.68875	0.49102	-0.28541	-0.98145	0.32644
S7-D16	0.18547	0.52989	0.59622	-0.53398	-1.76064	0.07838
S8-D16	0.36369	1.39258	0.16383	-0.40763	-2.21323	0.02694
S11-D12	0.19044	0.61257	0.54020	-0.62037	-2.39478	0.01668
S13-D12	1.36373	3.74511	0.00018	-2.28876	-8.22935	0.00000
S13-D14	0.16609	0.65678	0.51136	-0.61838	-3.07754	0.00210
S15-D12	0.20657	0.69353	0.48802	-0.02776	-0.12975	0.89678
S15-D14	0.08054	0.29153	0.77066	-0.20853	-0.70891	0.47842
S15-D19	0.25227	0.79851	0.42463	-0.49226	-1.83942	0.06593
S16-D19	0.24075	1.00175	0.31653	-0.10675	-0.58673	0.55742

Note. Beta denotes the GLM regression coefficient. t and p values correspond to the contrast of interest (listen > baseline). Values are rounded to five decimal places. Only channels retained following the scalp coupling index (SCI) criterion are shown.

Table S10

Single-Subject GLM Results for HbO and HbR During the Listen Task (Participant pp2)

Channel	HbO Beta	HbO t	HbO p	HbR Beta	HbR t	HbR p
S3-D5	1.21875	3.83226	0.00013	-1.79671	-5.32845	0.00000
S5-D5	0.40486	1.07458	0.28263	-1.32106	-4.84818	0.00000
S5-D7	0.31664	1.00170	0.31655	0.09628	0.40505	0.68547
S7-D5	0.05346	0.19940	0.84196	0.04492	0.21255	0.83169
S7-D7	-0.31142	-1.08346	0.27867	-0.32607	-1.33417	0.18222
S7-D16	-0.00785	-0.03229	0.97424	0.02792	0.12990	0.89665
S8-D16	-0.38366	-0.91731	0.35903	0.10250	0.26906	0.78790

S11-D12	0.98701	2.82624	0.00473	-1.06487	-2.94982	0.00320
S13-D12	0.39390	0.97667	0.32879	-1.71946	-6.49179	0.00000
S13-D14	0.55792	1.70821	0.08768	-0.91585	-3.80050	0.00015
S15-D12	0.21312	0.70418	0.48136	-0.00708	-0.02335	0.98137
S15-D14	0.34809	1.23154	0.21819	-0.60955	-1.68089	0.09286
S15-D19	0.12882	0.46467	0.64219	-0.13507	-0.64415	0.51951
S16-D19	-0.12181	-0.38728	0.69857	0.02141	0.11854	0.90564

Note. Beta denotes the GLM regression coefficient. t and p values correspond to the contrast of interest (listen > baseline). Values are rounded to five decimal places. Only channels retained following the scalp coupling index (SCI) criterion are shown.

Table S11

Single-Subject GLM Results for HbO and HbR During the Listen Task (Participant pp3)

Channel	HbO Beta	HbO t	HbO p	HbR Beta	HbR t	HbR p
S3-D5	0.47894	1.19657	0.23155	0.14538	0.40132	0.68821
S5-D5	-0.03292	-0.10211	0.91868	-0.21748	-0.61084	0.54134
S7-D5	0.42515	1.07798	0.28111	-0.22159	-0.71066	0.47734
S7-D16	-0.04546	-0.11565	0.90794	-0.02076	-0.05753	0.95413
S8-D16	0.10010	0.24206	0.80875	0.01896	0.04850	0.96132
S11-D12	0.27083	0.75032	0.45311	0.05342	0.16371	0.86997
S13-D12	0.52394	1.46408	0.14326	-0.58009	-1.63648	0.10182
S13-D14	0.04219	0.16205	0.87128	-0.37491	-1.21291	0.22524
S15-D12	0.04933	0.12967	0.89683	-0.18064	-0.51494	0.60662
S15-D14	-0.03227	-0.13867	0.88972	-0.17246	-0.59365	0.55278
S15-D19	-0.28280	-0.73113	0.46474	0.04567	0.12488	0.90063
S16-D19	0.13703	0.39374	0.69380	-0.27646	-0.79482	0.42677

Note. Beta denotes the GLM regression coefficient. t and p values correspond to the contrast of interest (listen > baseline). Values are rounded to five decimal places. Only channels retained following the scalp coupling index (SCI) criterion are shown.

Table S12

Single-Subject GLM Results for HbO and HbR During the Listen Task (Participant pp4)

Channel	HbO Beta	HbO t	HbO p	HbR Beta	HbR t	HbR p
S3-D5	-0.11047	-0.25125	0.80164	-0.32140	-0.77683	0.43733
S5-D5	0.46192	1.38636	0.16575	-0.06364	-0.20578	0.83698
S5-D7	-0.03280	-0.14035	0.88839	-0.11577	-0.44309	0.65774
S7-D5	-0.55376	-1.54365	0.12279	-0.50291	-1.63764	0.10161
S7-D7	-0.14611	-0.51822	0.60435	-0.08161	-0.24829	0.80393
S7-D16	0.10926	0.40340	0.68669	-0.31104	-0.79631	0.42592
S8-D16	-0.01971	-0.06069	0.95161	-0.45257	-1.23099	0.21843
S11-D12	0.40775	1.05405	0.29195	-0.12883	-0.32221	0.74732
S13-D12	-0.25990	-0.70807	0.47897	-0.49611	-1.27790	0.20139
S13-D14	0.11335	0.49424	0.62118	-0.62695	-1.93754	0.05278
S15-D12	-0.13866	-0.41826	0.67579	-0.17961	-0.60457	0.54551
S15-D14	-0.37432	-1.38845	0.16512	-0.02740	-0.11144	0.91127
S15-D19	-0.03455	-0.12152	0.90329	-0.21027	-0.67841	0.49757
S16-D19	0.50235	1.88557	0.05946	-0.16988	-0.42914	0.66785

Note. Beta denotes the GLM regression coefficient. t and p values correspond to the contrast of interest (listen > baseline). Values are rounded to five decimal places. Only channels retained following the scalp coupling index (SCI) criterion are shown.

Table S13

Single-Subject GLM Results for HbO and HbR During the Listen Task (Participant pp5)

Channel	HbO Beta	HbO t	HbO p	HbR Beta	HbR t	HbR p
S3-D5	0.34928	1.06802	0.28558	-0.73362	-1.82925	0.06744
S5-D5	0.34473	1.21436	0.22469	-0.48215	-1.73613	0.08262
S7-D5	0.40919	1.14272	0.25323	0.07425	0.19258	0.84730
S7-D7	0.04348	0.15604	0.87601	-0.38476	-1.57290	0.11583
S7-D16	1.06141	3.18071	0.00148	-1.20357	-4.06894	0.00005
S11-D12	-0.63867	-1.59519	0.11075	0.41059	1.03654	0.30002
S13-D12	0.91824	2.69855	0.00700	-1.19647	-3.56447	0.00037
S13-D14	0.12108	0.54905	0.58301	-1.06792	-3.79255	0.00015
S15-D12	0.13680	0.39571	0.69234	0.28633	0.69312	0.48828

S15-D14	-0.23890	-1.24368	0.21370	0.25002	1.13657	0.25579
S15-D19	0.34502	1.16430	0.24438	-0.63141	-2.35798	0.01843
S16-D19	0.49288	2.18207	0.02917	-0.56691	-2.41293	0.01587

Note. Beta denotes the GLM regression coefficient. t and p values correspond to the contrast of interest (listen > baseline). Values are rounded to five decimal places. Only channels retained following the scalp coupling index (SCI) criterion are shown.

Table S14

Single-Subject GLM Results for HbO and HbR During the Listen Task (Participant pp6)

Channel	HbO Beta	HbO t	HbO p	HbR Beta	HbR t	HbR p
S5-D5	0.26493	0.92058	0.35733	-0.08924	-0.46516	0.64184
S5-D7	-0.11654	-0.57075	0.56821	0.04962	0.32325	0.74652
S7-D16	0.43326	1.21889	0.22297	0.68255	2.36763	0.01796
S11-D12	0.97067	2.31581	0.02062	-0.04202	-0.12355	0.90168
S13-D14	-0.08261	-0.34511	0.73003	-0.34524	-2.09959	0.03583

Note. Beta denotes the GLM regression coefficient. t and p values correspond to the contrast of interest (listen > baseline). Values are rounded to five decimal places. Only channels retained following the scalp coupling index (SCI) criterion are shown.

Table S15

Single-Subject GLM Results for HbO and HbR During the Listen Task (Participant pp7)

Channel	HbO Beta	HbO t	HbO p	HbR Beta	HbR t	HbR p
S8-D16	0.71793	1.64115	0.10085	-0.53658	-1.07506	0.28242
S11-D12	1.10647	3.16401	0.00157	-0.98854	-3.28506	0.00103
S16-D19	0.24484	0.59288	0.55330	-0.22777	-0.52077	0.60256

Note. Beta denotes the GLM regression coefficient. t and p values correspond to the contrast of interest (listen > baseline). Values are rounded to five decimal places. Only channels retained following the scalp coupling index (SCI) criterion are shown.

Table S16

Single-Subject GLM Results for HbO and HbR During the Listen Task (Participant pp8)

Channel	HbO Beta	HbO t	HbO p	HbR Beta	HbR t	HbR p
S3-D5	0.97596	2.17825	0.02945	-0.67937	-1.43838	0.15042

S5-D5	1.71328	4.57599	0.00000	-2.13119	-5.41369	0.00000
S5-D7	2.36321	6.73172	0.00000	-3.09246	-9.27619	0.00000
S7-D5	0.95637	3.12386	0.00180	-0.85512	-2.22859	0.02590
S7-D7	0.53323	1.48026	0.13889	-0.22248	-0.55789	0.57695
S7-D16	0.98074	3.04371	0.00235	-1.03331	-2.33430	0.01964
S8-D16	0.69820	1.69877	0.08945	-0.32403	-0.67725	0.49829
S13-D14	0.93236	3.08518	0.00205	-2.34900	-6.46419	0.00000
S15-D14	0.38968	1.31288	0.18931	-0.77697	-2.26490	0.02358
S15-D19	0.68568	2.28085	0.02262	-0.78942	-2.14690	0.03187
S16-D19	0.69436	2.43838	0.01480	-0.80383	-1.92714	0.05404

Note. Beta denotes the GLM regression coefficient. t and p values correspond to the contrast of interest (listen > baseline). Values are rounded to five decimal places. Only channels retained following the scalp coupling index (SCI) criterion are shown.

Table S17

Single-Subject GLM Results for HbO and HbR During the Listen Task (Participant pp9)

Channel	HbO Beta	HbO t	HbO p	HbR Beta	HbR t	HbR p
S3-D5	0.49655	2.58893	0.00966	-0.06057	-0.20691	0.83609
S5-D5	0.45804	1.96028	0.05004	-0.27717	-0.95286	0.34072
S5-D7	0.20260	0.65300	0.51380	-0.27544	-0.80181	0.42271
S7-D5	0.43131	2.02796	0.04263	0.05955	0.27957	0.77982
S7-D7	0.16623	0.74587	0.45579	-0.02785	-0.14764	0.88263
S7-D16	0.05080	0.21974	0.82609	-0.10678	-0.41915	0.67513
S8-D16	0.47267	1.33377	0.18236	-0.45174	-1.59983	0.10972
S11-D12	0.81152	2.50279	0.01236	-0.98108	-3.49685	0.00048
S13-D12	0.78245	3.35724	0.00079	-0.73924	-3.88695	0.00010
S13-D14	0.65436	2.73055	0.00635	-0.51088	-2.18391	0.02903
S15-D12	0.43293	1.60914	0.10767	0.02613	0.10659	0.91512
S15-D14	0.42489	1.58675	0.11265	-0.06842	-0.29844	0.76539
S16-D19	0.19547	1.14931	0.25050	-0.12305	-0.74630	0.45553

Note. Beta denotes the GLM regression coefficient. t and p values correspond to the contrast of interest (listen > baseline). Values are rounded to five decimal places. Only channels retained following the scalp coupling index (SCI) criterion are shown.

Table S18

Single-Subject GLM Results for HbO and HbR During the Listen Task (Participant pp10)

Channel	HbO Beta	HbO t	HbO p	HbR Beta	HbR t	HbR p
S3-D5	-0.64576	-1.39892	0.16192	-0.01092	-0.02514	0.97994
S5-D5	0.84541	2.43870	0.01479	-2.58207	-7.17795	0.00000
S5-D7	-0.12918	-0.38600	0.69952	-2.21360	-8.06166	0.00000
S7-D5	-0.53251	-1.24944	0.21158	-0.67859	-1.76490	0.07766
S7-D7	-0.35112	-0.86537	0.38689	-0.90751	-2.41833	0.01564
S7-D16	-0.46887	-1.20585	0.22795	-0.77759	-2.18532	0.02893
S8-D16	-0.41940	-1.06003	0.28920	-0.92118	-2.66688	0.00769
S11-D12	0.56196	1.38782	0.16528	-2.01564	-5.97075	0.00000
S13-D12	-0.53475	-1.31198	0.18961	-1.82555	-7.20388	0.00000
S13-D14	-0.79883	-2.65384	0.00799	-2.93902	-12.03130	0.00000
S15-D12	-0.48989	-1.22793	0.21955	-0.10494	-0.24136	0.80929
S15-D14	-0.83415	-2.32357	0.02020	-0.76231	-1.98169	0.04759
S15-D19	-0.20970	-0.63183	0.52754	-1.26350	-3.28106	0.00104
S16-D19	-0.28947	-0.83247	0.40520	-1.62130	-5.31210	0.00000

Note. Beta denotes the GLM regression coefficient. t and p values correspond to the contrast of interest (listen > baseline). Values are rounded to five decimal places. Only channels retained following the scalp coupling index (SCI) criterion are shown.

Table S19

Single-Subject GLM Results for HbO and HbR During the Listen Task (Participant pp11)

Channel	HbO Beta	HbO t	HbO p	HbR Beta	HbR t	HbR p
S8-D16	-0.34058	-1.62173	0.10495	-0.15932	-0.57314	0.56659
S16-D19	-0.05828	-0.30988	0.75667	-0.74430	-2.51351	0.01200

Note. Beta denotes the GLM regression coefficient. t and p values correspond to the contrast of interest (listen > baseline). Values are rounded to five decimal places. Only channels retained following the scalp coupling index (SCI) criterion are shown.

Table S20

Single-Subject GLM Results for HbO and HbR During the Listen Task (Participant pp12)

Channel	HbO Beta	HbO t	HbO p	HbR Beta	HbR t	HbR p
S5-D5	0.71261	1.74099	0.08177	0.22067	0.56447	0.57247
S5-D7	0.30575	0.72141	0.47070	-0.41677	-1.54305	0.12291
S7-D5	0.66593	1.63212	0.10274	-0.13609	-0.32023	0.74881
S7-D7	0.49156	1.10743	0.26818	-0.23845	-1.20318	0.22899
S7-D16	0.39458	0.81055	0.41768	-0.37122	-0.99391	0.32034
S8-D16	0.00080	0.00200	0.99840	-0.35057	-0.97269	0.33078
S11-D12	0.29556	0.64859	0.51664	-0.22855	-0.55630	0.57804
S13-D12	-0.51264	-1.26858	0.20467	0.46337	1.15366	0.24872
S13-D14	0.24052	0.94397	0.34525	-0.88279	-3.62375	0.00029
S15-D12	0.27742	0.81925	0.41270	0.05881	0.17486	0.86120
S15-D14	0.01846	0.08018	0.93610	-0.19122	-0.53781	0.59074
S15-D19	0.62475	1.42854	0.15322	-0.65089	-1.44262	0.14922
S16-D19	0.84398	1.80872	0.07058	-0.58828	-1.31606	0.18824

Note. Beta denotes the GLM regression coefficient. t and p values correspond to the contrast of interest (listen > baseline). Values are rounded to five decimal places. Only channels retained following the scalp coupling index (SCI) criterion are shown.

Table S21

Single-Subject GLM Results for HbO and HbR During the Listen Task (Participant pp13)

Channel	HbO Beta	HbO t	HbO p	HbR Beta	HbR t	HbR p
S5-D5	-0.06528	-0.28541	0.77534	-0.02831	-0.08107	0.93539
S5-D7	-0.08694	-0.35138	0.72532	-0.25596	-0.87901	0.37945
S7-D5	0.13328	0.32686	0.74379	-0.74280	-2.86188	0.00423
S7-D7	-0.01472	-0.05665	0.95483	0.28672	0.85361	0.39337
S7-D16	-0.52037	-1.34303	0.17934	0.39761	1.12750	0.25960
S8-D16	0.19814	0.57034	0.56848	0.31354	1.10841	0.26776
S11-D12	-0.15730	-0.58071	0.56147	-0.12988	-0.54142	0.58825
S13-D12	-0.42044	-1.54130	0.12333	-0.16466	-0.73029	0.46526

S13-D14	0.11727	0.48861	0.62515	-0.65855	-1.71101	0.08716
S15-D12	-0.39841	-1.50969	0.13121	-0.08891	-0.48086	0.63065
S15-D14	-0.15429	-0.60906	0.54252	-0.06258	-0.23773	0.81210
S15-D19	0.01964	0.05885	0.95307	-0.47677	-1.87891	0.06033
S16-D19	-0.02356	-0.07185	0.94272	0.05087	0.14647	0.88356

Note. Beta denotes the GLM regression coefficient. t and p values correspond to the contrast of interest (listen > baseline). Values are rounded to five decimal places. Only channels retained following the scalp coupling index (SCI) criterion are shown.

Table S22

Single-Subject GLM Results for HbO and HbR During the Listen Task (Participant pp14)

Channel	HbO Beta	HbO t	HbO p	HbR Beta	HbR t	HbR p
S3-D5	1.33237	4.14768	0.00003	-0.38540	-1.36198	0.17329
S5-D5	2.87678	7.56642	0.00000	-1.52528	-5.08632	0.00000
S5-D7	1.48341	5.54183	0.00000	0.55293	1.67377	0.09426
S7-D5	0.35440	1.02882	0.30363	0.13028	0.39751	0.69102
S7-D7	-0.49289	-1.33781	0.18104	0.78862	2.32364	0.02020
S7-D16	0.03983	0.13509	0.89255	-0.08604	-0.31220	0.75490
S8-D16	-0.75439	-2.45052	0.01431	-0.01481	-0.04975	0.96033
S11-D12	1.41779	5.04477	0.00000	-0.58948	-2.07029	0.03850
S13-D12	-0.05349	-0.16470	0.86919	0.13828	0.45601	0.64841
S13-D14	1.33613	3.79129	0.00015	0.45678	1.11905	0.26319
S15-D12	0.57582	2.26392	0.02364	0.43928	1.69387	0.09037
S15-D14	-0.72650	-2.42035	0.01555	1.02563	2.64776	0.00814
S15-D19	0.72288	2.99011	0.00281	-0.03466	-0.13349	0.89382
S16-D19	0.93975	2.68953	0.00719	-0.19680	-0.79389	0.42731

Note. Beta denotes the GLM regression coefficient. t and p values correspond to the contrast of interest (listen > baseline). Values are rounded to five decimal places. Only channels retained following the scalp coupling index (SCI) criterion are shown.

Table S23

Single-Subject GLM Results for HbO and HbR During the Listen Task (Participant pp15)

Channel	HbO Beta	HbO t	HbO p	HbR Beta	HbR t	HbR p
S3-D5	0.33983	1.01411	0.31060	-0.07192	-0.27122	0.78624
S5-D5	1.51438	4.12601	0.00004	-1.41512	-5.71035	0.00000
S5-D7	0.60128	2.07304	0.03823	-0.88803	-3.51458	0.00045
S8-D16	0.10335	0.31241	0.75474	0.41426	1.22981	0.21884
S11-D12	0.53032	1.62694	0.10383	-0.05976	-0.31972	0.74920
S13-D12	0.28896	1.02564	0.30512	-0.14580	-0.56058	0.57512
S13-D14	0.99082	3.54244	0.00040	-2.07736	-6.96583	0.00000
S15-D12	0.20843	0.68153	0.49558	-0.01013	-0.04158	0.96683
S15-D14	0.26605	0.89732	0.36960	-0.15663	-0.58831	0.55636
S15-D19	0.50758	1.79674	0.07245	-0.83169	-2.84855	0.00442

Note. Beta denotes the GLM regression coefficient. t and p values correspond to the contrast of interest (listen > baseline). Values are rounded to five decimal places. Only channels retained following the scalp coupling index (SCI) criterion are shown.

Table S24

Single-Subject GLM Results for HbO and HbR During the Listen Task (Participant pp16)

Channel	HbO Beta	HbO t	HbO p	HbR Beta	HbR t	HbR p
S8-D16	-0.77207	-2.17483	0.02971	-0.36685	-1.32051	0.18674
S13-D14	-0.38435	-0.98932	0.32257	-0.13055	-0.54413	0.58638
S16-D19	-0.30129	-0.79121	0.42887	-0.57293	-1.96736	0.04922

Note. Beta denotes the GLM regression coefficient. t and p values correspond to the contrast of interest (listen > baseline). Values are rounded to five decimal places. Only channels retained following the scalp coupling index (SCI) criterion are shown.

Table S25

Single-Subject GLM Results for HbO and HbR During the Listen Task (Participant pp17)

Channel	HbO Beta	HbO t	HbO p	HbR Beta	HbR t	HbR p
S3-D5	-0.14772	-0.53170	0.59497	0.35802	1.26839	0.20473
S5-D5	0.01388	0.04210	0.96642	0.15347	0.86193	0.38878
S5-D7	0.14026	0.60816	0.54312	-0.03178	-0.27432	0.78385
S7-D7	-0.15198	-0.72387	0.46919	-0.08599	-0.63666	0.52438

S7-D16	-0.05487	-0.27180	0.78579	0.27189	1.10662	0.26852
S11-D12	-0.22331	-0.78621	0.43179	0.10346	0.34074	0.73332
S13-D12	-0.36453	-1.16149	0.24551	0.27029	0.94789	0.34324
S13-D14	0.23791	0.85984	0.38993	-0.53699	-2.97294	0.00297
S15-D14	-0.24716	-1.14835	0.25089	-0.43959	-2.82504	0.00475

Note. Beta denotes the GLM regression coefficient. t and p values correspond to the contrast of interest (listen > baseline). Values are rounded to five decimal places. Only channels retained following the scalp coupling index (SCI) criterion are shown.

Table S26

Single-Subject GLM Results for HbO and HbR During the Listen Task (Participant pp18)

Channel	HbO Beta	HbO t	HbO p	HbR Beta	HbR t	HbR p
S3-D5	-1.60867	-4.02469	0.00006	0.08149	0.21174	0.83232
S5-D5	-0.54440	-1.39646	0.16265	0.64750	1.72478	0.08464
S5-D7	-1.01674	-2.59082	0.00961	-0.33492	-1.15886	0.24658
S7-D5	-0.95101	-2.74885	0.00601	0.45552	1.40709	0.15948
S7-D7	-1.51984	-4.33088	0.00002	1.32828	3.81990	0.00014
S8-D16	-0.34820	-0.95541	0.33943	-0.09298	-0.30317	0.76177
S11-D12	-0.79798	-2.13299	0.03299	0.56214	1.50896	0.13139
S13-D12	-0.38460	-1.00950	0.31279	0.98680	2.91026	0.00363
S13-D14	-0.56585	-1.57839	0.11455	-0.48010	-1.64295	0.10047
S15-D12	-0.85362	-2.02060	0.04339	-0.11416	-0.28652	0.77450
S15-D14	0.48888	1.32920	0.18386	-2.27767	-7.98290	0.00000
S16-D19	0.57663	1.54860	0.12156	0.84945	2.90915	0.00364

Note. Beta denotes the GLM regression coefficient. t and p values correspond to the contrast of interest (listen > baseline). Values are rounded to five decimal places. Only channels retained following the scalp coupling index (SCI) criterion are shown.

Table S27

Single-Subject GLM Results for HbO and HbR During the Listen Task (Participant pp19)

Channel	HbO Beta	HbO t	HbO p	HbR Beta	HbR t	HbR p
S3-D5	-0.10380	-0.29061	0.77136	0.08444	0.19818	0.84291

S5-D5	0.44464	1.50327	0.13286	-0.42303	-1.80364	0.07137
S5-D7	0.20014	0.96522	0.33450	-0.23837	-1.29664	0.19484
S7-D5	0.13351	0.46125	0.64465	-0.34657	-1.23380	0.21736
S7-D7	0.29849	1.40867	0.15902	0.22181	1.62592	0.10406
S7-D16	0.68881	2.38263	0.01724	-0.33002	-1.27548	0.20222
S8-D16	0.02801	0.08055	0.93580	0.13352	0.42223	0.67288
S11-D12	0.46258	0.92756	0.35370	-0.65127	-1.31304	0.18926
S13-D12	1.38923	3.01614	0.00258	-1.47455	-3.53373	0.00042
S13-D14	1.50513	5.34349	0.00000	-1.73287	-5.23961	0.00000
S15-D12	0.09532	0.23776	0.81208	-0.35557	-1.02577	0.30507
S15-D14	0.08247	0.28485	0.77578	0.12354	0.60540	0.54495
S15-D19	1.09073	2.67896	0.00742	-1.37341	-3.38118	0.00073
S16-D19	0.51120	1.69422	0.09031	-0.94102	-3.64314	0.00027

Note. Beta denotes the GLM regression coefficient. t and p values correspond to the contrast of interest (listen > baseline). Values are rounded to five decimal places. Only channels retained following the scalp coupling index (SCI) criterion are shown.

Table S28

Single-Subject GLM Results for HbO and HbR During the Listen Task (Participant pp20)

Channel	HbO Beta	HbO t	HbO p	HbR Beta	HbR t	HbR p
S3-D5	0.13457	0.29908	0.76490	-0.29728	-0.84597	0.39763
S5-D5	1.20443	3.95523	0.00008	-0.84046	-2.22134	0.02639
S5-D7	1.07098	3.18462	0.00146	0.48194	1.11866	0.26336
S7-D5	0.46311	1.51342	0.13026	-0.82774	-2.72006	0.00656
S7-D7	0.26875	0.90684	0.36455	-0.22634	-0.89357	0.37161
S7-D16	-0.73646	-2.20786	0.02732	0.60768	1.42453	0.15439
S8-D16	0.50058	1.86516	0.06224	0.50918	1.89767	0.05782
S11-D12	0.19973	0.65504	0.51248	0.41321	0.94631	0.34406
S13-D12	0.73721	1.75790	0.07885	0.31414	0.67828	0.49764
S13-D14	1.01366	2.78650	0.00536	-1.63130	-3.87220	0.00011
S15-D12	0.15843	0.42182	0.67318	0.81742	1.96453	0.04955
S15-D14	-0.17936	-0.37937	0.70444	-0.13215	-0.29830	0.76549

S15-D19	0.28287	0.74450	0.45662	0.33067	0.80523	0.42074
S16-D19	0.20989	0.50657	0.61249	0.38720	1.37011	0.17074

Note. Beta denotes the GLM regression coefficient. t and p values correspond to the contrast of interest (listen > baseline). Values are rounded to five decimal places. Only channels retained following the scalp coupling index (SCI) criterion are shown.

Table S29

Single-Subject GLM Results for HbO and HbR During the Listen Task (Participant pp21)

Channel	HbO Beta	HbO t	HbO p	HbR Beta	HbR t	HbR p
S3-D5	0.89201	2.16945	0.03011	-0.67377	-1.45150	0.14673
S7-D5	-0.20168	-0.55900	0.57620	0.47659	1.78501	0.07434
S7-D16	-0.61568	-2.16767	0.03025	0.63367	2.01948	0.04351
S11-D12	0.16571	0.69236	0.48876	0.24057	0.76673	0.44329
S15-D12	0.32767	1.18634	0.23556	-0.10459	-0.33252	0.73951

Note. Beta denotes the GLM regression coefficient. t and p values correspond to the contrast of interest (listen > baseline). Values are rounded to five decimal places. Only channels retained following the scalp coupling index (SCI) criterion are shown.

Table S30

Single-Subject GLM Results for HbO and HbR During the Listen Task (Participant pp22)

Channel	HbO Beta	HbO t	HbO p	HbR Beta	HbR t	HbR p
S3-D5	0.80585	2.40312	0.01630	-0.38399	-1.31748	0.18776
S5-D5	0.11825	0.55652	0.57789	-0.04710	-0.21788	0.82753
S5-D7	-0.13420	-0.55789	0.57695	-0.19140	-0.85158	0.39450
S7-D5	0.01079	0.03738	0.97018	0.46754	2.37600	0.01755
S7-D16	0.40980	1.38608	0.16580	-0.10720	-0.58013	0.56186
S11-D12	0.59384	1.97425	0.04843	-0.34947	-1.21432	0.22470
S13-D12	0.50477	2.25918	0.02393	-0.77908	-4.89918	0.00000
S13-D14	0.21956	0.92646	0.35427	-0.57174	-4.04540	0.00005
S15-D12	0.46320	1.46262	0.14366	0.06821	0.24486	0.80658
S15-D14	0.09921	0.41619	0.67730	-0.07850	-0.44527	0.65615
S15-D19	-0.01652	-0.07132	0.94315	-0.28628	-1.66260	0.09648

Note. Beta denotes the GLM regression coefficient. t and p values correspond to the contrast of interest (listen > baseline). Values are rounded to five decimal places. Only channels retained following the scalp coupling index (SCI) criterion are shown.

Table S31

Single-Subject GLM Results for HbO and HbR During the Listen Task (Participant pp23)

Channel	HbO Beta	HbO t	HbO p	HbR Beta	HbR t	HbR p
S3-D5	0.21221	0.80452	0.42115	0.45241	1.28891	0.19751
S7-D5	0.64479	2.35475	0.01859	0.33649	1.01511	0.31012
S7-D7	0.54820	1.76049	0.07841	0.45831	1.29905	0.19401
S7-D16	0.59095	2.16512	0.03044	-0.61498	-1.87674	0.06063
S11-D12	1.24299	3.35337	0.00081	0.22667	0.58597	0.55793
S13-D12	0.94771	2.87163	0.00411	-1.04237	-3.32043	0.00091
S13-D14	0.60756	1.59627	0.11051	-0.37249	-1.05386	0.29202
S15-D12	0.16426	0.55662	0.57782	-0.76171	-2.12216	0.03389
S15-D14	1.15839	2.72085	0.00654	1.28606	3.42198	0.00063
S15-D19	1.08398	2.74073	0.00616	-0.58721	-1.64586	0.09988

Note. Beta denotes the GLM regression coefficient. t and p values correspond to the contrast of interest (listen > baseline). Values are rounded to five decimal places. Only channels retained following the scalp coupling index (SCI) criterion are shown.

Table S32

Single-Subject GLM Results for HbO and HbR During the Listen Task (Participant pp24)

Channel	HbO Beta	HbO t	HbO p	HbR Beta	HbR t	HbR p
S3-D5	0.05986	0.15845	0.87411	0.16457	0.48775	0.62576
S5-D7	0.05527	0.17130	0.86400	-0.14745	-0.60249	0.54689
S7-D5	0.04170	0.14015	0.88855	-0.43370	-1.03566	0.30043
S7-D7	-0.52471	-2.15674	0.03109	0.11063	0.39539	0.69258
S7-D16	-0.60051	-1.87523	0.06084	0.59715	1.47167	0.14120
S8-D16	-0.83147	-2.87352	0.00408	0.62150	1.57546	0.11524
S11-D12	-0.43176	-1.21571	0.22418	0.42146	1.20074	0.22994
S13-D12	0.36119	0.87665	0.38074	-0.61209	-1.91417	0.05568

S13-D14	0.38844	1.39476	0.16318	-1.00523	-3.21384	0.00132
S15-D12	-0.38433	-1.05980	0.28931	0.03597	0.09662	0.92304
S15-D14	-0.62398	-2.34131	0.01927	0.34121	1.21621	0.22399
S15-D19	-0.36928	-0.83874	0.40167	0.17432	0.47250	0.63660
S16-D19	-0.44176	-1.56846	0.11687	0.33183	0.87850	0.37973

Note. Beta denotes the GLM regression coefficient. t and p values correspond to the contrast of interest (listen > baseline). Values are rounded to five decimal places. Only channels retained following the scalp coupling index (SCI) criterion are shown.

Table S33

Single-Subject GLM Results for HbO and HbR During the Listen Task (Participant pp25)

Channel	HbO Beta	HbO t	HbO p	HbR Beta	HbR t	HbR p
S3-D5	0.16693	0.49269	0.62226	-0.00771	-0.02085	0.98337
S5-D5	0.80185	2.69789	0.00701	-0.09932	-0.32371	0.74617
S5-D7	0.56877	2.05693	0.03976	-0.06899	-0.23245	0.81620
S7-D5	0.35603	0.96354	0.33533	0.17550	0.51132	0.60915
S7-D7	0.36553	1.13271	0.25740	-0.17351	-0.57579	0.56479
S7-D16	0.18736	0.50118	0.61627	0.01925	0.05309	0.95766
S8-D16	0.65038	2.26759	0.02341	0.22189	0.67247	0.50132
S11-D12	1.63457	4.62712	0.00000	-1.68835	-4.86534	0.00000
S13-D12	1.91654	6.05948	0.00000	-2.08493	-7.22697	0.00000
S13-D14	1.22215	3.50282	0.00047	0.35579	1.29591	0.19509
S15-D12	0.35131	1.13522	0.25635	0.24579	0.77153	0.44044
S15-D14	0.30259	1.21211	0.22555	0.62228	2.17240	0.02989
S15-D19	0.42278	1.36780	0.17145	-0.14224	-0.46569	0.64146
S16-D19	1.18822	3.43501	0.00060	-0.60797	-2.01128	0.04437

Note. Beta denotes the GLM regression coefficient. t and p values correspond to the contrast of interest (listen > baseline). Values are rounded to five decimal places. Only channels retained following the scalp coupling index (SCI) criterion are shown.

Table S34

Single-Subject GLM Results for HbO and HbR During the Listen Task (Participant pp26)

Channel	HbO Beta	HbO t	HbO p	HbR Beta	HbR t	HbR p
S3-D5	1.18766	3.36239	0.00078	-1.32339	-3.81586	0.00014
S5-D5	1.14854	3.30506	0.00096	-2.12989	-5.59300	0.00000
S5-D7	0.29002	0.93661	0.34901	-0.67208	-1.86453	0.06232
S7-D7	0.45928	2.09366	0.03635	-0.07692	-0.24675	0.80511
S8-D16	0.54732	1.93094	0.05356	-0.38480	-1.27431	0.20263
S11-D12	0.78728	2.21064	0.02712	-0.10252	-0.27278	0.78504
S13-D12	2.16474	6.66039	0.00000	-3.17511	-11.10720	0.00000
S13-D14	1.32325	3.49209	0.00048	-2.16419	-6.12475	0.00000
S15-D12	0.86354	2.54118	0.01108	-0.58590	-1.74763	0.08060
S15-D14	0.77447	2.32849	0.01993	-0.98207	-3.09845	0.00196
S15-D19	0.42170	1.29661	0.19484	0.12233	0.35405	0.72332
S16-D19	0.33148	1.12826	0.25928	-0.23746	-0.61769	0.53681

Note. Beta denotes the GLM regression coefficient. t and p values correspond to the contrast of interest (listen > baseline). Values are rounded to five decimal places. Only channels retained following the scalp coupling index (SCI) criterion are shown.

Table S35

Single-Subject GLM Results for HbO and HbR During the Listen Task (Participant pp27)

Channel	HbO Beta	HbO t	HbO p	HbR Beta	HbR t	HbR p
S3-D5	0.10748	0.31844	0.75017	0.10033	0.30022	0.76403
S5-D5	0.23046	0.97068	0.33178	-0.11014	-0.37197	0.70994
S5-D7	0.55908	2.94423	0.00326	-0.68316	-3.34141	0.00084
S7-D5	0.15869	0.52052	0.60273	0.04031	0.15290	0.87849
S7-D7	-0.25227	-0.99202	0.32126	0.25567	0.93645	0.34910
S7-D16	-0.14655	-0.70682	0.47973	0.29883	0.99957	0.31759
S8-D16	-0.18226	-0.87833	0.37983	0.33204	1.10038	0.27124
S11-D12	0.24004	0.76859	0.44219	-0.36655	-1.04287	0.29708
S13-D12	0.33305	1.29131	0.19668	-1.04210	-4.14320	0.00004
S13-D14	0.80409	3.37579	0.00074	-1.63267	-5.14951	0.00000
S15-D12	0.22409	0.90796	0.36396	-0.52292	-1.96283	0.04975
S15-D14	0.11555	0.41332	0.67939	-0.33937	-1.64811	0.09942

S15-D19	-0.26367	-0.98502	0.32468	-0.01947	-0.08999	0.92830
S16-D19	0.11908	0.59489	0.55195	-0.17423	-0.69575	0.48663

Note. Beta denotes the GLM regression coefficient. t and p values correspond to the contrast of interest (listen > baseline). Values are rounded to five decimal places. Only channels retained following the scalp coupling index (SCI) criterion are shown.

Table S35

Single-Subject GLM Results for HbO and HbR During the Speak Task (Participant pp1)

Channel	HbO Beta	HbO t	HbO p	HbR Beta	HbR t	HbR p
S2-D4	0.46373	2.16947	0.03011	0.14852	0.79046	0.42931
S3-D3	0.49525	2.32511	0.02012	-0.02199	-0.17405	0.86184
S3-D5	0.48942	2.31001	0.02094	-0.74106	-5.09142	0.00000
S4-D3	0.33583	1.28367	0.19934	0.18924	1.40438	0.16029
S4-D4	0.18446	0.69830	0.48503	-0.31911	-1.62592	0.10405
S4-D5	0.77236	3.78244	0.00016	-0.37566	-2.45973	0.01395
S4-D6	0.40351	1.73244	0.08328	-0.05482	-0.39680	0.69154
S5-D5	0.51333	2.28842	0.02217	-0.86511	-5.68672	0.00000
S5-D7	0.31115	1.43794	0.15054	0.23494	1.42265	0.15492
S6-D6	0.04486	0.17295	0.86270	-0.14826	-1.06196	0.28832
S6-D15	0.61837	2.71381	0.00668	-0.05719	-0.37732	0.70596
S6-D17	0.27801	1.02714	0.30442	-0.34423	-2.15703	0.03107
S7-D5	0.70107	3.28386	0.00103	-1.17964	-9.08549	0.00000
S7-D7	-0.08836	-0.37214	0.70981	-0.64702	-3.85925	0.00012
S7-D16	0.06373	0.23083	0.81746	-0.17633	-0.86245	0.38850
S8-D16	-0.05424	-0.23251	0.81615	-0.22685	-1.91569	0.05548
S10-D11	0.87235	4.02714	0.00006	-0.04615	-0.37066	0.71092
S11-D10	0.69257	3.00162	0.00270	-0.04229	-0.35582	0.72200
S11-D12	0.53648	2.50515	0.01228	-0.34633	-3.59494	0.00033
S12-D10	0.75662	3.16243	0.00158	-0.22569	-1.99303	0.04633
S12-D12	0.73402	3.38532	0.00072	-0.47487	-4.13834	0.00004
S12-D13	0.78704	3.26369	0.00111	-0.01744	-0.14747	0.88277
S13-D12	0.52527	2.06409	0.03908	-0.89170	-5.11367	0.00000

Channel	HbO Beta	HbO t	HbO p	HbR Beta	HbR t	HbR p
S13-D14	0.25216	1.27366	0.20287	-0.14418	-1.03336	0.30150
S14-D15	0.54345	2.25813	0.02400	-0.11869	-0.90232	0.36695
S14-D18	0.33059	1.57023	0.11645	-0.04331	-0.28941	0.77229
S15-D12	0.37099	1.65046	0.09894	-0.57827	-4.61260	0.00000
S15-D14	0.37253	1.63131	0.10291	-0.43826	-2.73667	0.00624
S16-D19	0.81583	3.67955	0.00024	-0.16935	-1.06455	0.28715
S17-D4	0.65346	2.32359	0.02020	-0.49951	-1.93773	0.05273
S17-D11	0.98838	4.01227	0.00006	0.07785	0.56639	0.57116
S18-D6	0.15593	0.52962	0.59641	-0.16237	-0.94384	0.34531
S20-D13	0.44765	1.83342	0.06682	0.12231	0.85335	0.39352

Note. Beta denotes the GLM regression coefficient. t and p values correspond to the contrast of interest (listen > baseline). Values are rounded to five decimal places. Only channels retained following the scalp coupling index (SCI) criterion are shown.

Table S36

Single-Subject GLM Results for HbO and HbR During the Speak Task (Participant pp2)

Channel	HbO Beta	HbO t	HbO p	HbR Beta	HbR t	HbR p
S3-D3	-0.55290	-2.57520	0.01006	0.13091	0.78462	0.43273
S3-D5	0.00612	0.02757	0.97800	-0.15912	-0.79978	0.42389
S4-D5	-0.57384	-2.46831	0.01362	0.19931	1.14222	0.25344
S5-D5	0.01353	0.07015	0.94408	-0.06955	-0.41548	0.67781
S6-D4	-0.66236	-3.75692	0.00017	-0.49632	-2.45203	0.01425
S7-D5	-0.25912	-1.08249	0.27911	-0.57449	-3.56133	0.00037
S7-D7	-0.37087	-1.96228	0.04981	-0.14834	-0.82415	0.40991
S7-D16	-0.38834	-1.90461	0.05691	-0.00327	-0.01955	0.98441
S8-D16	-0.15746	-0.70436	0.48125	-0.08651	-0.50454	0.61391
S10-D11	-0.72262	-2.86519	0.00419	-0.15125	-0.77523	0.43826
S11-D10	-0.26320	-1.21181	0.22567	0.49847	2.42163	0.01550
S11-D12	-0.10223	-0.45314	0.65047	0.04118	0.21849	0.82706
S12-D10	-0.06583	-0.29742	0.76617	0.01662	0.08614	0.93136
S12-D11	-0.74909	-3.35585	0.00080	-0.45486	-2.59851	0.00940

Channel	HbO Beta	HbO t	HbO p	HbR Beta	HbR t	HbR p
S12-D12	-0.36526	-1.68506	0.09206	-0.16232	-0.84788	0.39656
S12-D13	-0.26493	-1.21926	0.22283	0.00351	0.01710	0.98636
S13-D12	-0.35432	-1.49019	0.13626	-0.36982	-2.01557	0.04392
S13-D14	-0.15822	-0.94639	0.34402	0.14736	1.00875	0.31316
S14-D11	-1.19882	-4.31707	0.00002	0.31060	1.59445	0.11092
S14-D13	-0.60160	-2.80133	0.00512	0.40730	2.07465	0.03809
S14-D15	-1.25366	-5.84733	0.00000	0.06402	0.36024	0.71869
S14-D18	-0.81745	-3.47161	0.00052	0.70950	3.43935	0.00059
S15-D12	-0.51540	-2.19777	0.02803	0.10709	0.52226	0.60152
S15-D13	-0.71314	-3.05942	0.00223	0.33486	1.53971	0.12372
S15-D14	-0.39309	-2.12687	0.03350	0.42801	2.69415	0.00709
S15-D19	-0.48908	-2.05028	0.04041	0.56597	2.60575	0.00920
S16-D19	0.36991	1.77856	0.07540	0.30256	1.57310	0.11578
S17-D4	-1.46524	-6.00113	0.00000	0.52339	2.76143	0.00578
S17-D11	-0.97385	-4.38490	0.00001	-0.11557	-0.68001	0.49654
S17-D15	-1.33378	-5.55920	0.00000	-0.19345	-1.00352	0.31568
S20-D13	-1.14251	-5.25673	0.00000	0.82978	3.79850	0.00015

Note. Beta denotes the GLM regression coefficient. t and p values correspond to the contrast of interest (listen > baseline). Values are rounded to five decimal places. Only channels retained following the scalp coupling index (SCI) criterion are shown.

Table S37

Single-Subject GLM Results for HbO and HbR During the Speak Task (Participant pp3)

Channel	HbO Beta	HbO t	HbO p	HbR Beta	HbR t	HbR p
S2-D4	0.49563	2.42412	0.01539	0.09222	0.72468	0.46870
S3-D3	0.61139	2.40798	0.01609	0.12955	1.45925	0.14458
S3-D5	1.01656	4.88246	0.00000	0.21234	2.09738	0.03603
S4-D4	0.85208	3.41962	0.00063	0.14151	1.52535	0.12726
S4-D5	0.58681	2.99939	0.00272	0.06402	1.10713	0.26831
S4-D6	0.94740	5.20134	0.00000	0.20139	2.14440	0.03207
S5-D5	0.44804	2.43906	0.01477	-0.02140	-0.14890	0.88164

Channel	HbO Beta	HbO t	HbO p	HbR Beta	HbR t	HbR p
S5-D7	0.79651	4.19340	0.00003	-0.04947	-0.30045	0.76385
S6-D4	0.20969	0.99922	0.31775	0.12002	0.97381	0.33021
S6-D6	0.11304	0.59006	0.55519	0.05259	0.96597	0.33412
S6-D15	0.49546	2.50413	0.01232	0.15931	2.00246	0.04531
S6-D17	0.37751	1.67300	0.09441	0.12938	0.86082	0.38939
S7-D5	0.77611	3.70180	0.00022	0.07313	0.80956	0.41825
S7-D6	0.90272	4.34954	0.00001	0.02071	0.29072	0.77128
S7-D7	-0.04509	-0.25925	0.79546	-0.09167	-0.71118	0.47702
S7-D16	-0.12054	-0.68603	0.49274	-0.10076	-0.77630	0.43762
S8-D26	-0.59672	-2.93076	0.00340	0.03177	-0.13152	0.89537
S11-D9	0.40738	1.50209	0.13316	0.07916	1.13309	0.25725
S11-D12	1.02567	3.98460	0.00007	0.14206	1.95118	0.05111
S12-D10	0.84316	4.07914	0.00005	0.21445	1.46096	0.14411
S12-D11	0.59644	2.37835	0.01744	0.12995	0.87265	0.38291
S12-D12	0.71626	2.74270	0.00612	0.24766	2.19069	0.02854
S12-D13	0.69115	3.26452	0.00111	0.17241	1.20854	0.22692
S13-D12	0.66230	3.13163	0.00175	0.07029	0.32108	0.74817
S13-D14	-0.35912	-2.05834	0.03963	0.05695	1.20890	0.22678
S13-D27	-0.71678	-2.89299	0.00384	-0.11172	-0.14079	0.88804
S14-D13	0.06344	0.27464	0.78361	0.12477	1.15246	0.24921
S14-D15	-0.51077	-2.24225	0.02500	0.20594	2.69195	0.00714
S14-D18	-0.42977	-1.91632	0.05540	0.12613	1.36832	0.17129
S15-D13	0.63366	2.90466	0.00370	0.14339	1.79215	0.07319
S15-D14	-0.10226	-0.54334	0.58693	-0.06347	-0.32304	0.74668
S15-D19	0.43064	2.14992	0.03163	-0.04628	-0.16444	0.86939
S15-D29	-0.25854	-1.04010	0.29836	-0.03303	-0.38184	0.70260
S17-D4	0.29406	1.28324	0.19949	0.13436	1.37723	0.16853
S17-D11	0.77199	3.26323	0.00111	0.16765	1.33008	0.18357
S17-D15	0.30502	2.24909	0.02457	0.21970	1.76779	0.07718
S18-D6	0.52631	3.04360	0.00235	0.02807	0.25455	0.79908

Channel	HbO Beta	HbO t	HbO p	HbR Beta	HbR t	HbR p
S18-D16	0.64979	3.30365	0.00096	0.11364	0.49403	0.62132
S20-D18	0.23757	1.27436	0.20262	0.07342	0.40277	0.68714

Note. Beta denotes the GLM regression coefficient. t and p values correspond to the contrast of interest (listen > baseline). Values are rounded to five decimal places. Only channels retained following the scalp coupling index (SCI) criterion are shown.

Table S38

Single-Subject GLM Results for HbO and HbR During the Speak Task (Participant pp4)

Channel	HbO Beta	HbO t	HbO p	HbR Beta	HbR t	HbR p
S3-D5	-0.24997	-1.02125	0.30721	0.07032	0.43494	0.66363
S4-D5	0.24318	1.01463	0.31035	-0.48472	-2.02754	0.04268
S5-D5	-0.19191	-0.85561	0.39227	0.27833	1.80745	0.07078
S5-D7	0.25003	1.35380	0.17589	0.09036	0.56336	0.57323
S7-D5	0.19666	0.89929	0.36856	-0.26731	-1.39870	0.16199
S7-D6	0.46086	1.97344	0.04852	-0.34171	-1.58811	0.11235
S7-D7	0.38600	1.50512	0.13238	0.21293	1.37185	0.17020
S7-D16	-0.03056	-0.14955	0.88113	-0.13952	-1.14092	0.25398
S8-D16	0.36368	1.99181	0.04647	-0.28826	-1.50420	0.13262
S11-D10	0.08132	0.30954	0.75693	-0.25586	-1.03453	0.30096
S11-D12	-0.04695	-0.19896	0.84230	-0.07897	-0.36537	0.71486
S12-D12	0.41454	1.49665	0.13457	-0.66613	-2.62828	0.00862
S12-D13	0.79768	3.39193	0.00070	-0.46876	-2.23611	0.02541
S13-D12	0.00132	0.00588	0.99531	-0.20287	-1.08178	0.27942
S13-D14	-0.35622	-1.93427	0.05316	0.01196	0.06342	0.94944
S15-D12	-0.10235	-0.44395	0.65711	-0.16276	-0.80100	0.42319
S15-D13	0.31490	1.34357	0.17917	-0.55627	-2.80250	0.00510
S15-D14	0.09472	0.43471	0.66380	0.04400	0.32276	0.74689
S15-D19	0.08861	0.39922	0.68975	-0.18452	-1.14079	0.25403
S16-D19	-0.04296	-0.17780	0.85889	-0.24418	-1.19650	0.23158
S20-D13	0.77297	3.21215	0.00133	-0.44154	-1.87548	0.06081

Note. Beta denotes the GLM regression coefficient. t and p values correspond to the contrast of interest (listen > baseline). Values are rounded to five decimal places. Only channels retained following the scalp coupling index (SCI) criterion are shown.

Table S39

Single-Subject GLM Results for HbO and HbR During the Speak Task (Participant pp5)

Channel	HbO Beta	HbO t	HbO p	HbR Beta	HbR t	HbR p
S2-D4	-0.22621	-0.77757	0.43687	-0.71233	-3.89562	0.00010
S3-D3	-0.05679	-0.21250	0.83173	0.57147	2.24940	0.02455
S3-D5	-0.27505	-1.09866	0.27199	-0.42101	-1.89299	0.05844
S4-D3	0.84639	2.94192	0.00328	-0.14260	-0.58881	0.55602
S4-D4	0.06237	0.25144	0.80148	-0.37966	-2.41879	0.01562
S4-D5	1.12481	4.20915	0.00003	-0.09997	-0.39585	0.69224
S4-D6	0.65656	2.23238	0.02565	-0.26060	-1.32195	0.18627
S5-D5	-0.32971	-1.52899	0.12635	-0.38008	-2.26160	0.02378
S5-D7	-0.03600	-0.20622	0.83663	-0.51631	-2.95646	0.00313
S6-D4	-0.16217	-0.61918	0.53584	-0.83732	-5.60644	0.00000
S6-D6	0.00233	0.00872	0.99305	-0.57767	-4.02663	0.00006
S6-D15	0.33555	1.16161	0.24547	-0.54150	-3.50341	0.00046
S7-D5	0.34132	1.31241	0.18946	0.04795	0.18187	0.85569
S7-D6	0.27899	1.08768	0.27681	-0.95175	-3.98294	0.00007
S7-D7	-0.21243	-1.19553	0.23196	-0.81516	-5.23813	0.00000
S7-D16	0.36145	1.56009	0.11882	-0.38017	-1.79125	0.07334
S8-D16	0.93400	4.06519	0.00005	0.23095	1.35188	0.17650
S10-D11	0.62536	2.06032	0.03944	-0.51762	-2.92450	0.00347
S11-D10	0.26000	0.91647	0.35948	0.55535	2.11128	0.03482
S11-D12	0.48921	1.68530	0.09202	-0.72025	-2.79287	0.00525
S12-D10	0.73903	2.48488	0.01300	-0.37425	-1.59649	0.11047
S12-D11	0.76739	2.48231	0.01310	-0.74956	-4.41523	0.00001
S12-D12	0.65567	2.30214	0.02138	-0.44771	-2.51989	0.01178
S12-D13	1.22636	5.55238	0.00000	-0.28579	-1.56419	0.11786
S13-D12	-0.18280	-0.73406	0.46296	-1.31072	-4.98311	0.00000

Channel	HbO Beta	HbO t	HbO p	HbR Beta	HbR t	HbR p
S13-D14	-0.70130	-3.73700	0.00019	-0.49924	-3.30123	0.00097
S14-D11	-0.32407	-1.23191	0.21806	0.50530	2.27531	0.02294
S14-D13	-0.13828	-0.56662	0.57101	-0.37525	-2.17654	0.02958
S14-D15	0.38049	1.60058	0.10956	-0.74379	-3.80580	0.00014
S14-D18	0.33740	1.23142	0.21824	-0.28694	-1.59228	0.11141
S15-D12	-0.24119	-0.81649	0.41428	-0.87912	-3.51881	0.00044
S15-D13	-0.22657	-0.85294	0.39375	-0.18162	-1.00111	0.31684
S15-D14	-1.02470	-4.39838	0.00001	-0.00240	-0.01039	0.99171
S15-D19	-0.45986	-1.87300	0.06115	-0.36257	-1.60359	0.10889
S16-D19	0.87383	4.42242	0.00001	-0.59196	-2.78762	0.00534
S17-D4	0.32111	1.06349	0.28763	-0.13646	-0.87007	0.38432
S17-D11	0.76635	2.57926	0.00994	-0.12180	-0.61868	0.53617
S17-D15	0.79427	2.80720	0.00502	-0.24290	-1.14525	0.25218
S18-D6	-0.61510	-3.37945	0.00073	-0.11725	-0.71561	0.47428
S20-D13	-0.03204	-0.13221	0.89482	0.00591	0.03356	0.97323

Note. Beta denotes the GLM regression coefficient. t and p values correspond to the contrast of interest (listen > baseline). Values are rounded to five decimal places. Only channels retained following the scalp coupling index (SCI) criterion are shown.

Table S40

Single-Subject GLM Results for HbO and HbR During the Speak Task (Participant pp6)

Channel	HbO Beta	HbO t	HbO p	HbR Beta	HbR t	HbR p
S5-D5	0.09415	0.54350	0.58682	0.20512	1.30305	0.19265
S5-D7	-0.41801	-2.26013	0.02388	-0.05662	-0.35681	0.72125
S6-D6	-0.66191	-2.71335	0.00669	0.66924	2.81584	0.00489
S7-D7	0.05440	0.23883	0.81125	-0.57778	-2.68697	0.00725
S7-D16	0.47808	2.42673	0.01529	0.39473	2.02871	0.04257
S11-D12	0.68016	2.14877	0.03172	0.36320	1.86871	0.06175
S13-D14	0.00701	0.05584	0.95548	-0.15821	-1.46077	0.14417
S15-D14	-0.05339	-0.28565	0.77516	-0.31652	-1.71215	0.08696
S17-D11	-0.00127	-0.00538	0.99570	0.61060	2.68595	0.00727

Channel	HbO Beta	HbO t	HbO p	HbR Beta	HbR t	HbR p
S17-D15	-0.45165	-1.98443	0.04729	-0.01455	-0.05957	0.95250
S18-D6	-0.16470	-0.70802	0.47898	0.27905	1.07158	0.28398

Note. Beta denotes the GLM regression coefficient. t and p values correspond to the contrast of interest (listen > baseline). Values are rounded to five decimal places. Only channels retained following the scalp coupling index (SCI) criterion are shown.

Table S41

Single-Subject GLM Results for HbO and HbR During the Speak Task (Participant pp7)

Channel	HbO Beta	HbO t	HbO p	HbR Beta	HbR t	HbR p
S8-D16	0.26508	1.15010	0.25017	-0.03186	-0.12236	0.90262
S11-D10	0.08919	0.39505	0.69282	-0.07625	-0.58804	0.55654
S11-D12	0.36874	1.89255	0.05849	-0.00880	-0.04747	0.96214
S16-D19	0.37117	1.63250	0.10265	0.02276	0.08862	0.92939

Note. Beta denotes the GLM regression coefficient. t and p values correspond to the contrast of interest (listen > baseline). Values are rounded to five decimal places. Only channels retained following the scalp coupling index (SCI) criterion are shown.

Table S42

Single-Subject GLM Results for HbO and HbR During the Speak Task (Participant pp8)

Channel	HbO Beta	HbO t	HbO p	HbR Beta	HbR t	HbR p
S2-D4	0.60986	2.64201	0.00827	-0.17610	-0.77935	0.43582
S3-D3	0.79804	3.66353	0.00025	0.08714	0.47311	0.63616
S3-D5	1.09600	4.85945	0.00000	-0.08069	-0.33757	0.73571
S4-D3	0.87445	3.88349	0.00010	-0.68789	-2.71482	0.00666
S4-D4	0.38203	1.63455	0.10222	0.17629	0.72836	0.46644
S4-D5	1.39697	6.63184	0.00000	-1.24012	-5.64295	0.00000
S4-D6	0.78158	3.61343	0.00031	-0.78689	-3.18938	0.00144
S5-D5	1.79702	8.58833	0.00000	-1.15485	-4.68278	0.00000
S5-D7	-1.60000	-7.88884	0.00000	0.20435	0.92085	0.35719
S6-D4	0.86936	3.73123	0.00019	-0.46226	-1.93951	0.05251
S6-D15	0.95690	4.16117	0.00003	-0.78923	-3.51793	0.00044

Channel	HbO Beta	HbO t	HbO p	HbR Beta	HbR t	HbR p
S6-D17	0.68682	3.04253	0.00236	-0.06601	-0.26748	0.78912
S7-D5	1.23753	5.74179	0.00000	-0.25194	-1.14838	0.25088
S7-D6	0.76781	3.21291	0.00132	-0.63955	-2.29857	0.02158
S7-D7	2.35814	10.33251	0.00000	-2.76770	-11.29913	0.00000
S7-D16	1.50813	7.27488	0.00000	-0.87204	-3.61541	0.00030
S10-D11	0.40084	1.69695	0.08979	0.22140	0.97906	0.32761
S11-D10	-0.02402	-0.09764	0.92222	0.78016	3.39605	0.00069
S12-D10	0.60662	2.52146	0.01173	0.02572	0.11228	0.91061
S12-D11	0.57515	2.41062	0.01597	0.13176	0.57022	0.56856
S12-D13	1.22846	5.05442	0.00000	-0.93467	-3.93536	0.00008
S13-D14	1.24256	7.72042	0.00000	-1.08193	-5.48738	0.00000
S14-D11	0.45977	1.57732	0.11480	-0.22476	-1.14676	0.25155
S14-D13	1.16282	4.72385	0.00000	-1.02445	-4.43827	0.00001
S14-D15	0.56576	2.18333	0.02907	-0.19519	-0.92677	0.35410
S14-D18	0.58862	2.56536	0.01034	-0.17956	-0.84687	0.39712
S15-D13	1.71512	8.22438	0.00000	-2.54494	-11.77253	0.00000
S15-D14	1.22584	6.17049	0.00000	-2.16378	-8.96934	0.00000
S15-D19	1.38828	6.35031	0.00000	-1.58801	-7.27114	0.00000
S17-D4	0.76902	3.23923	0.00121	-1.03080	-5.03704	0.00000
S17-D11	0.24798	0.99943	0.31765	0.64572	3.39095	0.00070
S17-D15	0.67435	2.81967	0.00483	0.00357	0.01878	0.98502
S18-D6	0.87053	4.08357	0.00005	-0.10357	-0.42035	0.67425
S20-D13	1.11545	5.10166	0.00000	-1.02912	-4.82155	0.00000

Note. Beta denotes the GLM regression coefficient. t and p values correspond to the contrast of interest (listen > baseline). Values are rounded to five decimal places. Only channels retained following the scalp coupling index (SCI) criterion are shown.

Table S43

Single-Subject GLM Results for HbO and HbR During the Speak Task (Participant pp9)

Channel	HbO Beta	HbO t	HbO p	HbR Beta	HbR t	HbR p
S3-D5	-0.60968	-3.14387	0.00168	-0.34796	-2.00396	0.04514

Channel	HbO Beta	HbO t	HbO p	HbR Beta	HbR t	HbR p
S4-D5	0.65236	2.90395	0.00371	-0.80853	-3.74828	0.00018
S5-D5	-0.15083	-0.73891	0.46001	-0.26869	-1.48451	0.13776
S5-D7	0.11535	0.59577	0.55136	-0.60862	-2.67649	0.00747
S7-D5	-0.70188	-3.69921	0.00022	-0.06936	-0.32019	0.74884
S7-D7	-0.42755	-2.44822	0.01440	-0.01433	-0.11355	0.90960
S7-D16	-0.34225	-1.57663	0.11496	-0.11329	-0.53513	0.59259
S8-D16	-0.16017	-0.86978	0.38448	-0.15288	-0.89826	0.36910
S11-D10	0.03352	0.14054	0.88824	-0.29290	-1.20636	0.22775
S11-D12	-0.37534	-1.94164	0.05225	-0.24085	-1.23756	0.21595
S13-D12	-0.47655	-2.55207	0.01075	0.16147	1.22794	0.21954
S13-D14	-0.24445	-1.39422	0.16333	-0.15164	-1.10198	0.27054
S15-D12	-0.69194	-4.26625	0.00002	-0.01859	-0.10779	0.91417
S15-D14	-0.60514	-3.11877	0.00183	-0.24008	-1.36400	0.17265
S15-D19	-0.52000	-3.57255	0.00036	-0.40677	-2.45584	0.01410
S16-D19	-0.18245	-1.43136	0.15241	0.29040	2.10379	0.03546

Note. Beta denotes the GLM regression coefficient. t and p values correspond to the contrast of interest (listen > baseline). Values are rounded to five decimal places. Only channels retained following the scalp coupling index (SCI) criterion are shown.

Table S44

Single-Subject GLM Results for HbO and HbR During the Speak Task (Participant pp10)

Channel	HbO Beta	HbO t	HbO p	HbR Beta	HbR t	HbR p
S3-D5	0.05238	0.17685	0.85963	0.32791	1.14486	0.25234
S4-D3	0.14430	0.60364	0.54612	-0.04532	-0.21382	0.83070
S4-D6	0.57810	2.33947	0.01937	-0.75220	-3.38472	0.00072
S5-D5	0.43716	1.80001	0.07194	-0.46722	-1.71173	0.08703
S5-D7	-0.44921	-2.04369	0.04106	-0.29841	-1.42726	0.15359
S7-D5	1.49016	6.45200	0.00000	-1.32800	-5.01967	0.00000
S7-D6	1.29822	5.92941	0.00000	-1.87156	-9.02868	0.00000
S7-D7	0.60354	2.78461	0.00539	-1.05281	-4.37176	0.00001
S7-D16	0.70066	3.03542	0.00242	-0.36130	-1.70122	0.08899

Channel	HbO Beta	HbO t	HbO p	HbR Beta	HbR t	HbR p
S8-D16	0.60110	2.51316	0.01201	0.73482	3.69698	0.00022
S11-D10	-0.15703	-0.58015	0.56185	0.34896	1.43726	0.15073
S11-D12	-0.01938	-0.07785	0.93796	-0.72685	-2.61057	0.00908
S12-D10	-0.05011	-0.20111	0.84063	0.43192	1.94061	0.05238
S12-D12	0.12842	0.56129	0.57463	0.06745	0.27674	0.78199
S12-D13	0.66265	2.68206	0.00735	-0.56262	-1.90775	0.05650
S13-D12	0.32978	1.37620	0.16884	-0.61297	-3.05490	0.00227
S13-D14	-0.80222	-3.03103	0.00245	-0.92453	-4.80794	0.00000
S15-D12	0.35756	1.54981	0.12127	-1.11308	-4.73686	0.00000
S15-D13	0.65800	2.78665	0.00535	-1.39269	-6.12309	0.00000
S15-D14	-0.41393	-1.96643	0.04933	-0.52376	-2.63758	0.00839
S15-D19	-0.81098	-3.69882	0.00022	-0.33655	-1.57663	0.11497
S18-D6	0.32368	1.22424	0.22094	0.18469	0.65900	0.50994
S20-D13	0.39756	1.70478	0.08832	0.09182	0.40171	0.68792

Note. Beta denotes the GLM regression coefficient. t and p values correspond to the contrast of interest (listen > baseline). Values are rounded to five decimal places. Only channels retained following the scalp coupling index (SCI) criterion are shown.

Table S45

Single-Subject GLM Results for HbO and HbR During the Speak Task (Participant pp11)

Channel	HbO Beta	HbO t	HbO p	HbR Beta	HbR t	HbR p
S5-D7	-0.01584	-0.07694	0.93868	-0.21690	-1.04006	0.29838
S6-D15	-0.74799	-3.66817	0.00025	-1.07013	-4.34021	0.00001
S8-D16	0.31200	1.41446	0.15731	-0.28078	-1.34463	0.17882
S13-D14	0.03755	0.23677	0.81284	0.03170	0.17938	0.85765
S14-D15	-0.70325	-3.40445	0.00067	-1.20192	-5.23134	0.00000
S16-D19	-0.10028	-0.48696	0.62632	0.31416	1.67470	0.09407
S17-D4	-0.69593	-3.40370	0.00067	-1.00806	-4.73997	0.00000
S17-D11	-0.57455	-2.94411	0.00326	-1.11912	-4.98749	0.00000
S17-D15	-0.76361	-3.34974	0.00082	-0.95132	-4.74867	0.00000

Note. Beta denotes the GLM regression coefficient. t and p values correspond to the contrast of interest (listen > baseline). Values are rounded to five decimal places. Only channels retained following the scalp coupling index (SCI) criterion are shown.

Table S46

Single-Subject GLM Results for HbO and HbR During the Speak Task (Participant pp12)

Channel	HbO Beta	HbO t	HbO p	HbR Beta	HbR t	HbR p
S4-D5	0.02655	0.08618	0.93133	0.00708	0.02601	0.97925
S5-D5	0.39521	1.48824	0.13678	0.25996	1.32907	0.18391
S5-D7	0.48281	1.75063	0.08010	0.29870	1.62795	0.10363
S6-D15	-0.32060	-1.76311	0.07797	-0.56521	-1.98306	0.04744
S7-D5	0.71270	2.37039	0.01782	-0.04912	-0.27302	0.78485
S7-D7	0.59574	2.49297	0.01271	0.19281	1.24718	0.21242
S7-D16	0.73856	3.05233	0.00229	-0.40928	-2.70507	0.00686
S8-D16	0.45221	2.11349	0.03463	-0.24397	-1.64780	0.09949
S11-D10	-0.34811	-1.12364	0.26124	0.31400	1.20689	0.22756
S11-D12	-0.11695	-0.39420	0.69346	0.20987	0.80278	0.42216
S12-D10	0.17732	0.53913	0.58983	0.03426	0.12438	0.90102
S12-D12	0.57380	1.94812	0.05148	-0.38413	-1.63970	0.10116
S12-D13	0.58479	2.12076	0.03401	-0.16922	-0.68218	0.49517
S13-D12	-0.09480	-0.49071	0.62366	-0.56314	-3.27631	0.00106
S13-D14	0.44548	1.96149	0.04990	-0.46886	-2.85815	0.00429
S15-D12	0.57225	2.13168	0.03310	-0.23231	-1.09959	0.27159
S15-D13	1.01677	3.79803	0.00015	-1.48956	-6.11064	0.00000
S15-D14	0.46728	2.17364	0.02980	0.20454	1.12064	0.26252
S15-D19	0.79829	2.50828	0.01218	-0.47507	-1.63598	0.10193
S16-D19	0.80625	2.77800	0.00550	-0.30901	-1.14310	0.25308
S20-D13	0.23658	0.78363	0.43331	0.19804	0.67241	0.50137

Note. Beta denotes the GLM regression coefficient. t and p values correspond to the contrast of interest (listen > baseline). Values are rounded to five decimal places. Only channels retained following the scalp coupling index (SCI) criterion are shown.

Table S47

Single-Subject GLM Results for HbO and HbR During the Speak Task (Participant pp13)

Channel	HbO Beta	HbO t	HbO p	HbR Beta	HbR t	HbR p
S4-D3	0.41591	2.24186	0.02503	0.39886	2.41673	0.01571
S4-D5	1.45564	7.89392	0.00000	-0.99512	-7.04486	0.00000
S5-D5	0.45905	3.07876	0.00209	-0.02989	-0.17762	0.85903
S5-D7	-0.27181	-1.38495	0.16615	-0.06799	-0.54497	0.58581
S7-D5	1.65683	7.22680	0.00000	-0.87829	-5.12379	0.00000
S7-D6	0.27595	1.35782	0.17461	0.38480	1.97821	0.04798
S7-D7	-0.56185	-3.33504	0.00086	-0.18651	-1.08995	0.27581
S7-D16	1.30406	5.54855	0.00000	0.36706	1.78570	0.07423
S8-D16	-0.94295	-3.80685	0.00014	1.10151	4.97691	0.00000
S11-D12	0.25806	1.39262	0.16382	0.15111	1.19636	0.23163
S13-D12	-0.21916	-1.26262	0.20681	-0.24452	-1.18564	0.23584
S13-D14	0.21554	1.15416	0.24851	-0.35613	-2.42049	0.01555
S15-D12	0.28200	1.66082	0.09684	0.06624	0.67755	0.49810
S15-D13	-0.09115	-0.52848	0.59720	0.20377	1.63239	0.10269
S15-D14	0.02436	0.10561	0.91590	-0.26780	-1.42780	0.15344
S15-D19	0.11955	0.65932	0.50974	0.30059	2.09528	0.03622
S16-D19	-0.35065	-1.82142	0.06863	-0.24670	-1.15684	0.24742
S18-D6	0.26006	1.12231	0.26181	0.73621	3.26806	0.00109
S20-D13	-0.15391	-0.82512	0.40936	0.32638	2.23573	0.02543

Note. Beta denotes the GLM regression coefficient. t and p values correspond to the contrast of interest (listen > baseline). Values are rounded to five decimal places. Only channels retained following the scalp coupling index (SCI) criterion are shown.

Table S48

Single-Subject GLM Results for HbO and HbR During the Speak Task (Participant pp14)

Channel	HbO Beta	HbO t	HbO p	HbR Beta	HbR t	HbR p
S2-D4	0.33293	1.48220	0.13837	0.13207	0.53600	0.59199
S3-D3	-0.51045	-1.96528	0.04945	0.44426	1.99948	0.04562
S3-D5	1.14750	4.68938	0.00000	-1.51311	-6.40205	0.00000

Channel	HbO Beta	HbO t	HbO p	HbR Beta	HbR t	HbR p
S4-D3	-0.26651	-1.08616	0.27747	0.93736	3.81344	0.00014
S4-D4	-0.56374	-2.22983	0.02581	-0.07173	-0.27845	0.78068
S4-D5	1.54537	6.77926	0.00000	-0.87246	-3.59176	0.00033
S4-D6	0.04034	0.13917	0.88932	-1.63541	-8.21518	0.00000
S5-D5	2.52589	10.80296	0.00000	-1.79040	-7.08883	0.00000
S6-D4	0.09810	0.41804	0.67594	-0.76441	-2.92330	0.00348
S6-D17	-1.03318	-4.37585	0.00001	0.76111	2.96219	0.00307
S7-D5	1.72315	7.20777	0.00000	-2.73353	-12.74039	0.00000
S7-D6	1.37832	5.82424	0.00000	-2.30036	-10.37811	0.00000
S7-D16	0.90273	3.76834	0.00017	-1.38029	-5.94365	0.00000
S10-D11	1.84399	8.46748	0.00000	-0.16589	-0.70302	0.48208
S11-D10	0.21971	0.87816	0.37991	0.51874	2.01105	0.04439
S11-D12	1.32901	5.47315	0.00000	-1.43444	-6.43760	0.00000
S12-D10	1.66992	7.07595	0.00000	0.05973	0.28333	0.77694
S12-D11	0.61513	2.44377	0.01458	-0.05806	-0.27346	0.78451
S12-D12	1.74869	7.29327	0.00000	-0.59497	-2.62764	0.00863
S12-D13	1.14521	4.50026	0.00001	-0.83150	-3.57217	0.00036
S13-D12	-0.28596	-1.49891	0.13398	-0.36966	-1.99789	0.04580
S13-D14	0.15732	0.97470	0.32977	0.00925	0.04081	0.96745
S14-D11	0.09875	0.36005	0.71883	0.20885	0.79332	0.42764
S14-D13	0.55137	2.01509	0.04396	-0.44230	-1.73832	0.08223
S14-D15	0.61445	2.59484	0.00950	-2.40934	-11.24535	0.00000
S14-D18	-0.17839	-0.66874	0.50370	-0.19926	-0.75221	0.45197
S15-D13	0.43978	1.90669	0.05663	-0.32796	-1.41256	0.15786
S15-D14	0.19496	0.87052	0.38407	-0.33540	-1.48085	0.13873
S15-D19	-1.10524	-4.49602	0.00001	-0.16946	-0.63648	0.52450
S17-D4	-0.72102	-3.47746	0.00051	-1.10586	-4.66226	0.00000
S17-D11	0.50482	2.12512	0.03364	-2.08125	-10.20478	0.00000
S18-D6	-1.10049	-4.52129	0.00001	0.02895	0.11631	0.90742
S20-D13	1.03420	4.36242	0.00001	-1.06231	-4.54589	0.00001

Note. Beta denotes the GLM regression coefficient. t and p values correspond to the contrast of interest (listen > baseline). Values are rounded to five decimal places. Only channels retained following the scalp coupling index (SCI) criterion are shown.

Table S49

Single-Subject GLM Results for HbO and HbR During the Speak Task (Participant pp15)

Channel	HbO Beta	HbO t	HbO p	HbR Beta	HbR t	HbR p
S3-D5	0.05908	0.23600	0.81344	-0.10359	-0.57000	0.56871
S4-D3	-0.06239	-0.29669	0.76672	0.02006	0.12484	0.90066
S4-D4	-0.28360	-1.05789	0.29018	-0.40266	-2.75262	0.00594
S4-D5	-0.00123	-0.00565	0.99549	-0.04802	-0.28154	0.77831
S4-D6	0.19142	0.95961	0.33732	-0.27286	-2.46460	0.01376
S5-D5	0.77616	3.22374	0.00128	-0.83482	-4.67559	0.00000
S5-D7	0.45674	2.60288	0.00928	-0.61664	-4.02607	0.00006
S6-D15	0.25505	1.60650	0.10825	-0.29234	-1.44085	0.14972
S6-D17	-0.34818	-1.46662	0.14257	-0.36397	-2.22586	0.02609
S8-D16	-0.12162	-0.58565	0.55815	0.29934	1.70204	0.08884
S11-D10	0.15902	0.67364	0.50058	0.04332	0.27470	0.78356
S11-D12	0.30221	1.30057	0.19349	0.00533	0.03213	0.97437
S12-D12	0.18985	0.91309	0.36125	0.18511	1.24962	0.21152
S13-D12	0.61736	3.24173	0.00120	-0.41148	-2.82377	0.00477
S13-D14	0.92283	5.05003	0.00000	-1.29499	-6.21760	0.00000
S15-D12	0.47496	2.40988	0.01601	-0.22971	-1.22471	0.22076
S15-D13	0.36324	1.78722	0.07399	-0.30505	-2.18125	0.02923
S15-D14	0.54596	3.14002	0.00170	0.05263	0.31737	0.75098
S15-D19	0.27840	1.33329	0.18252	-0.12260	-0.57392	0.56606
S17-D15	0.35573	1.86447	0.06234	-0.54051	-2.77845	0.00549
S20-D13	0.11366	0.55602	0.57823	0.44198	2.38489	0.01714

Note. Beta denotes the GLM regression coefficient. t and p values correspond to the contrast of interest (listen > baseline). Values are rounded to five decimal places. Only channels retained following the scalp coupling index (SCI) criterion are shown.

Table S50

Single-Subject GLM Results for HbO and HbR During the Speak Task (Participant pp16)

Channel	HbO Beta	HbO t	HbO p	HbR Beta	HbR t	HbR p
S6-D15	1.78975	6.88738	0.00000	-0.22501	-0.96769	0.33326
S8-D16	0.77389	3.63805	0.00028	0.01884	0.10227	0.91855
S13-D14	0.83725	3.20928	0.00134	0.06496	0.33576	0.73707
S14-D15	1.34067	5.39304	0.00000	-0.36919	-1.63421	0.10230
S16-D19	0.82242	3.90269	0.00010	0.19908	1.03676	0.29992
S17-D11	0.84860	3.97195	0.00007	0.28071	1.51291	0.13039

Note. Beta denotes the GLM regression coefficient. t and p values correspond to the contrast of interest (listen > baseline). Values are rounded to five decimal places. Only channels retained following the scalp coupling index (SCI) criterion are shown.

Table S51

Single-Subject GLM Results for HbO and HbR During the Speak Task (Participant pp17)

Channel	HbO Beta	HbO t	HbO p	HbR Beta	HbR t	HbR p
S3-D3	-0.15989	-0.85442	0.39293	0.44888	1.88326	0.05975
S3-D5	-0.25102	-1.30653	0.19146	-0.20871	-1.07184	0.28387
S4-D3	-0.88184	-3.08779	0.00203	-0.22529	-0.88582	0.37578
S4-D5	-0.10677	-0.38529	0.70005	-0.25027	-1.04156	0.29769
S4-D6	-0.09932	-0.37813	0.70536	0.10855	0.44111	0.65916
S5-D5	0.80783	3.47668	0.00051	-0.76126	-3.32988	0.00088
S5-D7	0.54154	3.05975	0.00223	-0.67130	-4.72889	0.00000
S7-D7	0.32680	1.64686	0.09968	-0.20128	-1.06078	0.28886
S7-D16	-0.01446	-0.08294	0.93391	-0.47905	-2.37788	0.01747
S11-D10	0.32695	1.45452	0.14589	-0.62394	-3.00567	0.00267
S11-D12	0.33802	1.95121	0.05111	-0.03613	-0.15331	0.87816
S12-D12	-0.17869	-0.86262	0.38841	-0.10547	-0.46999	0.63839
S12-D13	0.36198	1.44928	0.14735	-0.58782	-2.55240	0.01074
S13-D12	0.74129	3.30817	0.00095	0.36819	1.56552	0.11755
S13-D14	0.42155	2.48785	0.01290	0.07016	0.41512	0.67808
S15-D14	-0.08032	-0.42641	0.66983	-0.19702	-1.39664	0.16261

Channel	HbO Beta	HbO t	HbO p	HbR Beta	HbR t	HbR p
S17-D11	-0.79533	-2.69884	0.00699	-0.01553	-0.05690	0.95463
S18-D6	0.54778	2.19353	0.02833	0.16509	0.72041	0.47132
S20-D13	0.41531	1.69264	0.09061	-0.04361	-0.19446	0.84583

Note. Beta denotes the GLM regression coefficient. t and p values correspond to the contrast of interest (listen > baseline). Values are rounded to five decimal places. Only channels retained following the scalp coupling index (SCI) criterion are shown.

Table S52

Single-Subject GLM Results for HbO and HbR During the Speak Task (Participant pp18)

Channel	HbO Beta	HbO t	HbO p	HbR Beta	HbR t	HbR p
S3-D5	0.07328	0.29545	0.76766	-1.26440	-6.05559	0.00000
S4-D3	0.79661	4.20023	0.00003	-0.05789	-0.27568	0.78281
S4-D5	1.00517	5.90108	0.00000	-0.78674	-4.07994	0.00005
S4-D6	0.82825	4.45629	0.00001	-0.19985	-0.93282	0.35097
S5-D5	1.18154	4.98381	0.00000	-1.10313	-5.44760	0.00000
S5-D7	0.31877	1.29918	0.19396	-0.29772	-1.43798	0.15052
S6-D4	0.39298	1.84772	0.06472	-0.10782	-0.43249	0.66541
S6-D6	0.43188	1.93968	0.05249	0.32475	1.53662	0.12447
S6-D15	0.64060	2.94274	0.00327	-1.03621	-4.34928	0.00001
S6-D17	-0.22574	-0.98368	0.32534	-0.27748	-1.36280	0.17303
S7-D5	0.57228	2.79397	0.00523	-0.17366	-0.82626	0.40871
S8-D16	0.87699	4.78766	0.00000	0.92180	4.46860	0.00001
S11-D10	0.26867	1.22473	0.22075	-0.26036	-1.18744	0.23513
S11-D12	0.58367	2.93271	0.00338	-1.02531	-4.60140	0.00000
S12-D12	0.56270	2.80603	0.00504	-0.92281	-4.42920	0.00001
S12-D13	0.48210	1.94685	0.05163	0.31588	1.25726	0.20874
S13-D12	0.69745	3.17989	0.00149	-0.51064	-2.41745	0.01568
S13-D14	-0.31899	-1.41770	0.15636	-0.70246	-3.32838	0.00088
S15-D12	0.16598	0.70236	0.48250	-0.30734	-1.32278	0.18599
S15-D13	0.22135	0.92920	0.35285	-0.30894	-1.14154	0.25372
S15-D14	-0.28652	-1.29639	0.19492	-0.00581	-0.02677	0.97864

Channel	HbO Beta	HbO t	HbO p	HbR Beta	HbR t	HbR p
S16-D19	0.38059	1.78407	0.07449	-0.36080	-2.13997	0.03242
S17-D11	0.97066	4.53489	0.00001	0.55471	2.61586	0.00894
S18-D6	0.40221	2.23427	0.02552	-0.01606	-0.08264	0.93414
S20-D13	0.20080	0.86149	0.38902	-1.03441	-4.56043	0.00001

Note. Beta denotes the GLM regression coefficient. t and p values correspond to the contrast of interest (listen > baseline). Values are rounded to five decimal places. Only channels retained following the scalp coupling index (SCI) criterion are shown.

Table S53

Single-Subject GLM Results for HbO and HbR During the Speak Task (Participant pp19)

Channel	HbO Beta	HbO t	HbO p	HbR Beta	HbR t	HbR p
S3-D3	-0.07073	-0.23271	0.81600	-0.46998	-1.94364	0.05202
S3-D5	-0.09982	-0.47218	0.63683	-0.27296	-1.26693	0.20526
S4-D3	0.29002	0.92099	0.35712	-0.36418	-1.52713	0.12682
S4-D4	0.38815	1.24437	0.21345	-0.54028	-2.09949	0.03584
S4-D5	0.13287	0.45609	0.64835	-0.05416	-0.25343	0.79995
S4-D6	0.43822	1.46714	0.14242	-0.50332	-1.91914	0.05505
S5-D5	-0.38592	-2.07630	0.03794	0.21561	0.91769	0.35884
S5-D7	-0.39944	-2.03175	0.04225	0.13533	0.54993	0.58240
S6-D6	0.00841	0.03317	0.97354	0.05158	0.21027	0.83347
S6-D15	-0.01613	-0.05544	0.95579	-0.12955	-0.51950	0.60344
S6-D17	0.03057	0.11413	0.90914	0.22910	0.78582	0.43203
S7-D5	-0.00827	-0.04113	0.96720	-0.01454	-0.05806	0.95370
S7-D6	0.08158	0.31076	0.75600	0.10415	0.41634	0.67718
S7-D7	-0.39189	-2.16800	0.03022	-0.05216	-0.33942	0.73432
S7-D16	-0.13163	-0.68373	0.49419	-0.23530	-1.14043	0.25418
S8-D16	-0.17308	-0.64694	0.51771	0.25284	1.09494	0.27362
S11-D10	-0.02296	-0.07453	0.94059	-0.16461	-0.54596	0.58512
S11-D12	0.19172	0.72596	0.46791	-0.52765	-2.51564	0.01192
S12-D10	-0.03723	-0.11750	0.90647	-0.14345	-0.50836	0.61123
S12-D12	0.12496	0.41761	0.67626	-0.22230	-0.79643	0.42583

Channel	HbO Beta	HbO t	HbO p	HbR Beta	HbR t	HbR p
S13-D12	0.06953	0.35975	0.71906	-0.24390	-1.00489	0.31502
S13-D14	0.03879	0.19450	0.84580	-0.00030	-0.00167	0.99866
S14-D18	-0.06157	-0.23476	0.81440	0.10484	0.34106	0.73308
S15-D12	0.14480	0.80618	0.42019	-0.43248	-2.22127	0.02639
S15-D14	-0.15141	-0.65584	0.51197	-0.03663	-0.14800	0.88235
S15-D19	-0.18362	-1.01040	0.31237	-0.35474	-2.14520	0.03200
S16-D19	-0.17138	-0.85923	0.39027	-0.27067	-1.37038	0.17065
S18-D6	0.01004	0.03359	0.97320	0.10949	0.40740	0.68374

Note. Beta denotes the GLM regression coefficient. t and p values correspond to the contrast of interest (listen > baseline). Values are rounded to five decimal places. Only channels retained following the scalp coupling index (SCI) criterion are shown.

Table S54

Single-Subject GLM Results for HbO and HbR During the Speak Task (Participant pp20)

Channel	HbO Beta	HbO t	HbO p	HbR Beta	HbR t	HbR p
S3-D3	-0.29940	-1.19748	0.23119	-0.72548	-3.12429	0.00180
S3-D5	0.25456	1.06932	0.28499	-0.79495	-2.98874	0.00282
S4-D3	0.31795	1.23491	0.21694	-0.64874	-2.64857	0.00812
S4-D4	-0.77231	-2.83073	0.00467	-0.23707	-1.01907	0.30823
S4-D5	0.79631	3.10144	0.00194	-1.45637	-6.00322	0.00000
S4-D6	-0.40031	-1.73962	0.08201	-1.08409	-5.02029	0.00000
S5-D5	0.38478	2.10440	0.03541	-0.39696	-2.00390	0.04515
S5-D7	0.47286	2.36300	0.01818	0.45055	2.22363	0.02623
S6-D6	-1.30413	-5.84557	0.00000	1.04579	4.80484	0.00000
S6-D17	-0.40734	-1.74685	0.08074	-0.11170	-0.47955	0.63157
S7-D5	0.08205	0.36247	0.71702	-1.13351	-4.98824	0.00000
S7-D6	0.29560	1.31318	0.18920	0.57824	2.85909	0.00427
S7-D7	-0.35471	-1.79535	0.07267	-0.49283	-2.20062	0.02782
S7-D16	-0.00701	-0.03131	0.97503	1.52317	7.48159	0.00000
S10-D11	-0.52390	-2.11998	0.03407	0.25285	1.07290	0.28338
S11-D10	0.14453	0.58704	0.55721	-0.22627	-0.92532	0.35485

Channel	HbO Beta	HbO t	HbO p	HbR Beta	HbR t	HbR p
S11-D12	0.29953	1.34137	0.17988	-0.52004	-2.70715	0.00682
S12-D11	-0.10790	-0.41873	0.67543	0.30071	1.11225	0.26610
S12-D12	0.55005	2.64237	0.00827	0.06235	0.29375	0.76897
S12-D13	0.77275	3.35622	0.00080	-0.79807	-3.55664	0.00038
S13-D12	-0.93929	-4.53793	0.00001	-1.06338	-5.49695	0.00000
S13-D14	-0.61796	-2.87156	0.00411	-1.55703	-7.73115	0.00000
S14-D13	0.11691	0.53433	0.59315	-0.82990	-3.88101	0.00011
S15-D12	-0.85165	-3.75973	0.00017	-0.64891	-3.66203	0.00025
S15-D13	-1.08692	-4.18500	0.00003	-0.60201	-3.63772	0.00028
S15-D14	-0.13180	-0.57300	0.56668	-1.29067	-6.58059	0.00000
S15-D19	0.03125	0.13772	0.89047	-1.35333	-6.10660	0.00000
S16-D19	0.97971	4.91112	0.00000	0.90473	4.57504	0.00000
S18-D6	-0.89623	-4.41533	0.00001	0.69324	2.78964	0.00530
S20-D13	-0.29001	-1.29732	0.19460	0.29498	1.22028	0.22243

Note. Beta denotes the GLM regression coefficient. t and p values correspond to the contrast of interest (listen > baseline). Values are rounded to five decimal places. Only channels retained following the scalp coupling index (SCI) criterion are shown.

Table S55

Single-Subject GLM Results for HbO and HbR During the Speak Task (Participant pp21)

Channel	HbO Beta	HbO t	HbO p	HbR Beta	HbR t	HbR p
S2-D4	-0.66534	-3.79461	0.00015	0.44250	2.78740	0.00534
S3-D3	-0.73980	-3.14333	0.00168	1.56755	6.38663	0.00000
S3-D5	-0.62868	-2.69647	0.00704	0.44979	1.77644	0.07574
S4-D3	-0.72314	-3.96717	0.00007	0.52072	2.47709	0.01329
S4-D4	-0.62829	-4.06560	0.00005	0.28705	1.76424	0.07777
S4-D5	-0.80680	-4.80750	0.00000	1.22265	4.87412	0.00000
S4-D6	-0.59929	-3.82571	0.00013	-0.26984	-1.55013	0.12119
S5-D5	-0.89292	-3.94535	0.00008	-0.15570	-0.68499	0.49340
S5-D7	-0.83508	-3.66811	0.00025	-0.20341	-0.95428	0.34000
S6-D4	-0.63295	-3.65539	0.00026	-0.37708	-1.93073	0.05359

Channel	HbO Beta	HbO t	HbO p	HbR Beta	HbR t	HbR p
S6-D6	-0.63854	-3.17742	0.00150	0.25279	1.25180	0.21072
S6-D15	-0.55094	-3.00082	0.00271	0.10524	0.45422	0.64970
S6-D17	-0.70661	-3.26184	0.00112	0.81246	3.47808	0.00051
S7-D5	-0.38552	-2.26184	0.02376	-0.67754	-3.24746	0.00117
S7-D6	-0.55396	-3.20915	0.00134	-0.25409	-1.20481	0.22835
S7-D7	-0.07314	-0.29822	0.76555	0.02348	0.11579	0.90782
S7-D16	-0.53938	-2.31939	0.02043	-0.09084	-0.46075	0.64501
S8-D16	-0.72701	-3.39651	0.00069	0.34774	1.81324	0.06987
S10-D11	-0.84367	-4.66464	0.00000	0.39088	1.72880	0.08393
S11-D10	-0.63597	-3.18567	0.00146	-0.07237	-0.37666	0.70645
S11-D12	-0.64384	-3.55294	0.00039	-0.28559	-1.45016	0.14710
S12-D10	-0.70799	-3.99973	0.00006	0.95532	4.07388	0.00005
S12-D11	-0.97297	-4.57929	0.00000	0.08062	0.39924	0.68974
S12-D12	-0.33315	-1.94052	0.05239	-0.51822	-2.89250	0.00384
S12-D13	-0.67388	-2.88333	0.00396	-0.31366	-1.28960	0.19727
S13-D12	-0.40023	-2.05419	0.04003	-0.49958	-2.41902	0.01561
S13-D14	-0.62327	-2.77598	0.00553	-0.36183	-1.98253	0.04749
S14-D11	-0.72350	-2.72619	0.00644	-0.20235	-1.04840	0.29452
S14-D13	-0.90933	-3.58697	0.00034	0.09518	0.50779	0.61163
S14-D15	-0.85661	-4.05315	0.00005	-0.34186	-1.58527	0.11299
S14-D18	-0.82546	-3.68242	0.00023	1.20522	5.10428	0.00000
S15-D12	-0.28502	-1.55706	0.11954	-0.27570	-1.39927	0.16181
S15-D13	-0.48616	-2.23383	0.02555	0.19496	0.95256	0.34087
S15-D14	-0.52190	-2.29872	0.02158	0.35276	1.99396	0.04623
S15-D19	-0.84457	-3.74709	0.00018	1.01830	5.10290	0.00000
S16-D19	-0.52890	-3.30852	0.00095	-0.10590	-0.56800	0.57007
S17-D4	-0.59738	-3.24062	0.00120	-0.25932	-1.21565	0.22420
S17-D11	-0.69423	-3.37330	0.00075	0.22530	1.05768	0.29027
S17-D15	-0.69291	-3.59021	0.00033	-0.56794	-2.49006	0.01282
S18-D6	-0.34401	-1.72367	0.08485	0.50838	2.33011	0.01985

Channel	HbO Beta	HbO t	HbO p	HbR Beta	HbR t	HbR p
S20-D13	-0.90790	-4.32226	0.00002	0.39203	1.86749	0.06191

Note. Beta denotes the GLM regression coefficient. t and p values correspond to the contrast of interest (listen > baseline). Values are rounded to five decimal places. Only channels retained following the scalp coupling index (SCI) criterion are shown.

Table S56

Single-Subject GLM Results for HbO and HbR During the Speak Task (Participant pp22)

Channel	HbO Beta	HbO t	HbO p	HbR Beta	HbR t	HbR p
S2-D4	0.44705	2.09237	0.03648	0.57432	2.54632	0.01093
S3-D3	-0.13053	-0.50506	0.61355	0.82861	4.36448	0.00001
S3-D5	0.35480	1.70759	0.08780	-0.14841	-0.92337	0.35588
S4-D3	0.12708	0.51171	0.60889	1.27749	5.49132	0.00000
S4-D4	0.35061	1.75332	0.07963	0.81093	3.94855	0.00008
S4-D5	0.50418	2.41826	0.01564	0.50130	2.28902	0.02214
S4-D6	0.72424	3.26512	0.00110	0.60887	2.53260	0.01136
S5-D5	0.07617	0.49480	0.62077	-0.52799	-3.75216	0.00018
S5-D7	0.09862	0.55823	0.57672	-0.06019	-0.38132	0.70298
S6-D4	0.49049	2.34366	0.01915	0.19011	1.16017	0.24606
S6-D6	1.10797	4.46905	0.00001	0.02130	0.10049	0.91996
S6-D15	0.64229	2.54530	0.01096	-0.00138	-0.00914	0.99271
S6-D17	1.12970	4.81000	0.00000	-0.02311	-0.12437	0.90103
S7-D5	0.50148	3.10198	0.00194	-0.37392	-3.11829	0.00183
S7-D6	0.75223	3.28973	0.00101	-0.42673	-2.53275	0.01136
S7-D7	0.81937	5.08807	0.00000	-0.51001	-3.59091	0.00033
S7-D16	0.65915	3.82220	0.00013	-0.33315	-2.47582	0.01334
S10-D11	0.15718	0.72062	0.47119	0.34485	1.64647	0.09976
S11-D10	0.09814	0.40925	0.68238	1.15587	4.95064	0.00000
S11-D12	0.18042	1.02479	0.30553	0.14110	1.23243	0.21787
S12-D10	0.52069	2.48000	0.01318	0.38209	1.87881	0.06035
S12-D11	0.46932	1.97845	0.04795	0.52226	2.94506	0.00325
S12-D12	0.57800	2.57624	0.01003	-0.51368	-2.61523	0.00895

Channel	HbO Beta	HbO t	HbO p	HbR Beta	HbR t	HbR p
S12-D13	0.28087	1.34077	0.18008	0.49176	2.44008	0.01473
S13-D12	-0.19390	-1.54037	0.12356	-0.25379	-1.79540	0.07267
S13-D14	-0.24015	-1.53012	0.12608	-0.15278	-1.14261	0.25328
S14-D11	0.93574	4.13708	0.00004	0.02202	0.13318	0.89406
S14-D13	0.99248	4.08898	0.00004	0.39235	2.88757	0.00391
S14-D15	0.91178	4.95495	0.00000	0.04027	0.23340	0.81547
S14-D18	0.89141	3.70277	0.00022	0.20367	1.12298	0.26152
S15-D12	0.11440	0.90590	0.36505	-0.29924	-2.91154	0.00362
S15-D14	0.04723	0.31521	0.75262	-0.28276	-2.01999	0.04346
S15-D19	0.20814	1.55801	0.11932	-0.39218	-3.31688	0.00092
S17-D4	0.83425	4.16845	0.00003	-0.00182	-0.01220	0.99027
S17-D11	0.52634	2.60710	0.00917	0.12883	0.71429	0.47509
S17-D15	0.82180	3.85335	0.00012	0.18339	1.13745	0.25543
S18-D6	0.60913	2.51707	0.01188	-0.00616	-0.02735	0.97818
S20-D13	1.01991	4.12361	0.00004	-0.10476	-0.59066	0.55479

Note. Beta denotes the GLM regression coefficient. t and p values correspond to the contrast of interest (listen > baseline). Values are rounded to five decimal places. Only channels retained following the scalp coupling index (SCI) criterion are shown.

Table S57

Single-Subject GLM Results for HbO and HbR During the Speak Task (Participant pp23)

Channel	HbO Beta	HbO t	HbO p	HbR Beta	HbR t	HbR p
S3-D3	0.85801	3.56822	0.00036	0.14924	0.64338	0.52002
S3-D5	0.48798	2.42404	0.01539	-0.41701	-1.80022	0.07190
S4-D3	1.36925	5.10199	0.00000	0.60596	2.40286	0.01631
S4-D4	1.63109	6.45551	0.00000	1.01452	4.26912	0.00002
S4-D5	0.98138	4.15071	0.00003	-0.61139	-2.50324	0.01235
S4-D6	1.21145	4.84581	0.00000	-0.47494	-1.94620	0.05170
S6-D4	1.20398	4.58549	0.00000	0.19097	0.69222	0.48884
S6-D6	0.72605	2.83423	0.00462	-0.71234	-2.85451	0.00433
S7-D5	1.93074	8.91998	0.00000	-0.81594	-3.61903	0.00030

Channel	HbO Beta	HbO t	HbO p	HbR Beta	HbR t	HbR p
S7-D6	1.81181	8.79854	0.00000	-0.96591	-4.24546	0.00002
S7-D7	0.89370	3.88063	0.00011	-0.71385	-3.20991	0.00134
S7-D16	2.01209	10.02175	0.00000	-0.86015	-3.71542	0.00021
S10-D11	1.23969	4.47650	0.00001	0.31878	1.21568	0.22418
S11-D10	0.92968	3.32655	0.00089	0.54945	2.34933	0.01886
S11-D12	0.38379	1.64939	0.09915	0.22893	0.90294	0.36661
S12-D10	1.03683	3.92316	0.00009	0.71942	2.89589	0.00380
S12-D11	1.53026	5.65935	0.00000	0.36602	1.79914	0.07207
S12-D12	0.47936	2.26200	0.02375	0.30454	1.57501	0.11533
S12-D13	0.70206	2.96626	0.00303	0.34333	1.83569	0.06648
S13-D14	0.61972	2.71827	0.00659	-0.66627	-3.08231	0.00207
S14-D13	0.54985	2.06116	0.03935	1.19713	4.54726	0.00001
S14-D15	0.09019	0.31071	0.75604	-0.84418	-3.25077	0.00116
S14-D18	1.04327	3.46215	0.00054	0.34268	1.25042	0.21122
S15-D12	0.53398	2.68563	0.00727	-0.94630	-4.00861	0.00006
S15-D13	0.90663	3.51922	0.00044	0.34697	1.35423	0.17574
S15-D14	0.94386	4.23233	0.00002	-0.91984	-3.62197	0.00030
S15-D19	1.19386	5.19506	0.00000	-0.94711	-3.49796	0.00047
S17-D4	0.73722	2.72559	0.00645	-0.50778	-1.90029	0.05747
S20-D13	0.28711	1.04711	0.29511	0.89445	3.92189	0.00009

Note. Beta denotes the GLM regression coefficient. t and p values correspond to the contrast of interest (listen > baseline). Values are rounded to five decimal places. Only channels retained following the scalp coupling index (SCI) criterion are shown.

Table S58

Single-Subject GLM Results for HbO and HbR During the Speak Task (Participant pp24)

Channel	HbO Beta	HbO t	HbO p	HbR Beta	HbR t	HbR p
S4-D5	0.59988	2.32097	0.02034	-1.41417	-7.75193	0.00000
S5-D7	-0.13523	-0.69031	0.49004	-0.37589	-2.09276	0.03643
S6-D15	0.36841	1.64604	0.09983	-1.07171	-5.92284	0.00000
S7-D5	0.32462	1.45143	0.14674	-0.46992	-1.84858	0.06459

Channel	HbO Beta	HbO t	HbO p	HbR Beta	HbR t	HbR p
S7-D6	0.46783	1.82300	0.06838	-0.56409	-2.90145	0.00373
S7-D7	0.37128	2.49938	0.01248	-0.38704	-1.88853	0.05903
S7-D16	0.27165	1.41669	0.15665	-0.00418	-0.02663	0.97876
S8-D16	0.31558	1.41281	0.15779	0.40778	2.34877	0.01888
S11-D12	0.08948	0.40041	0.68888	-0.47516	-2.26800	0.02338
S12-D12	-0.03116	-0.11133	0.91136	-0.37382	-2.85467	0.00433
S13-D12	0.06454	0.23926	0.81091	-0.77450	-4.19990	0.00003
S13-D14	0.06397	0.25876	0.79583	-0.63524	-2.96243	0.00307
S14-D13	0.94119	3.47711	0.00051	-0.48242	-2.23721	0.02533
S14-D15	0.21095	0.89267	0.37208	-0.34447	-1.88263	0.05982
S15-D12	0.19878	0.81820	0.41329	-0.77278	-3.46017	0.00055
S15-D13	0.11183	0.43275	0.66522	-0.74938	-5.98959	0.00000
S15-D14	0.08010	0.50340	0.61471	-0.20937	-1.16333	0.24476
S15-D19	0.20169	0.91984	0.35771	-0.86438	-3.63191	0.00028
S17-D4	0.27866	1.23279	0.21773	0.26289	0.94909	0.34263
S17-D11	0.56477	2.62015	0.00882	-0.64830	-2.47699	0.01329
S18-D6	0.75793	3.66287	0.00025	-0.45031	-2.29292	0.02190
S20-D13	-0.21024	-0.78828	0.43058	-0.59845	-4.55601	0.00001

Note. Beta denotes the GLM regression coefficient. t and p values correspond to the contrast of interest (listen > baseline). Values are rounded to five decimal places. Only channels retained following the scalp coupling index (SCI) criterion are shown.

Table S59

Single-Subject GLM Results for HbO and HbR During the Speak Task (Participant pp25)

Channel	HbO Beta	HbO t	HbO p	HbR Beta	HbR t	HbR p
S2-D4	0.94842	3.87903	0.00011	-0.57206	-2.81328	0.00493
S3-D5	-0.28319	-1.28053	0.20044	-0.40523	-1.82781	0.06766
S4-D3	1.38646	7.04420	0.00000	-0.27832	-1.22724	0.21981
S4-D4	1.31815	6.66184	0.00000	-0.33781	-1.37634	0.16880
S4-D5	0.43612	1.72597	0.08443	-0.18324	-0.86835	0.38526
S4-D6	1.46468	7.14336	0.00000	-0.67625	-2.77581	0.00553

Channel	HbO Beta	HbO t	HbO p	HbR Beta	HbR t	HbR p
S5-D5	0.05571	0.24625	0.80550	0.26222	1.47445	0.14044
S5-D7	-0.10940	-0.51489	0.60666	-0.20148	-1.01034	0.31239
S6-D4	1.00272	4.60738	0.00000	-0.53045	-2.43168	0.01507
S6-D6	0.27708	1.17866	0.23861	-0.79939	-5.61985	0.00000
S6-D15	0.96159	4.14733	0.00003	-0.37715	-1.74825	0.08050
S6-D17	0.00179	0.00808	0.99355	-0.21522	-1.14285	0.25317
S7-D5	0.67712	3.09775	0.00196	-0.00450	-0.01816	0.98551
S7-D6	0.05220	0.20158	0.84026	-0.39748	-2.04518	0.04091
S7-D7	0.59639	2.97808	0.00292	0.54179	2.59246	0.00957
S7-D16	0.25957	1.09378	0.27412	-0.27985	-1.38934	0.16481
S8-D16	-0.22402	-0.98798	0.32322	0.12828	0.58951	0.55555
S10-D11	0.77244	4.85986	0.00000	-0.66677	-2.43534	0.01492
S11-D10	0.32393	1.30185	0.19305	-0.48814	-2.46591	0.01371
S11-D12	0.76861	3.25465	0.00115	-1.34032	-6.15533	0.00000
S12-D10	0.99520	4.45191	0.00001	-0.59647	-3.41080	0.00065
S12-D11	1.12915	5.41558	0.00000	-0.51727	-3.58627	0.00034
S12-D12	0.54691	2.08800	0.03686	0.14260	0.61321	0.53977
S12-D13	0.94154	3.48604	0.00050	-0.14736	-0.81655	0.41423
S13-D12	0.53419	2.33843	0.01942	-0.85630	-4.13701	0.00004
S13-D14	-0.41332	-1.69257	0.09062	-0.07693	-0.29738	0.76620
S14-D11	0.52858	3.24906	0.00117	-0.63439	-4.58684	0.00000
S14-D13	1.06084	5.09242	0.00000	-0.62086	-3.08216	0.00207
S14-D15	0.52655	3.82813	0.00013	-0.61433	-3.30742	0.00095
S14-D18	0.61848	3.40908	0.00066	-0.66304	-3.26321	0.00111
S15-D12	0.45059	2.06586	0.03891	-0.15005	-0.80263	0.42224
S15-D13	1.11663	4.73284	0.00000	-1.21423	-8.79095	0.00000
S15-D14	0.51842	2.64194	0.00828	0.56232	3.14154	0.00169
S15-D19	0.39376	1.96561	0.04942	-0.26777	-1.42113	0.15536
S16-D19	0.16076	0.64552	0.51863	-0.21770	-1.12749	0.25960
S17-D4	1.15017	5.26741	0.00000	-0.05201	-0.35761	0.72066

Channel	HbO Beta	HbO t	HbO p	HbR Beta	HbR t	HbR p
S17-D11	1.00693	3.93205	0.00009	-0.26536	-3.90440	0.00010
S17-D15	0.95565	3.76422	0.00017	-0.08984	-0.75762	0.44873
S18-D6	-0.31112	-1.31433	0.18882	-0.15645	-1.09614	0.27309
S20-D13	0.73672	3.03021	0.00246	-0.81867	-4.59890	0.00000

Note. Beta denotes the GLM regression coefficient. t and p values correspond to the contrast of interest (listen > baseline). Values are rounded to five decimal places. Only channels retained following the scalp coupling index (SCI) criterion are shown.

Table S60

Single-Subject GLM Results for HbO and HbR During the Speak Task (Participant pp26)

Channel	HbO Beta	HbO t	HbO p	HbR Beta	HbR t	HbR p
S3-D5	0.59076	2.27749	0.02281	-0.69735	-2.69996	0.00696
S4-D3	1.17806	5.21726	0.00000	-0.07387	-0.28875	0.77279
S4-D4	0.860377999	3.45560	0.00055	0.22325	0.90911	0.36335
S4-D5	0.97498	4.16164	0.00003	0.21844	0.79864	0.42455
S4-D6	1.32137	5.89975	0.00000	-0.29760	-1.33882	0.18071
S5-D5	0.46748	2.11298	0.03466	-0.15531	-0.67512	0.49964
S5-D7	0.27802	1.19772	0.23109	0.31296	1.55993	0.11885
S6-D6	0.70728	2.64801	0.00813	-0.59466	-2.61582	0.00893
S6-D15	0.56662	2.56972	0.01021	-0.19015	-0.97548	0.32938
S6-D17	0.84228	3.80078	0.00015	-0.68646	-3.26832	0.00109
S7-D5	0.39190	1.69357	0.09042	0.33410	1.27407	0.20271
S7-D7	0.68639	3.92649	0.00009	0.25855	1.34644	0.17824
S8-D16	-0.60121	-2.89083	0.00386	0.52401	2.65449	0.00797
S11-D10	0.72532	2.85400	0.00434	0.53341	2.05340	0.04010
S11-D12	1.03593	4.55237	0.00001	-0.72946	-2.94518	0.00325
S12-D10	0.75944	3.20501	0.00136	-0.04007	-0.16063	0.87239
S12-D12	1.14715	4.78474	0.00000	-0.55433	-2.40697	0.01613
S12-D13	1.08068	5.16655	0.00000	-0.59449	-2.54365	0.01101
S13-D12	1.58299	7.53724	0.00000	-1.37198	-6.62385	0.00000
S13-D14	0.33297	1.60583	0.10839	0.04611	0.23685	0.81279

Channel	HbO Beta	HbO t	HbO p	HbR Beta	HbR t	HbR p
S14-D13	0.90040	3.94940	0.00008	-0.85381	-3.90505	0.00010
S14-D18	0.63657	3.05801	0.00224	-0.43287	-2.14020	0.03240
S15-D12	0.80067	3.42192	0.00063	-0.18874	-0.72315	0.46963
S15-D13	0.96180	4.74911	0.00000	-0.84380	-3.90129	0.00010
S15-D14	0.92168	5.73697	0.00000	-0.04723	-0.26164	0.79361
S15-D19	0.70582	3.27235	0.00108	-0.06474	-0.27862	0.78055
S16-D19	-0.30702	-1.78863	0.07375	-0.62234	-2.53230	0.01137
S18-D6	0.79428	3.12055	0.00182	0.07774	0.34059	0.73343

Note. Beta denotes the GLM regression coefficient. t and p values correspond to the contrast of interest (listen > baseline). Values are rounded to five decimal places. Only channels retained following the scalp coupling index (SCI) criterion are shown.

Table S61

Single-Subject GLM Results for HbO and HbR During the Speak Task (Participant pp27)

Channel	HbO Beta	HbO t	HbO p	HbR Beta	HbR t	HbR p
S3-D5	-0.17717	-0.81923	0.41271	-0.54464	-2.81377	0.00492
S4-D3	-0.05300	-0.20358	0.83869	-0.40411	-1.46580	0.14279
S4-D5	0.486836152	2.36700	0.01798	-0.73776	-3.84419	0.00012
S4-D6	0.07140	0.33612	0.73680	-0.40883	-2.68981	0.00718
S5-D5	0.19432	0.85588	0.39212	-0.18145	-0.84162	0.40006
S5-D7	0.70761	3.28537	0.00103	-0.54370	-3.28395	0.00103
S6-D6	-0.58766	-2.76345	0.00575	-0.06976	-0.37426	0.70823
S6-D15	-0.57189	-2.72575	0.00645	-0.20188	-0.91584	0.35981
S7-D5	0.72107	3.71782	0.00020	-1.29469	-5.37208	0.00000
S7-D7	0.25315	1.37545	0.16908	-0.09598	-0.45486	0.64924
S8-D16	0.46934	2.33524	0.01958	0.31514	1.56176	0.11843
S11-D10	-0.35443	-1.60275	0.10908	-0.22562	-0.99936	0.31769
S11-D12	0.88230	6.70602	0.00000	-1.31476	-6.22260	0.00000
S12-D12	0.54464	3.09526	0.00198	-1.01034	-4.92684	0.00000
S12-D13	-0.20363	-1.03427	0.30108	-0.66311	-3.00385	0.00268
S13-D12	0.35409	1.70470	0.08833	-1.00880	-6.08369	0.00000

Channel	HbO Beta	HbO t	HbO p	HbR Beta	HbR t	HbR p
S13-D14	0.39558	2.02963	0.04247	-0.74648	-4.63103	0.00000
S15-D12	0.98216	5.18698	0.00000	-1.34139	-6.46608	0.00000
S15-D13	0.00000	0.00002	0.99999	-1.43108	-6.66977	0.00000
S15-D14	0.33247	1.62141	0.10502	-0.78500	-5.55385	0.00000
S15-D19	0.36612	1.68544	0.09199	-0.86705	-5.75735	0.00000
S16-D19	0.55801	2.67756	0.00745	-0.17976	-0.79716	0.42541
S17-D15	-0.43013	-1.60207	0.10923	-0.25673	-1.47963	0.13906
S18-D6	-0.02721	-0.11854	0.90565	-0.43953	-1.67247	0.09452
S20-D13	0.65970	3.76179	0.00017	-1.28293	-5.37158	0.00000

Note. Beta denotes the GLM regression coefficient. t and p values correspond to the contrast of interest (listen > baseline). Values are rounded to five decimal places. Only channels retained following the scalp coupling index (SCI) criterion are shown.
